# Weak associations between pubertal development and psychiatric and behavioral problems

**DOI:** 10.1038/tp.2017.63

**Published:** 2017-04-18

**Authors:** E Smith-Woolley, K Rimfeld, R Plomin

**Affiliations:** 1 King's College London, MRC Social, Genetic and Developmental Psychiatry Centre, Institute of Psychiatry, Psychology and Neuroscience, London, UK

## Abstract

Pubertal development has been associated with adverse outcomes throughout adolescence and adulthood. However, much of the previous literature has categorized outcome variables and pubertal timing measures for ease of mean difference or odds ratio interpretation. We use a UK-representative sample of over 5000 individuals drawn from the Twins Early Development Study to extend this literature by adopting an individual differences approach and emphasizing effect sizes. We investigate a variety of psychiatric and behavioral measures collected longitudinally at ages 11, 14 and 16, for multiple raters and for males and females separately. In addition, we use two measures of pubertal development: the Pubertal Development Scale at each age, as well as the age of menarche for girls. We found that pubertal development, however assessed, was linearly associated with a range of psychiatric and behavioral outcomes; however, the effect sizes of these associations were modest for both males and females with most correlations between −0.10 and 0.10. Our systematic analysis of associations between pubertal development, and psychiatric and behavioral problems is the most comprehensive to date. The results showing linearity of the effects of pubertal development support an individual differences approach, treating both pubertal development and associated outcomes as continuous rather than categorical variables. We conclude that pubertal development explains little variance in psychiatric and behavioral outcomes (<1% on average). The small effect sizes indicate that the associations are weak and should not warrant major concern at least in non-clinical populations.

## Introduction

Puberty is a developmental milestone, marked by major physical, hormonal, cognitive and social changes. For girls, reaching puberty early has been reported to be a risk factor for a range of psychiatric and behavioral problems during adolescence, including depression,^1, 2, 3, 4, 5, 6, 7^ anxiety,^1, 6^ conduct disorder,^3, 8, 9^ eating disorder^6, 10, 11^ and risky behaviors, including substance use^12, 13^ (for reviews, see Copeland *et al.*,^9^ Brooks-Gunn *et al.*,^14^ Graber,^15^ Negriff and Susman,^16^ and Paus *et al.*^17^). In addition, early pubertal development has also been associated with longer-term outcomes, persisting into adulthood.^4, 17^ However, this research has typically focused on the statistical significance of group differences rather than taking an individual differences approach that focuses on effect sizes, not just statistical significance. That is, pubertal development scores have been used to categorize children into early, average and late groups, resulting in the loss of valuable information within groups. The present study takes an individual differences approach, treating measures as continuous rather than discrete. Furthermore, we estimate both linear and nonlinear models to assess the shape and effect size of associations between pubertal development, and behavioral and psychiatric traits over a 5-year period and compare them to traditional mean differences results. Most pubertal research to date has been conducted with girls; here we consider the relationship between pubertal development and psychiatric and behavioral problems for both boys and girls using self-reported and parent-reported outcome measures over three ages (ages 11, 14 and 16).

There are several theoretical explanations for the association between pubertal development, and behavioral and psychiatric problems both concurrently and over time.^18^ One is the developmental readiness hypothesis (or early-maturational timing hypothesis), which posits that it is the gap between physical and psychosocial development that places adolescents at a risk for developing these problems.^14, 19^ Early maturers are faced with demanding social, physical and hormonal changes, whereas their emotional and cognitive development lags behind. This disparity in development, combined with fewer age-appropriate role models, is thought to leave early maturers vulnerable to current and future problems.^1, 7, 20, 21^ Because girls typically enter puberty before boys, early-maturing girls are thought to be the most affected by the developmental gap, being both early compared to boys and early compared to same-sex peers.^16^ The second explanation is the maturational deviance hypothesis, which suggests that the risk for developing behavioral and psychiatric problems comes from any substantial departure from the norm—either early or late.^22^ In this way, both early-maturing girls and late-maturing boys would be most at risk for behavioral and psychiatric problems, because they are at the developmental extremes. Although less is known about the effects of pubertal development in boys, some research suggests that going through puberty late compared to same-sex peers is associated with psychiatric and behavioral problems.^3, 4, 15^ Furthermore, these problems may not just affect adolescents, but associations between pubertal deviance, and psychiatric and behavioral problems may also persist into adulthood, with certain traits self-perpetuating.^4, 9, 17^

Although both pubertal development theories propose similar effects for early pubertal development, the developmental hypothesis assumes a linear relationship from early to late pubertal development, with early maturers having the most problems, decreasing with age. In contrast, the maturation hypothesis proposes a quadratic (or U-shaped) relationship, in which both early- and late-developing individuals have more problems.

Both theories have gained some empirical support; however, this support is largely based on significant mean differences after splitting the sample into groups, usually categorizing the sample into early, on time and late pubertal development or case–control groups. Although dividing samples in this way may simplify results as risk ratios or mean differences and facilitate clinical decision-making, this simplicity comes at a cost of loss of information, loss of power and arbitrariness.^23^ Furthermore, by emphasizing significance and often not reporting effect sizes, it can be difficult to compare results between studies and interpret their real-world significance.

The purpose of the present study was to address these limitations of the previous literature. We used a UK-representative sample of over 5000 individuals from the Twins Early Development Study (TEDS)^24^ and looked at 38 outcome measures (composed of over 80 scales and subscales) obtained at ages 11, 14 and 16. To investigate pubertal development and associated outcomes, we used both self- and parent-reported measures to study the relationship between pubertal development, and its links to psychiatric and behavioral problems concurrently and over time for girls and boys separately. We used an individual differences approach, comparing both linear and nonlinear relationships to estimate effect sizes, (in this case the proportion of variance explained). Furthermore, we compared these individual differences results to results of group differences.

## Materials and methods

### Sample

The sampling frame for the present study was TEDS. TEDS is a large, representative sample of 16 000 twin pairs born in England and Wales between 1994 and 1996, and followed from birth to the present day. Although there has been some attrition throughout the years, over 10 000 twin pairs remain in the study, many of whom provide rich behavioral and cognitive data. Importantly, TEDS was and still is a representative sample of England and Wales, the sampling and the representativeness of the sample is described in detail elsewhere.^24, 25^

The present study included three waves of testing when twins were 11 (*M*=11.29, s.d.=0.70), 14 (*M*=14.07, s.d.=0.57) and 16 (*M*=16.32, s.d.=0.68) years old (see Table 1 for the sample size per age and measure). To maintain independence of data, one twin individual was randomly selected out of every twin pair and used in the analysis. Analyses were also repeated on the ‘other' twin in a pair to see whether results replicated, although we acknowledge that this is not an independent sample and only allows for ‘part' replication. Individuals with major medical problems and those who had suffered severe problems at birth or for whom their mothers had severe complications during pregnancy were excluded from the analyses. Written informed consent was given for all participants involved for each wave of data collection.

### Measures

#### Pubertal status

The Pubertal Development Scale (PDS)^26^ was used to assess the development of secondary sexual characteristics, such as growth spurts, body hair growth, skin changes, breast development and menarche in girls, and voice changes and growth of testes in boys. The PDS has been found to be a reliable measure and has high correlations with pubertal stage as rated by healthcare professionals (*r*=0.82–0.86 for agreement within one stage between healthcare professionals and self-report PDS),^27, 28^ and with hormone levels.^28^

Participants filled in the five-item measure at ages 11, 14 and 16 by indicating their pubertal development on a four-point scale from: ‘not yet begun (1)^1^' to ‘completed (4)^4^'. For example, the participants rated their development on items such as ‘Have your breast begun to grow?' for girls or ‘Has your voice begun to change?' for boys. Menarche was rated either ‘yes' or ‘no' and the age of menarche was recorded for those who had reached it. The items were averaged to produce a summary PDS score at each age, in addition to age of menarche measure for girls. These measures were corrected for the mean effects of age at every data collection wave by rescoring the variable as a standardized residual correcting for age to create our pubertal status variable.

#### Categories of relative pubertal development

In line with previous literature,^29^ we categorized pubertal development by scoring those who were +1 s.d. from the mean on the total PDS score as ‘early' and those who scored −1 s.d. from the mean of the PDS as late. This analysis was done separately for girls and boys at ages 11, 14 and 16.

#### Internalizing and externalizing problems

We used all behavioral and psychiatric measures collected in TEDS at ages 11, 14 and 16 from self-report and parent-report questionnaires, collected both online and by mail. These included measures previously associated with pubertal development. Obtaining data on psychiatric outcomes was a major aim of data collection at age 16, which included 80 subscales. To investigate systematically the links with pubertal development, we included all 80 measures and reduced these to 38 scales, which are available at http://www.teds.ac.uk/CMSUploads/Supplementary%20Material%20-%20measures.pdf. As with pubertal development, all internalizing and externalizing measures were corrected for the effects of age so as not to confound the measures—which could otherwise be a function of the age they completed the questionnaire.

Table 1 shows the sample sizes across measures. Although there are ~10 000 twin pairs still in the study, not all of these twins actively participate at every data collection wave. For example, at age 11, there were between 5056 and 5623 individuals, whereas at age 14, there were between 2845 and 3173. This sample size variation between waves is due to the follow-up procedures taken. For example, at age 11, all families selected to participate were allocated callers who reminded families to participate, whereas at age 14, due to the size of the study, only a subsample of families were allocated callers, resulting in lower participation rates. As well as variation between waves, there is also sample size variation within waves, for example, at age 16 the sample size ranges from 1070 to 4837. This is due to variations in the questionnaire battery between the two waves of data collection at age 16. A few of the scales (attention-deficit hyperactivity disorder, eating problems, delinquency and callous unemotional traits) were only included in the first wave of data collection at age 16, which comprised one cohort out of four. These scales were not included in the second wave of data collection that included the other three cohorts. Importantly, this does not affect the representativeness of the sample.

### Analyses

Measures were described in terms of means and standard deviations (s.d.'s). All analyses were conducted using only one twin in a pair so as not to inflate the estimates (and to maintain independence of the data). We also replicated the results by using the other ‘co-twin', although we acknowledge that this does not provide a fully independent replication sample. Correlational analysis was used to estimate the linear relationship between pubertal status and outcome measures, both concurrently, and over time. To test whether the ends (extremes) of the distribution were driving an association, we also tested for quadratic and cubic relationships, by adding these terms in incremental *F* tests and observing the *F*-change statistic and associated *P-*value. All analyses were conducted separately for males and females. We compared these results to a more traditional group differences approach using analysis of variance (ANOVA) with contrasts that compared the impact of early vs normal and late vs normal development on our outcomes. We also compared these group-difference results with previously reported results.

## Results

### Missingness analyses

We studied whether attrition in the sample was a function of pubertal development or psychiatric and behavioral problems by creating dichotomous dummy variables representing missingness status at ages 11, 14 and 16. These variables were used in a series of ANOVAs to test whether missingness at later time points were associated with differences in pubertal status, psychiatric and behavioral problems at earlier time points, or whether missingness at earlier time points were related to differences in future outcome measures.

We found that missingness at age 14 explained <1% of the variance of age 11 measures; the same was true for missingness at age 16 with both age 14 and 11 measures. Furthermore, we found that missingness at age 11 explained <1% in later outcome measures at ages 14 and 16, and that the same was true for missingness at 14 and behavioral, psychiatric and pubertal development variables at age 16. These analyses suggest that earlier pubertal, behavioral or psychiatric measures do not influence later missingness or that early missingness does not influence later outcome measures, suggesting that our findings are not compromised by attrition bias (please see Supplementary Table S1 for these analyses).

### Descriptive statistics

For all measures, means and s.d.'s, as well as variance explained by gender, are presented in Table 1. In line with the literature, boys and girls differed significantly on their PDS scores at every age, with girls' mean pubertal development above their male peers at every age and roughly a whole s.d. above males at age 14, when gender explained up to 22% of the variance in pubertal development. Because variance in pubertal development was greater for girls than boys at all three ages, we used the non-parametric Welch test to compare means. We also found expected mean differences between boys and girls for the behavioral and psychiatric measures. However, gender differences only explained between 1 and 6% of the variance in these outcome measures.

In light of these results, all subsequent analyses were conducted separately for males and females.

**Figure 1:**
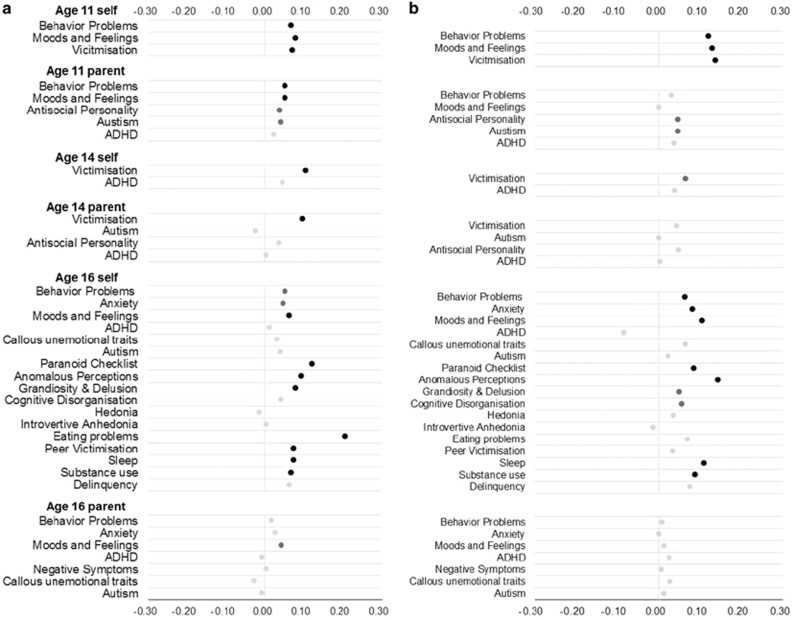
(**a**) Girls - Correlations between pubertal development at age 11 and symptoms of psychiatric and behavioral problems for self- and parent-reported measures at ages 11, 14 and 16. (**b**) Boys - Correlations between pubertal development at age 11 and symptoms of psychiatric and behavioral problems for self- and parent-reported measures at ages 11, 14 and 16 for boys. In both plots, black dots indicate correlations significant at the 0.01 level, dark gray dots indicate correlations significant at the 0.05 level and light gray dots indicate non-significant correlations. ADHD, attention-deficit hyperactivity disorder.

Figure 1 shows correlations between pubertal development at age 11 and symptoms of internalizing and externalizing problems for self- and parent report at ages 11, 14 and 16 separately for girls (Figure 1a) and for boys (Figure 1b). Although pubertal development was found to be associated with adverse outcomes, the effect sizes were small across measures and ages. In line with the literature for females, the largest association was observed between pubertal status at age 11 and eating problems at age 16 (*r*=0.21); however, this effect did not replicate in the co-twin sample, where the correlation was only 0.03 and not significant (see Supplementary Table S2.1). Furthermore, for females, the great majority (92%) of the correlations fell between −0.10 and 0.10. For males, the largest association was between age 11 pubertal status and anomalous perceptions at age 16 (*r*=0.14), where there was also a small effect for the co-twin sample (*r*=0.06). The effects sizes for boys were also modest, with the majority (84%) of correlations falling between −0.10 and 0.10. In summary, these results indicate that puberty generally explains <1% of variance in psychiatric and behavioral outcomes.

The table of correlations and dot-plots for pubertal development at each age with concurrent and future outcomes for boys and girls separately are presented in Supplementary Tables S2.1–S2.7 and Supplementary Figures S1.1–4. The results were very similar across age, gender and rater, with pubertal development explaining a small amount of variance in adverse outcomes both concurrently and over time. Pubertal status was moderately stable across age, with age 11 and age 14 PDS scores correlating 0.49 and age 14 and age 16 correlating 0.50. The correlation between the pubertal status at ages 11 and 16 was lower (*r*=0.31) as expected with the 5-year age gap.

**Figure 2:**
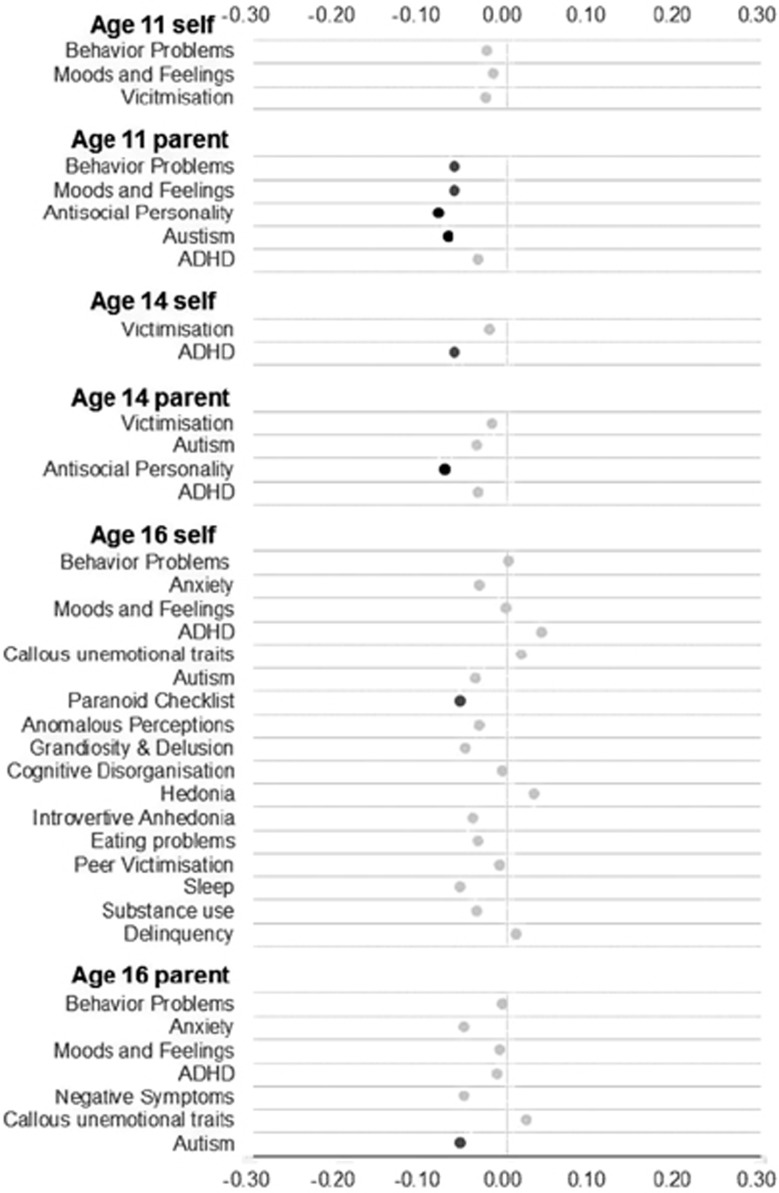
Correlations between age of menstruation and psychiatric and behavioral problems. Black dots indicate correlations significant at the 0.01, dark gray dots indicate correlations significant at the 0.05 level and light gray dots indicate non-significant correlations. ADHD, attention-deficit hyperactivity disorder.

We also looked at age of first menstruation and its associations with psychiatric and behavioral problems (Figure 2). In line with the literature, age of first menstruation was negatively correlated with the outcomes; however, only seven associations were significant and all the coefficients were below 0.10, with the largest effect seen with parent-reported antisocial personality disorder at age 11 (*r*=−0.08). These results again suggest that pubertal status explains <1% of the variance in psychiatric and behavioral outcomes.

To investigate nonlinear effects and to test the maturational deviance hypothesis, we conducted a series of regressions entering linear, quadratic (*x*^2^) and cubic (*x*^3^) terms hierarchically into the model and comparing the incremental F-change and associated significance. Supplementary Table S3 displays the polynomials where the quadratic or cubic model fitted the data better than the linear model (as seen from the *F*-change). Of the 92 comparisons for girls, there were nine significant nonlinear relationships, of which five were quadratic and four were cubic. Nonetheless, all but two associations remained below 0.10, indicating again that puberty generally explains less than 1% of the variance in these outcome measures even when nonlinearity is taken into account. For boys, out of the 92 comparisons, there were 11 nonlinear associations, of which nine were quadratic. The largest nonlinear association was between age 16 pubertal development and age 16 callous unemotional traits (*r*=0.24) in which a quadratic relationship was a significant improvement in fit from the linear model (*F*-change=12.63). See Supplementary Tables S4.1–S4.6 for all the nonlinear analyses at each age for boys and girls separately. When replicating these analyses with co-twins, only two of the nonlinear associations remained significant: (age 11 pubertal development and age 14 parent-report autism for both girls and boys). Therefore, these results indicate a mainly linear association for all of the outcome measures.

In summary, our analyses of linearity do not support the categorization of pubertal development into early and late groups. Nonetheless, to compare our results with those previously reported in the literature for group differences, ANOVA and contrasts were used to look at the effects of early and late relative pubertal development on adverse life outcomes at three ages (Supplementary Tables S5.1–S5.7). Cohen's *d* ranged from −0.08 to −0.64 with the pattern of results similar to the correlational analyses. In line with our previous analysis, the highest association for girls emerged for pubertal development at age 11 and eating disorder at 16, with the on-time maturing girls more likely to have eating problems than those with late pubertal development. For boys, again similar to our correlational analyses, the largest effect was seen between relative pubertal development and anomalous perceptions at age 11, with the early puberty group scoring higher on this measure compared to those with on-time pubertal development.

Finally, we compared our results to those found by Graber and colleagues,^4^ who categorized pubertal development and looked at the relationship between early, on time and late pubertal development as assessed by the PDS and a selection of psychosocial measures at age 16. For the 24 measures that used analysis of variance, comparisons were reported for early vs on time and late vs on time for girls and for boys. Of these 96 comparisons, only 33% were significantly different. Although Graber *et al.* did not report effect sizes in their paper, we computed Cohen's *d* values using means and approximating s.d.'s to be 1.0 because standardized scores were reported. Cohen's *d* estimates were comparable to our own, representing small effect sizes and ranging from 0.11 to 0.40, with an average *d* of 0.25 for the 32 significant effects (see Supplementary Table S6 for effect sizes).

## Discussion

This study is the most comprehensive and systematic investigation to date of the effects of pubertal development on adverse outcomes both concurrently and over a 5-year period for both boys and girls separately and using diverse data collected from multiple raters in a large representative sample. We show that if an individual differences approach is taken, pubertal development has only a very small effect on the adverse outcomes, explaining <1% of the variance on average across the three ages.

In line with the literature, we found a larger effect of pubertal development on eating disorder; however, the effect size was still small with age 11 pubertal development explaining only 4% of the variance in eating disorder at age 16 for girls. Although the co-twins do not represent a completely independent sample, we also re-ran our analyses on the ‘other' member of each twin pair and the results were similar. We found that the association between age 11 pubertal development and eating disorder at 16 did not hold up to replication (Supplementary Table S2.1). It should be noted that our sample size of ~5000 for most analyses provides 80% power (*P*=0.05) to detect a correlation of 0.05.^30^

For both males and females, many of the modest associations were observed longitudinally, with pubertal development associated with later outcomes. As mentioned in the introduction, various hypotheses have been proposed to explain why early puberty may have an impact on later outcomes. One of these is the selective persistence hypothesis^9^ that suggests that the negative effects of early puberty may self-perpetuate for some traits, for example, the association between early pubertal development on depression.^31^ Early maturers may feel isolated from their less-developed peers at a time when forging friendships is important and this may persist into adolescence. This can be observed in the current sample for moods and feelings (tapping into depression), which is related to pubertal development at age 11 as well as age 16 for both boys and girls. However, it should be emphasized that associations between pubertal development and these outcomes are weak concurrently and across age.

Rather than using individual differences to investigate the relationship between pubertal development and adverse outcomes, most research has compared mean differences between groups by dividing the sample into early, normal and late pubertal development groups or the outcome variable into cases and controls. Although categorizing measures for odds ratio interpretation has its benefits for clinical application, this approach loses information and assumes nonlinearity. We suggest that the individual differences approach is more parsimonious, does not lose information, can test for linear and nonlinear effects, and focuses on effect size. We showed that relationships between pubertal development and adverse outcomes were mainly linear and of small effect size. We also showed using nonlinear models that the extremes of the distributions do not drive the relationship between pubertal development and adverse outcomes. There are only a few exceptions to linearity such as the relationship between pubertal development and self-reported callous–unemotional traits at 16, in which the quadratic relationship explained an additional 4% of the variance compared to the linear model for boys; however, this did not replicate when we conducted the analysis on the co-twin. The only replicable nonlinear results were for self-reported pubertal development at age 11 and parent-reported autism at age 14 for both girls and boys; however, this nonlinear association explained <1% of the variance.

The non-replication of both the correlational analysis and the nonlinear associations highlights the importance of taking significance as well as effect size into account, especially with large samples. Large samples such as ours mean that even very small effects can become significant. However, replication in an independent sample is the best way to test whether effects are true or merely false positives. Our sample of co-twins is not independent. If we had found replication, the lack of independence might have contributed to replicated false-positive findings. However, the lack of independence worked against finding so little replication. This suggests that the effects of pubertal development on behavioral and psychiatric traits should be interpreted with caution.

When looking at pubertal development, an advantage of an individual differences approach is that variables do not need to be split into groups, which involves some arbitrariness in addition to loss of information. When we split our pubertal development variable into early, average and late relative pubertal development and observe the mean differences with adverse outcomes, we see a pattern of results similar to those from our correlational analysis. However, with samples as large as in the present study, any differences between groups appear to be statistically significant and such null-hypothesis-significance testing does not focus on the issue of effect size, which is of paramount importance for interpreting the real-world significance of findings.

Indeed, when we calculated effect sizes for the results reported by Graber and colleagues^4^ and compared them to our results, we found that their effect sizes were small in magnitude, similar to ours. For example, they found that the effect of perceived early pubertal development on current feelings of depression was *d*=0.21 for boys, which is the same effect size as in our study. Although not all measures were directly comparable to our own, the results of both studies show that effect sizes were of the same magnitude for a variety of behavioral and psychiatric measures, as well as social traits such as parental support and conflict. However, it is worth noting that the study by Graber and colleagues used a measure of perceived pubertal development (‘Was your physical growth and development early, on time or late compared to most teenagers of your age?'), whereas the current study used a more objective measure of relative pubertal development by taking those who score ±1 s.d. from the mean. Some argue that perceived pubertal development may be more important than actual pubertal development for psychosocial adjustment and problem behaviors;^32, 33^ nevertheless, as the effect sizes were in the same magnitude, we can infer that both pubertal development relative to peers and perceived pubertal development have a small effect on behavioral and psychiatric problems in the general population.

Although the present study is the most thorough and most powerful analysis of associations between pubertal development and adverse outcomes, there are several limitations to consider. First, the participants were twins and twins might differ in pubertal development from non-twins, although there are no data to support this hypothesis. Second, as in many studies of pubertal development, our measures relied on self-reports. Although these measures of physical development have been shown to be valid,^27, 28^ more objective measures would be desirable. For example, although the relationship between pubertal development and eating disorders was modest in the present study, looking at hormonal changes during puberty and their links to eating disorder symptoms has been highlighted as a potential avenue of research.^34^ Indeed, using direct rather than secondary sex characteristics as a way to measure pubertal development might help to elucidate links between pubertal development and adverse outcomes.

Another limitation is that data were available only through age 16. Although we found only small effects of pubertal development on adverse outcomes through age 16, it has been suggested that these effects might increase and combine with other risk factors to have larger cumulative effects in early adulthood.^35^ TEDS is currently collecting data in emerging adulthood, so that we will be able to study longer-term effects in the future. Finally, it is worth noting that, although we found small effects of pubertal development in the current sample, it may have a larger impact in other samples, such as clinical samples.^36, 37^

Here, for we believe the first time, we systematically analyzed data collected longitudinally from a large representative sample to investigate associations between pubertal development, and psychiatric and behavioral outcomes. We conclude that, although puberty is a time of rapid physical and emotional growth, pubertal development explains little variance in adverse outcomes as the small effect sizes indicate that associations are weak and should not warrant major concern, at least in non-clinical samples.

## Figures and Tables

**Table 1:** Sample size, means (and s.d.'s) of study variables by gender

	N	*Whole sample*	*Boys*	*Girls*	F	R^2^
*Age 11*						
Self-report						
Behavior problems	5057	8.79 (5.30)	9.37 (5.36)	8.28 (5.20)	53.62**^a^	0.01
Moods and feelings	5077	3.29 (3.85)	3.43 (3.73)	3.16 (3.95)	6.11*	<0.01
Victimisation	5623	7.80 (7.39)	8.68 (7.83)	7.01 (6.87)	69.66** a	0.01
Pubertal development	5066	1.72 (0.58)	1.54 (0.47)	1.88 (0.62)	536.44**^a^	0.09
Parent report						
Behavior problems	5056	6.82 (4.92)	7.51 (5.06)	6.22 (4.67)	86.52**^a^	0.02
Moods and feelings	5065	1.73 (2.75)	1.83 (2.67)	1.65 (2.82)	5.54*	<0.01
Antisocial personality	5078	7.12 (4.32)	7.93 (4.51)	6.43 (4.02)	154.04**^a^	0.03
Autism	5245	4.69 (3.15)	5.22 (3.31)	4.23 (2.95)	129.61**^a^	0.02
ADHD	5070	13.27 (7.80)	11.13 (8.73)	7.87 (7.19)	206.84**^a^	0.04
						
*Age 14*						
Self-report						
Victimisation	3173	7.64 (7.21)	8.51 (7.78)	6.93 (6.63)	41.93**^a^	0.01
ADHD	3054	13.28 (7.80)	14.00 (8.33)	12.68 (7.67)	21.15**	<0.01
Pubertal development	2905	2.75 (0.61)	2.46 (0.57)	3.00 (0.53)	815.16**^a^	0.22
Parent report						
Victimisation	2845	6.50 (7.65)	6.81 (7.99)	6.25 (7.34)	4.49*^a^	<0.01
Autism	3039	36.94 (21.18)	38.31 (12.74)	35.75 (11.55)	33.84**^a^	0.01
Antisocial personality	3065	7.78 (5.05)	8.67 (5.22)	76.01 (4.78)	84.53**^a^	0.03
ADHD	3087	8.28 (7.96)	9.95 (8.77)	6.83 (6.85)	121.10**^a^	0.04
						
*Age 16*						
Self-report						
Behavior problems	4815	9.49 (5.12)	8.89 (4.93)	9.96 (5.22)	53.36**^a^	0.01
Anxiety	4820	7.97 (5.87)	6.10 (4.75)	9.49 (6.22)	481.33**^a^	0.09
Moods and feelings	4821	3.60 (4.42)	2.64 (3.49)	4.38 (4.91)	202.78**^a^	0.04
ADHD	1073	4.74 (0.86)	4.72 (0.85)	4.76 (0.87)	0.60	<0.01
Callous unemotional traits	1070	19.61 (7.58)	22.30 (7.88)	17.96 6.91)	80.93**^a^	0.07
Autism	4818	11.88 (5.77)	12.19 (5.70)	11.63 (5.81)	11.20**	<0.01
Paranoid checklist	4813	12.17 (10.63)	11.76 (10.42)	12.50 (10.78)	5.73*	<0.01
Anomalous perceptions	4821	4.66 (6.01)	4.28 (5.73)	4.97 (6.21)	16.07**^a^	<0.01
Grandiosity and delusion	4817	5.32 (4.43)	5.84 (4.59)	4.91 (4.25)	53.26**^a^	0.01
Cognitive disorganization	4815	3.96 (2.85)	3.40 (2.72)	4.41 (2.87)	155.21**^a^	0.03
Hedonia	4817	33.68 (7.93)	31.52 (7.98)	35.42 (7.44)	299.96**^a^	0.06
Introvertive anhedonia	4814	1.30 (1.32)	1.47 (1.31)	1.16 (1.31)	61.13**^a^	0.01
Eating problems	1067	3.18 (1.96)	2.14 (1.40)	3.84 (1.97)	272.03**^a^	0.18
Peer victimisation	2502	3.50 (3.22)	4.09 (2.23)	3.06 (3.14)	63.92**	0.03
Sleep	3747	7.87 (4.72)	7.25 (4.33)	8.41 (4.97)	57.63**^a^	0.02
Substance use	3747	2.58 (2.73)	2.62 (2.82)	2.55 (2.66)	0.96	<0.01
Delinquency	1074	5.34 (7.24)	6.50 (7.87)	4.61 (6.72)	16.50**^a^	0.02
Pubertal development	2234	3.36 (0.42)	3.2 (0.41)	3.49 (0.37)	385.73**	0.15
Parent report						
Behavior problems	4836	3.80 (3.18)	4.28 (3.36)	3.41 (2.98)	86.01**^a^	0.02
Anxiety	4837	3.56 (4.20)	2.71 (3.57)	4.24 (4.53)	176.97**^a^	0.03
Moods and feelings	4834	0.97 (2.27)	0.74 (1.83)	1.16 (2.56)	43.33**^a^	<0.01
ADHD	4832	6.73 (7.45)	8.05 (8.25)	5.66 (6.55)	116.51**^a^	0.03
Negative symptoms	4832	2.81 (3.87)	3.17 (4.08)	2.51 (3.67)	33.45**^a^	<0.01
Callous unemotional traits	4835	17.55 (9.16)	19.74 (9.39)	15.77 (8.57)	229.85**^a^	0.05
Autism	4835	24.05 (10.63)	25.97 (10.98)	22.49 (10.07)	126.94***^a^	0.03
Self-report						
Age of menarche	1864	12.75 (1.22)				

Abbreviations: ANOVA, analysis of variance; ADHD, attention-deficit hyperactivity disorder.

Note: means and s.d.'s calculated with raw data with one twin randomly selected from each twin pair. S.d.'s are shown in parentheses. *N*=sample size after exclusions. ANOVA performed on age-regressed data from one randomly selected twin per pair to test the effect of sex. When the Levene's test of homogeneity of variance was violated, the non-parametric Welch test was used instead. Results=*F* statistic; *P<0.05; **P<0.01; *R*^2^=proportion of variance explained by sex.

a Homogeneity of variance was not equal between sexes; Welch test used instead.
