# Supplementary material for: Weak associations between pubertal development and psychiatric and behavioral problems
Source: Transl Psychiatry. 2017 Apr 18;7(4):e1098–. doi: 10.1038/tp.2017.63 (PMC5416703; doi:10.1038/tp.2017.63)
Supplement: Supplementary Information [file tp201763x1.pdf]

**Supplementary Material**

# Weak associations between pubertal development and psychiatric and behavioral problems.

Emily Smith-Woolley, MSc[1]*, Kaili Rimfeld, MSc[1]*, Robert Plomin, PhD[1].

*Joint first authors*

**Abstract:**
Pubertal development has been associated with adverse outcomes throughout adolescence and adulthood. However, much of the previous literature has categorized outcome variables and pubertal timing measures for ease of mean difference or odds-ratio interpretation. We use a UK-representative sample of over 5 000 individuals drawn from the Twins Early Development Study to extend this literature by adopting an individual differences approach and emphasizing effect sizes. We investigate a variety of psychiatric and behavioral measures collected longitudinally at ages 11, 14 and 16, for multiple raters and for males and females separately. In addition, we use two measures of pubertal development: totals on the Pubertal Development Scale at each age, as well as age of menarche for girls. We found that pubertal development, however assessed, was linearly associated with a range of psychiatric and behavioral outcomes; however, the effect sizes of these associations were modest for both males and females with most correlations between -.10 and .10. Our systematic analysis of associations between pubertal development and psychiatric and behavioral problems is the most comprehensive to date. The results showing linearity of the effects of pubertal development support an individual differences approach, treating both pubertal development and associated outcomes as continuous rather than categorical variables. We conclude that pubertal development explains little variance in psychiatric and behavioral outcomes (less than 1% on average). The small effect sizes indicate that the associations are weak and should not warrant major concern at least in non-clinical populations.

**Tables:**


Table S1 - Missingness at ages 11, 14 and 16 and variance explained in psychiatric, behavioural and pubertal measures

Table S2.1 – Correlations between pubertal development at 11 and psychiatric and behavioral problems concurrently and over time for girls.

Table S2.2 – Correlations between pubertal development at 14 and psychiatric and behavioral problems concurrently and over time for girls.

Table S2.3 – Correlations between pubertal development at 16 and psychiatric and behavioral problems concurrently for girls.

Table S2.4 – Correlations between age of menarche and psychiatric and behavioral problems over time for girls.

Table S2.5 – Correlations between pubertal development at 11 and psychiatric and behavioral problems concurrently and over time for boys.

Table S2.6 – Correlations between pubertal development at 14 and psychiatric and behavioral problems concurrently and over time for boys.

Table S2.7 – Correlations between pubertal development at 16 and psychiatric and behavioral problems concurrently for boys.

Table S3 – Significant non-linear results at ages 11, 14 and 16 for males and females separately

Table S4.1 – Quadratic and cubic polynomials between girls' age 11 PDS score and behavioral and psychiatric problems concurrently and over time

Table S4.2 – Quadratic and cubic polynomials between boys' age 11 PDS score and behavioral and psychiatric problems concurrently and over time

Table S4.3 – Quadratic and cubic polynomials between girls' age 14 PDS score and behavioral and psychiatric problems concurrently and over time

Table S4.4 – Quadratic and cubic polynomials between boys' age 14 PDS score and behavioral and psychiatric problems concurrently and over time

Table S4.5 – Quadratic and cubic polynomials between girls' age 16 PDS score and behavioral and psychiatric problems concurrently and over time

Table S4.6 – Quadratic and cubic polynomials between boys' age 16 PDS score and behavioral and psychiatric problems concurrently and over time

Table S5.1 – Analysis of variance and associated post-hoc comparisons looking at early, on-time and late pubertal timing in girls at age 11 and behavioral and psychiatric problems concurrently and over time

Table S5.2 – Analysis of variance and associated post-hoc comparisons looking at early, on-time and late pubertal timing in boys at age 11 and behavioral and psychiatric problems concurrently and over time

Table S5.3 – Analysis of variance and associated post-hoc comparisons looking at early, on-time and late pubertal timing in girls at age 14 and behavioral and psychiatric problems concurrently and at age 16.

Table S5.4 – Analysis of variance and associated post-hoc comparisons looking at early, on-time and late pubertal timing in boys at age 14 and behavioral and psychiatric problems concurrently and at age 16.

Table S5.5 – Analysis of variance and associated post-hoc comparisons looking at early, on-time and late pubertal timing in girls at age 16 and behavioral and psychiatric problems concurrently at age 16.

Table S5.6 – Analysis of variance and associated post-hoc comparisons looking at early, on-time and late pubertal timing in boys at age 16 and behavioral and psychiatric problems concurrently at age 16.

Table S5.7 – Analysis of variance and associated post-hoc comparisons looking at early, on-time and late menarcheal timing in girls and behavioral and psychiatric problems concurrently and at age 14 and 16.

*Table S6* – Estimated effect sizes for the contrasts between early vs on-time and late vs. on-time pubertal development from Graber et al 1997

**Figures:**

Figure S1.1 – Correlations between girls PDS score at age 14 and psychiatric and behavioral problems. Black dots indicate correlations significant at the .01, dark grey dots indicate correlations significant at the .05 level and light grey dots indicate non-significant correlations.

Figure S1.2 – Correlations between boys PDS score at age 14 and psychiatric and behavioral problems. Black dots indicate correlations significant at the .01, dark grey dots indicate correlations significant at the .05 level and light grey dots indicate non-significant correlations.

Figure S1.3 – Correlations between girls PDS score at age 16 and psychiatric and behavioral problems. Black dots indicate correlations significant at the .01, dark grey dots indicate correlations significant at the .05 level and light grey dots indicate non-significant correlations.

Figure S1.4 – Correlations between boys PDS score at age 16 and psychiatric and behavioral problems. Black dots indicate correlations significant at the .01, dark grey dots indicate correlations significant at the .05 level and light grey dots indicate non-significant correlations

***Table S1*** - Missingness at ages 11, 14 and 16 and variance explained in psychiatric, behavioural and pubertal measures

| | | | F | $R^2$ |
|---|---|---|---|---|
| Age 11 Missingness | Self-report age 14 | Victimisation | 3.08 | <.01 |
| | | ADHD | 11.36** | <.01 |
| | | Puberty | 3.56 | <.01 |
| | Parent-report age 14 | Victimisation | 9.12** | <.01 |
| | | Autism | 18.27** | <.01 |
| | | Antisocial Personality | 23.03** | <.01 |
| | | ADHD | 28.74** | <.01 |
| | Self-report age 16 | Behavior Problems | 6.25* | <.01 |
| | | Anxiety | 2.96 | <.01 |
| | | Moods and Feelings | 0.50 | <.01 |
| | | ADHD | 0.75 | <.01 |
| | | Callous unemotional traits | 8.02** | <.01 |
| | | Autism | 8.87** | <.01 |
| | | Paranoid Checklist | 1.99 | <.01 |
| | | Anomalous Perceptions | 0.28 | <.01 |
| | | Grandiosity & Delusion | 0.98 | <.01 |
| | | Cognitive Disorganisation | 6.43* | <.01 |
| | | Hedonia | 0.01 | <.01 |
| | | Introvertive Anhedonia | 14.51** | <.01 |
| | | Eating problems | 0.09 | <.01 |
| | | Peer Victimisation | 0.02 | <.01 |
| | | Sleep | 1.2 | <.01 |
| | | Substance use | 3.89* | <.01 |
| | | Delinquency | 0.85 | <.01 |
| | | Puberty | 1.56 | <.01 |
| | Parent-report age 16 | Behavior Problems | 20.73** | <.01 |
| | | Anxiety | 31.43** | <.01 |
| | | Moods and Feelings | 2.82 | <.01 |
| | | ADHD | 57.93** | <.01 |
| | | Negative Symptoms | 17.89** | <.01 |
| | | Callous unemotional traits | 0.49 | <.01 |
| | | Autism | 41.08** | <.01 |
| Age 14 missingness | Self-report age 11 | Behavior Problems | 9.95** | <.01 |
| | | Moods and Feelings | 0.5 | <.01 |
| | | Victimisation | 17.32** | <.01 |
| | | Puberty | 3.43 | <.01 |
| | Parent-report age 11 | Behavior Problems | 38.01** | <.01 |
| | | Moods and Feelings | 11.14** | <.01 |
| | | Antisocial Personality | 44.31** | <.01 |
| | | Autism | 25.24** | <.01 |
| | | ADHD | 43.07** | <.01 |
| | Self-report age 16 | Behavior Problems | 13.95** | <.01 |
| | | Anxiety | 2.26 | <.01 |
| | | Moods and Feelings | 0.57 | <.01 |
| | | ADHD | 5.97* | <.01 |
| | | Callous unemotional traits | 2.51 | <.01 |
| | | Autism | 1.55 | <.01 |
| | | Paranoid Checklist | 3.23 | <.01 |

| | | | | |
|---|---|---|---|---|
| | | Anomalous Perceptions | 0.23 | <.01 |
| | | Grandiosity & Delusion | 3.1 | <.01 |
| | | Cognitive Disorganisation | 6.30* | <.01 |
| | | Hedonia | 0.01 | <.01 |
| | | Introvertive Anhedonia | 32.28** | <.01 |
| | | Eating problems | 1.07 | <.01 |
| | | Peer Victimisation | 1.05 | <.01 |
| | | Sleep | 0.01 | <.01 |
| | | Substance use | 4.26* | <.01 |
| | | Delinquency | 1.19 | <.01 |
| | | Puberty | 6.96** | <.01 |
| | Parent-report age 16 | Behavior Problems | 76.88** | <.01 |
| | | Anxiety | 25.49** | <.01 |
| | | Moods and Feelings | 3.32 | <.01 |
| | | ADHD | 95.95** | <.01 |
| | | Negative Symptoms | 42.00** | <.01 |
| | | Callous unemotional traits | 29.42** | <.01 |
| | | Autism | 40.66** | <.01 |
| Age 16 missingness | Self-report age 11 | Victimisation | 90.46 | <.01 |

Note; $R^2$= proportion of variance explained by missingness at each age; ** = *p*<.01; * = *p*<.05. There was only one measure at age 11 and 14 (self-report victimisation at age 11) for which there was missing data at 16 but data present at age 11 or 14, therefore the rest of the scores could not be computed.

***Table S2.1*** - Correlations between pubertal development at 11 and psychiatric and behavioral problems concurrently and over time for girls. Analysis was conducted by randomly selecting 1 twin per pair and replicated on the co-twin.

| | | | Twin 1 correlation | Twin 1 $R^2$ | Twin 2 correlation | Twin 2 $R^2$ |
|---|---|---|---|---|---|---|
| Age 11 | Self-report | Behavior Problems | .07** | .01 | .08** | .01 |
| | | Moods and Feelings | .08** | .01 | .09** | .01 |
| | | Victimisation | .07** | .01 | .07** | .01 |
| | Parent-report | Behavior Problems | .05** | <.01 | .04 | <.01 |
| | | Moods and Feelings | .05** | <.01 | .02 | <.01 |
| | | Antisocial Personality | .04* | <.01 | .05** | <.01 |
| | | Autism | .04* | <.01 | .04* | <.01 |
| | | ADHD | .02 | <.01 | .03 | <.01 |
| Age 14 | Self-report | Victimisation | .11** | .01 | .08** | .01 |
| | | ADHD | .05 | <.01 | .03 | <.01 |
| | Parent-report | Victimisation | .10** | .01 | .05 | <.01 |
| | | Autism | -.02 | <.01 | -.04 | <.01 |
| | | Antisocial Personality | .04 | <.01 | -.01 | <.01 |
| | | ADHD | .01 | <.01 | -.02 | <.01 |
| Age 16 | Self-report | Behavior Problems | .05* | <.01 | .05* | <.01 |
| | | Anxiety | .05* | <.01 | .07** | <.01 |
| | | Moods and Feelings | .07** | <.01 | .07** | <.01 |
| | | ADHD | .01 | <.01 | .07 | <.01 |
| | | Callous unemotional traits | .03 | <.01 | -.05 | <.01 |
| | | Autism | .04 | <.01 | .05* | <.01 |
| | | Paranoid Checklist | .12** | .02 | .06** | <.01 |
| | | Anomalous Perceptions | .10** | .01 | .06** | <.01 |
| | | Grandiosity & Delusion | .08** | .01 | .05* | <.01 |
| | | Cognitive Disorganisation | .04 | <.01 | .05* | <.01 |
| | | Hedonia | -.01 | <.01 | .03 | <.01 |
| | | Introvertive Anhedonia | <.01 | <.01 | .01 | <.01 |
| | | Eating problems | .21** | .04 | .03 | <.01 |
| | | Peer Victimisation | .08** | .01 | .02 | .01 |
| | | Sleep | .08** | .01 | .06** | <.01 |
| | | Substance use | .07** | .01 | .04 | <.01 |
| | | Delinquency | .07 | <.01 | .02 | <.01 |
| | Parent-report | Behavior Problems | .02 | <.01 | -.01 | <.01 |
| | | Anxiety | .03 | <.01 | -.00 | <.01 |
| | | Moods and Feelings | .04 | <.01 | .01 | <.01 |
| | | ADHD | -.01 | <.01 | -.00 | <.01 |
| | | Negative Symptoms | .01 | <.01 | -.03 | <.01 |
| | | Callous unemotional traits | -.03 | <.01 | -.03 | <.01 |
| | | Autism | -.01 | <.01 | -.02 | <.01 |

Note; $R^2$= proportion of variance explained by PDS; ** = $p<.01$; * = $p<.05$

***Table S2.2*** - Correlations between pubertal development at 14 and psychiatric and behavioral problems concurrently and over time for girls. Analysis was conducted by randomly selecting one twin per pair.

| | | | Correlation | $R^2$ |
|---|---|---|---|---|
| Age 14 | Self-report | Victimisation | .00 | .00 |
| | | ADHD | .03 | <.01 |
| | Parent-report | Victimisation | -.03 | <.01 |
| | | Autism | -.04 | <.01 |
| | | Antisocial Personality | .03 | <.01 |
| | | ADHD | -.01 | <.01 |
| Age 16 | Self-report | Behavior Problems | .02 | <.01 |
| | | Anxiety | .04 | <.01 |
| | | Moods and Feelings | .05 | <.01 |
| | | ADHD | -.06 | <.01 |
| | | Callous unemotional traits | .05 | <.01 |
| | | Autism | -.01 | <.01 |
| | | Paranoid Checklist | .07** | .01 |
| | | Anomalous Perceptions | .05* | <.01 |
| | | Grandiosity & Delusion | .06* | <.01 |
| | | Cognitive Disorganisation | .01 | <.01 |
| | | Hedonia | -.02 | <.01 |
| | | Introvertive Anhedonia | .01 | <.01 |
| | | Eating problems | .11* | .01 |
| | | Peer Victimisation | .03 | <.01 |
| | | Sleep | .03 | <.01 |
| | | Substance use | .06 | <.01 |
| | | Delinquency | .08 | .01 |
| | Parent-report | Behavior Problems | -.01 | <.01 |
| | | Anxiety | -.04 | <.01 |
| | | Moods and Feelings | .04 | <.01 |
| | | ADHD | -.01 | <.01 |
| | | Negative Symptoms | -.02 | <.01 |
| | | Callous unemotional traits | -.04 | <.01 |
| | | Autism | -.02 | <.01 |

Note; $R^2$= proportion of variance explained by PDS; ** = $p$<.01; * = $p$<.05

***Table S2.3*** - Correlations between pubertal development at 16 and psychiatric and behavioral problems concurrently for girls. Analysis was conducted by randomly selecting one twin per pair.

| | | | **Correlation** | **$R^2$** |
|---|---|---|---|---|
| Age 16 | Self-report | Behavior Problems | -.03 | <.01 |
| | | Anxiety | -.04 | <.01 |
| | | Moods and Feelings | .04 | <.01 |
| | | ADHD | -.05 | <.01 |
| | | Callous unemotional traits | -.03 | <.01 |
| | | Autism | <.01 | <.01 |
| | | Paranoid Checklist | .02 | <.01 |
| | | Anomalous Perceptions | -.04 | <.01 |
| | | Grandiosity & Delusion | -.02 | <.01 |
| | | Cognitive Disorganisation | -.04 | <.01 |
| | | Hedonia | -.02 | <.01 |
| | | Introvertive Anhedonia | -.07* | .01 |
| | | Eating problems | .07 | .01 |
| | | Peer Victimisation | -.03 | <.01 |
| | | Sleep | -.03 | <.01 |
| | | Substance use | .01 | <.01 |
| | | Delinquency | <.01 | <.01 |
| | Parent-report | Behavior Problems | -.03 | <.01 |
| | | Anxiety | -.03 | <.01 |
| | | Moods and Feelings | .01 | <.01 |
| | | ADHD | -.05 | <.01 |
| | | Negative Symptoms | <.01 | <.01 |
| | | Callous unemotional traits | -.07* | <.01 |
| | | Autism | -.01 | <.01 |

Note; $R^2$= proportion of variance explained by PDS; ** = $p$<.01; * = $p$<.05

***Table S2.4*** - Correlations between age of menarche and psychiatric and behavioral problems over time for girls. Analyses were conducted by randomly selecting one twin per pair and replicated on the co-twin.

| | | | Twin 1 correlation | Twin 1 $R^2$ | Twin 2 correlation | Twin 2 $R^2$ |
|---|---|---|---|---|---|---|
| Age 11 | Self-report | Behavior Problems | -.02 | <.01 | -.03 | <.01 |
| | | Moods and Feelings | -.02 | <.01 | -.02 | <.01 |
| | | Victimisation | -.03 | <.01 | .01 | <.01 |
| | Parent-report | Behavior Problems | -.06* | <.01 | .02 | <.01 |
| | | Moods and Feelings | -.06* | <.01 | .00 | <.01 |
| | | Antisocial Personality | -.08** | .01 | -.06* | <.01 |
| | | Autism | -.07** | .01 | -.03 | <.01 |
| | | ADHD | -.03 | <.01 | .04 | <.01 |
| Age 14 | Self-report | Victimisation | -.02 | <.01 | -.00 | <.01 |
| | | ADHD | -.06* | <.01 | -.06* | <.01 |
| | Parent-report | Victimisation | -.02 | <.01 | -.04 | <.01 |
| | | Autism | -.04 | <.01 | -.02 | <.01 |
| | | Antisocial Personality | -.07** | .01 | -.06* | <.01 |
| | | ADHD | -.03 | <.01 | -.02 | <.01 |
| Age 16 | Self-report | Behavior Problems | .00 | <.01 | -.06* | <.01 |
| | | Anxiety | -.03 | <.01 | -.04 | <.01 |
| | | Moods and Feelings | <.01 | <.01 | -03 | <.01 |
| | | ADHD | .04 | <.01 | .01 | <.01 |
| | | Callous unemotional traits | .02 | <.01 | -.02 | <.01 |
| | | Autism | -.02 | <.01 | -.08** | .01 |
| | | Paranoid Checklist | -.05* | <.01 | -.05 | <.01 |
| | | Anomalous Perceptions | -.03 | <.01 | -.06* | <.01 |
| | | Grandiosity & Delusion | -.05 | <.01 | -.02 | <.01 |
| | | Cognitive Disorganisation | -.01 | <.01 | -.06* | <.01 |
| | | Hedonia | .03 | <.01 | .03 | <.01 |
| | | Introvertive Anhedonia | -.04 | <.01 | -.06* | <.01 |
| | | Eating problems | -.03 | <.01 | -.05 | <.01 |
| | | Peer Victimisation | -.01 | <.01 | -.03 | <.01 |
| | | Sleep | -.05 | <.01 | -.03 | <.01 |
| | | Substance use | -.03 | <.01 | -.05 | <.01 |
| | | Delinquency | .01 | <.01 | -.02 | <.01 |
| | Parent-report | Behavior Problems | -.01 | <.01 | -.03 | <.01 |
| | | Anxiety | -.05 | <.01 | -.05 | <.01 |
| | | Moods and Feelings | -.01 | <.01 | -.03 | <.01 |
| | | ADHD | -.01 | <.01 | -.00 | <.01 |
| | | Negative Symptoms | -.05 | <.01 | .02 | <.01 |
| | | Callous unemotional traits | .02 | <.01 | .01 | <.01 |
| | | Autism | -.05* | <.01 | -.04 | <.01 |

Note; $R^2$= proportion of variance explained by PDS; ** = $p$<.01; * = $p$<.05

***Table S2.5*** - Correlations between pubertal development at 11 and psychiatric and behavioral problems concurrently and over time for boys. Analyses were conducted by randomly selecting one twin per pair and replicated on the co-twin.

| | | | Twin 1 correlation | Twin 1 $R^2$ | Twin 2 correlation | Twin 2 $R^2$ |
|---|---|---|---|---|---|---|
| Age 11 | Self-report | Behavior Problems | .12** | .02 | .13** | .02 |
| | | Moods and Feelings | .13** | .02 | .13** | .02 |
| | | Victimisation | .14** | .02 | .14** | .02 |
| | Parent-report | Behavior Problems | .03 | <.01 | .07** | <.01 |
| | | Moods and Feelings | .00 | <.01 | .03 | <.01 |
| | | Antisocial Personality | .05 | <.01 | .08** | .01 |
| | | Autism | .05* | <.01 | .07** | <.01 |
| | | ADHD | .04 | <.01 | .07** | <.01 |
| Age 14 | Self-report | Victimisation | .07* | <.01 | .09** | .01 |
| | | ADHD | .04 | <.01 | .08** | .01 |
| | Parent-report | Victimisation | .05 | <.01 | .09** | .01 |
| | | Autism | <.01 | <.01 | .04 | <.01 |
| | | Antisocial Personality | .05 | <.01 | .08** | .01 |
| | | ADHD | <.01 | <.01 | .06* | <.01 |
| Age 16 | Self-report | Behavior Problems | .07** | <.01 | .05* | <.01 |
| | | Anxiety | .08** | .01 | .05 | <.01 |
| | | Moods and Feelings | .11** | .01 | .05 | <.01 |
| | | ADHD | -.08 | .01 | -.05 | <.01 |
| | | Callous unemotional traits | .07 | .01 | .03 | <.01 |
| | | Autism | .02 | <.01 | -.03 | <.01 |
| | | Paranoid Checklist | .09** | .01 | .06* | <.01 |
| | | Anomalous Perceptions | .14** | .02 | .06* | <.01 |
| | | Grandiosity & Delusion | .05* | <.01 | .05 | <.01 |
| | | Cognitive Disorganisation | .06* | <.01 | .05 | <.01 |
| | | Hedonia | .04 | <.01 | .02 | <.01 |
| | | Introvertive Anhedonia | -.01 | <.01 | -.04 | <.01 |
| | | Eating problems | .07 | .01 | -.01 | <.01 |
| | | Peer Victimisation | .04 | <.01 | .06 | <.01 |
| | | Sleep | .11** | .01 | .08** | .01 |
| | | Substance use | .09** | .01 | .14** | .02 |
| | | Delinquency | .08 | .01 | .17** | .03 |
| | Parent-report | Behavior Problems | .01 | <.01 | .07** | <.01 |
| | | Anxiety | <.01 | <.01 | -.02 | <.01 |
| | | Moods and Feelings | .01 | <.01 | .01 | <.01 |
| | | ADHD | .03 | <.01 | .04 | <.01 |
| | | Negative Symptoms | .01 | <.01 | .00 | <.01 |
| | | Callous unemotional traits | .03 | <.01 | -.01 | <.01 |
| | | Autism | .01 | <.01 | -.02 | <.01 |

Note; $R^2$= proportion of variance explained by PDS; ** = $p$<.01; * = $p$<.05

***Table S2.6*** - Correlations between pubertal development at 14 and psychiatric and behavioral problems concurrently and over time for boys. Analysis was conducted by randomly selecting one twin per pair.

| | | | Correlation | $R^2$ |
|---|---|---|---|---|
| Age 14 | Self-report | Victimisation | -.05 | <.01 |
| | | ADHD | -.06* | <.01 |
| | Parent-report | Victimisation | -.05 | <.01 |
| | | Autism | -.08** | .01 |
| | | Antisocial Personality | -.01 | <.01 |
| | | ADHD | -.08** | <.01 |
| Age 16 | Self-report | Behavior Problems | .01 | <.01 |
| | | Anxiety | <.01 | <.01 |
| | | Moods and Feelings | .05 | <.01 |
| | | ADHD | .02 | <.01 |
| | | Callous unemotional traits | .05 | <.01 |
| | | Autism | <.01 | <.01 |
| | | Paranoid Checklist | .01 | <.01 |
| | | Anomalous Perceptions | .06 | <.01 |
| | | Grandiosity & Delusion | .01 | <.01 |
| | | Cognitive Disorganisation | .03 | <.01 |
| | | Hedonia | -.04 | <.01 |
| | | Introvertive Anhedonia | <.01 | <.01 |
| | | Eating problems | .04 | <.01 |
| | | Peer Victimisation | -.06 | <.01 |
| | | Sleep | .12** | .02 |
| | | Substance use | .04 | <.01 |
| | | Delinquency | .03 | <.01 |
| | Parent-report | Behavior Problems | -.07* | .01 |
| | | Anxiety | -.04 | <.01 |
| | | Moods and Feelings | -.01 | <.01 |
| | | ADHD | -.08** | .01 |
| | | Negative Symptoms | -.05 | <.01 |
| | | Callous unemotional traits | <.01 | <.01 |
| | | Autism | -.04 | <.01 |

Note; $R^2$= proportion of variance explained by PDS; ** = $p$<.01; * = $p$<.05

***Table S2.7*** - Correlations between pubertal development at 16 and psychiatric and behavioral problems concurrently for boys. Analysis was conducted by randomly selecting one twin per pair.

| | | | Correlation | $R^2$ |
|---|---|---|---|---|
| Age 16 | Self-report | Behavior Problems | -.07* | .01 |
| | | Anxiety | .05 | <.01 |
| | | Moods and Feelings | -.05 | <.01 |
| | | ADHD | <.01 | <.01 |
| | | Callous unemotional traits | -.07 | <.01 |
| | | Autism | -.06 | <.01 |
| | | Paranoid Checklist | -.01 | <.01 |
| | | Anomalous Perceptions | -.03 | <.01 |
| | | Grandiosity & Delusion | -.09* | .01 |
| | | Cognitive Disorganisation | -.01 | <.01 |
| | | Hedonia | .02 | <.01 |
| | | Introvertive Anhedonia | -.06 | <.01 |
| | | Eating problems | .04 | <.01 |
| | | Peer Victimisation | -.01 | <.01 |
| | | Sleep | .11* | .01 |
| | | Substance use | .01 | <.01 |
| | | Delinquency | -.05 | <.01 |
| | Parent-report | Behavior Problems | -.10** | .01 |
| | | Anxiety | -.08* | .01 |
| | | Moods and Feelings | -.05 | <.01 |
| | | ADHD | -.08* | .01 |
| | | Negative Symptoms | -.03 | <.01 |
| | | Callous unemotional traits | -.10** | .01 |
| | | Autism | -.08* | .01 |

Note; $R^2$= proportion of variance explained by PDS; ** = $p$<.01; * = $p$<.05

***Table S3*** - Significant non-linear results at ages 11, 14 and 16 for males and females separately. Analysis was conducted by randomly selecting one twin per pair.

| | | Model | *R* | $R^2$ | Adj $R^2$ | Std. Error | $R^2$ Change | *F* Change |
|---|---|---|---|---|---|---|---|---|
| **GIRLS** | | | | | | | | |
| Age 11 PDS score | Parent-report CAST at 11 | Linear | .04 | <.01 | <.01 | .86 | <.01 | 4.82* |
| | | Quadratic | .05 | <.01 | <.01 | .86 | <.01 | 2.65 |
| | | Cubic | .07 | <.01 | <.01 | .86 | <.01 | 6.77** |
| | Parent-report AQ at 14 | Linear | .02 | <.01 | <.01 | .90 | <.01 | 0.80 |
| | | Quadratic | .06 | <.01 | <.01 | .90 | <.01 | 4.75* |
| | | Cubic | .06 | <.01 | <.01 | .90 | <.01 | 0.03 |
| | Self-report ADHD at 16 | Linear | .01 | <.01 | <.01 | 1.04 | .00 | .09 |
| | | Quadratic | .10 | .01 | .01 | 1.03 | .01 | 5.17* |
| | | Cubic | .10 | <.01 | <.01 | 1.03 | .00 | .07 |
| | Self-report hedonia at 16 | Linear | .01 | <.01 | <.01 | .94 | .00 | .39 |
| | | Quadratic | .03 | <.01 | <.01 | .94 | .00 | 1.92 |
| | | Cubic | .06 | <.01 | <.01 | .94 | .00 | 4.41* |
| | Parent-report anxiety at 16 | Linear | .03 | <.01 | <.01 | 1.05 | .00 | 1.54 |
| | | Quadratic | .04 | <.01 | <.01 | 1.05 | .00 | 1.75 |
| | | Cubic | .06 | <.01 | <.01 | 1.05 | .00 | 4.69* |
| Age 14 PDS score | Parent-report AQ at 14 | Linear | .04 | <.01 | <.01 | .90 | <.01 | 2.07 |
| | | Quadratic | .08 | <.01 | <.01 | .90 | .01 | 6.95** |
| | | Cubic | .08 | <.01 | <.01 | .90 | <.01 | 0.08 |
| | Self-report Gradiosity at 16 | Linear | .06 | <.01 | <.01 | .92 | <.01 | 5.60* |
| | | Quadratic | .08 | <.01 | <.01 | .92 | <.01 | 2.08 |
| | | Cubic | .10 | .01 | .01 | .91 | .01 | 6.55* |
| | Self-report hedonia at 16 | Linear | .02 | <.01 | <.01 | .92 | <.01 | 0.51 |
| | | Quadratic | .07 | <.01 | <.01 | .92 | <.01 | 5.29* |
| | | Cubic | .07 | <.01 | <.01 | .92 | <.01 | 0.39 |
| | Parent-report AQ at 16 | Linear | .02 | <.01 | <.01 | .91 | <.01 | 0.56 |
| | | Quadratic | .06 | <.01 | <.01 | .91 | <.01 | 4.81* |
| | | Cubic | .06 | <.01 | <.01 | .91 | <.01 | 0.02 |

| | | Model | *R* | $R^2$ | Adj $R^2$ | Std. Error | $R^2$ Change | *F* Change |
|---|---|---|---|---|---|---|---|---|
| **BOYS** | | | | | | | | |
| Age 11 PDS score | Self-report Victimisation at 14 | Linear | .07 | <.01 | <.01 | 1.07 | <.01 | 5.53* |
| | | Quadratic | .09 | <.01 | <.01 | 1.07 | <.01 | 4.16* |
| | | Cubic | .09 | <.01 | <.01 | 1.07 | <.01 | .06 |
| | Parent-report AQ at 14 | Linear | <.01 | <.01 | <.01 | 1.01 | <.01 | <.01 |
| | | Quadratic | .07 | <.01 | <.01 | 1.01 | .01 | 6.33* |
| | | Cubic | .08 | <.01 | <.01 | 1.01 | <.01 | 1.55 |
| | Self-report MFQ at 16 | Linear | .11 | .01 | .01 | .79 | .01 | 19.58** |
| | | Quadratic | .11 | .01 | .01 | .79 | .00 | .87 |
| | | Cubic | .12 | .01 | .01 | .79 | .00 | 5.11* |
| | | Linear | .08 | <.01 | <.01 | .97 | .01 | 2.34 |

| | | | | | | | | |
|---|---|---|---|---|---|---|---|---|
| | Self-report SWAN at 16 | Quadratic | .15 | .02 | .02 | .96 | .02 | 5.41* |
| | | Cubic | .15 | .02 | .01 | .96 | <.01 | .13 |
| Age 14 PDS score | Self-report Victimisation at 14 | Linear | .05 | <.01 | <.01 | 1.08 | <.01 | 3.36 |
| | | Quadratic | .09 | <.01 | <.01 | 1.08 | .01 | 7.21** |
| | | Cubic | .10 | <.01 | <.01 | 1.08 | <.01 | 1.31 |
| | Parent-report Victimisation at 14 | Linear | .05 | <.01 | <.01 | 1.02 | <.01 | 2.52 |
| | | Quadratic | .08 | <.01 | <.01 | 1.02 | <.01 | 4.64* |
| | | Cubic | .08 | <.01 | <.01 | 1.02 | <.01 | .13 |
| | Self-report Introvertive Anhedonia at 16 | Linear | <.01 | <.01 | <.01 | .98 | <.01 | .01 |
| | | Quadratic | .06 | <.01 | <.01 | .98 | <.01 | 4.00* |
| | | Cubic | .07 | <.01 | <.01 | .98 | <.01 | .52 |
| Age 16 PDS score | Self-report ICUT at 16 | Linear | .11 | .01 | <.01 | .99 | <.01 | 3.31 |
| | | Quadratic | .24 | .06 | .01 | .99 | <.01 | 12.63** |
| | | Cubic | .25 | .06 | .02 | .99 | <.01 | 1.22 |
| | Self-report Gradiosity at 16 | Linear | .09 | .01 | .01 | 1.0 | .01 | 5.90* |
| | | Quadratic | .12 | .01 | .01 | 1.0 | <.01 | 4.61* |
| | | Cubic | .12 | .02 | .01 | 1.0 | <.01 | 0.18 |
| | Self-report hedonia at 16 | Linear | .02 | <.01 | <.01 | .99 | <.01 | .28 |
| | | Quadratic | .10 | .01 | .01 | .99 | .01 | 6.69** |
| | | Cubic | .11 | .01 | .01 | .99 | <.01 | 1.23 |
| | Parent-report Conners at 16 | Linear | .08 | .01 | .01 | .94 | .01 | 4.53 |
| | | Quadratic | .10 | .01 | .01 | .94 | <.01 | 3.14 |
| | | Cubic | .14 | .02 | .01 | .94 | .01 | 5.84* |

Note: $R^2$= proportion of variance explained; Adj $R^2$ = adjusted $R^2$ in new model. Highlighted grey lines indicate significant non-linear results. When these results were replicated with the co-twin, only self-reported age 11 pubertal development and age 14 parent-report autism remained significant for girls and boys.
*Significant at the .05 level
** Significant at the .01 level

***Table S4.1*** - Linear, quadratic and cubic associations between girls' age 11 PDS score and behavioral and psychiatric problems concurrently and over time. Analysis was conducted by randomly selecting one twin per pair.

| | | | Model | R | $R^2$ | Adj $R^2$ | Std. Error | Change statistics | | | |
|---|---|---|---|---|---|---|---|---|---|---|---|
| | | | | | | | | $R^2$ Change | F Change | df1 | df2 |
| Age 11 | Self | Behavior Problems | Linear | 0.07 | .01 | .00 | .96 | .00 | 12.83** | 1.00 | 2685.00 |
| | | | Quadratic | 0.07 | .01 | .00 | .96 | .00 | .00 | 1.00 | 2684.00 |
| | | | Cubic | 0.08 | .01 | .01 | .96 | .00 | 2.44 | 1.00 | 2683.00 |
| | | Moods and Feelings | Linear | 0.08 | .01 | .01 | 1.03 | .01 | 17.94** | 1.00 | 2701.00 |
| | | | Quadratic | 0.08 | .01 | .01 | 1.03 | .00 | 1.17 | 1.00 | 2700.00 |
| | | | Cubic | 0.09 | .01 | .01 | 1.03 | .00 | 1.92 | 1.00 | 2699.00 |
| | | Victimisation | Linear | 0.07 | .01 | .01 | .91 | .01 | 14.02** | 1.00 | 2699.00 |
| | | | Quadratic | 0.08 | .01 | .01 | .91 | .00 | 2.44 | 1.00 | 2698.00 |
| | | | Cubic | 0.08 | .01 | .01 | .91 | .00 | 1.41 | 1.00 | 2697.00 |
| | Parent | Behavior Problems | Linear | 0.05 | .00 | .00 | .93 | .00 | 7.57** | 1.00 | 2681.00 |
| | | | Quadratic | 0.05 | .00 | .00 | .93 | .00 | .28 | 1.00 | 2680.00 |
| | | | Cubic | 0.06 | .00 | .00 | .93 | .00 | .92 | 1.00 | 2679.00 |
| | | Moods and Feelings | Linear | 0.05 | .00 | .00 | 1.02 | .00 | 7.55** | 1.00 | 2681.00 |
| | | | Quadratic | 0.05 | .00 | .00 | 1.02 | .00 | .05 | 1.00 | 2680.00 |
| | | | Cubic | 0.06 | .00 | .00 | 1.02 | .00 | 1.34 | 1.00 | 2679.00 |
| | | Antisocial Personality | Linear | 0.04 | .00 | .00 | .92 | .00 | 3.91* | 1.00 | 2691.00 |
| | | | Quadratic | 0.04 | .00 | .00 | .92 | .00 | .12 | 1.00 | 2690.00 |
| | | | Cubic | 0.04 | .00 | .00 | .92 | .00 | .64 | 1.00 | 2689.00 |
| | | Autism | Linear | 0.04 | .00 | .00 | .86 | .00 | 4.82* | 1.00 | 2687.00 |
| | | | Quadratic | 0.05 | .00 | .00 | .86 | .00 | 2.65 | 1.00 | 2686.00 |
| | | | Cubic | 0.07 | .01 | .00 | .86 | .00 | 6.77** | 1.00 | 2685.00 |
| | | ADHD | Linear | 0.02 | .00 | .00 | .84 | .00 | 1.55 | 1.00 | 2687.00 |

| | | | | | | | | | | | |
|---|---|---|---|---|---|---|---|---|---|---|---|
| | | | Quadratic | 0.03 | .00 | .00 | .84 | .00 | .70 | 1.00 | 2686.00 |
| | | | Cubic | 0.03 | .00 | .00 | .85 | .00 | .62 | 1.00 | 2685.00 |
| Age 14 | Self | Victimisation | Linear | 0.11 | .01 | .01 | .90 | .01 | 17.09** | 1.00 | 1503.00 |
| | | | Quadratic | 0.11 | .01 | .01 | .90 | .00 | .02 | 1.00 | 1502.00 |
| | | | Cubic | 0.11 | .01 | .01 | .90 | .00 | .30 | 1.00 | 1501.00 |
| | | ADHD | Linear | 0.05 | .00 | .00 | .91 | .00 | 3.31 | 1.00 | 1510.00 |
| | | | Quadratic | 0.05 | .00 | .00 | .91 | .00 | 1.01 | 1.00 | 1509.00 |
| | | | Cubic | 0.05 | .00 | .00 | .92 | .00 | .05 | 1.00 | 1508.00 |
| | Parent | Victimisation | Linear | 0.10 | .01 | .01 | .93 | .01 | 13.10** | 1.00 | 1342.00 |
| | | | Quadratic | 0.10 | .01 | .01 | .93 | .00 | .90 | 1.00 | 1341.00 |
| | | | Cubic | 0.10 | .01 | .01 | .93 | .00 | .46 | 1.00 | 1340.00 |
| | | Autism | Linear | 0.02 | .00 | .00 | .90 | .00 | .80 | 1.00 | 1464.00 |
| | | | Quadratic | 0.06 | .00 | .00 | .90 | .00 | 4.75* | 1.00 | 1463.00 |
| | | | Cubic | 0.06 | .00 | .00 | .90 | .00 | .03 | 1.00 | 1462.00 |
| | | Antisocial Personality | Linear | 0.04 | .00 | .00 | .95 | .00 | 2.04 | 1.00 | 1473.00 |
| | | | Quadratic | 0.04 | .00 | .00 | .95 | .00 | .06 | 1.00 | 1472.00 |
| | | | Cubic | 0.05 | .00 | .00 | .95 | .00 | 1.99 | 1.00 | 1471.00 |
| | | ADHD | Linear | 0.01 | .00 | .00 | .82 | .00 | .03 | 1.00 | 1481.00 |
| | | | Quadratic | 0.03 | .00 | .00 | .82 | .00 | 1.51 | 1.00 | 1480.00 |
| | | | Cubic | 0.04 | .00 | .00 | .82 | .00 | .23 | 1.00 | 1479.00 |
| Age 16 | Self | Behavior Problems | Linear | 0.05 | .00 | .00 | 1.00 | .00 | 6.00* | 1.00 | 2148.00 |
| | | | Quadratic | 0.06 | .00 | .00 | 1.00 | .00 | .41 | 1.00 | 2147.00 |
| | | | Cubic | 0.06 | .00 | .00 | 1.00 | .00 | .93 | 1.00 | 2146.00 |
| | | Anxiety | Linear | 0.05 | .00 | .00 | 1.06 | .00 | 5.23* | 1.00 | 2150.00 |
| | | | Quadratic | 0.06 | .00 | .00 | 1.06 | .00 | 1.57 | 1.00 | 2149.00 |
| | | | Cubic | 0.06 | .00 | .00 | 1.06 | .00 | 1.63 | 1.00 | 2148.00 |
| | | | Linear | 0.07 | .00 | .00 | 1.10 | .00 | 9.09** | 1.00 | 2150.00 |

| | | | | | | | | | | | |
|---|---|---|---|---|---|---|---|---|---|---|---|
| | | Moods and Feelings | Quadratic | 0.07 | .00 | .00 | 1.10 | .00 | .60 | 1.00 | 2149.00 |
| | | | Cubic | 0.07 | .00 | .00 | 1.10 | .00 | .22 | 1.00 | 2148.00 |
| | | ADHD | Linear | 0.01 | .00 | .00 | 1.04 | .00 | .09 | 1.00 | 520.00 |
| | | | Quadratic | 0.10 | .01 | .01 | 1.03 | .01 | 5.17* | 1.00 | 519.00 |
| | | | Cubic | 0.10 | .01 | .00 | 1.03 | .00 | .07 | 1.00 | 518.00 |
| | | Callous unemotional traits | Linear | 0.02 | .00 | .00 | .84 | .00 | .17 | 1.00 | 518.00 |
| | | | Quadratic | 0.06 | .00 | .00 | .84 | .00 | 1.42 | 1.00 | 517.00 |
| | | | Cubic | 0.06 | .00 | .00 | .84 | .00 | .00 | 1.00 | 516.00 |
| | | Autism | Linear | 0.04 | .00 | .00 | 1.00 | .00 | 3.44 | 1.00 | 2150.00 |
| | | | Quadratic | 0.04 | .00 | .00 | 1.00 | .00 | .61 | 1.00 | 2149.00 |
| | | | Cubic | 0.04 | .00 | .00 | 1.00 | .00 | .03 | 1.00 | 2148.00 |
| | | Anomalous Perceptions | Linear | 0.10 | .01 | .01 | .99 | .01 | 19.50** | 1.00 | 2149.00 |
| | | | Quadratic | 0.10 | .01 | .01 | .99 | .00 | .02 | 1.00 | 2148.00 |
| | | | Cubic | 0.10 | .01 | .01 | .99 | .00 | 1.01 | 1.00 | 2147.00 |
| | | Grandiosity & Delusion | Linear | 0.08 | .01 | .01 | .99 | .00 | 14.01 | 1.00 | 2148.00 |
| | | | Quadratic | 0.08 | .01 | .01 | .99 | .00 | .01 | 1.00 | 2147.00 |
| | | | Cubic | 0.09 | .01 | .01 | .99 | .00 | 1.61 | 1.00 | 2146.00 |
| | | Cognitive disorganisation | Linear | 0.04 | .00 | .00 | .99 | .00 | 3.62 | 1.00 | 2147.00 |
| | | | Quadratic | 0.04 | .00 | .00 | .99 | .00 | .13 | 1.00 | 2146.00 |
| | | | Cubic | 0.05 | .00 | .00 | .99 | .00 | .94 | 1.00 | 2145.00 |
| | | Hedonia | Linear | 0.01 | .00 | .00 | .94 | .00 | .39 | 1.00 | 2151.00 |
| | | | Quadratic | 0.03 | .00 | .00 | .94 | .00 | 1.92 | 1.00 | 2150.00 |
| | | | Cubic | 0.06 | .00 | .00 | .94 | .00 | 4.41* | 1.00 | 2149.00 |
| | | Introvertive Anhedonia | Linear | 0.00 | .00 | .00 | .96 | .00 | .03 | 1.00 | 2147.00 |
| | | | Quadratic | 0.01 | .00 | .00 | .96 | .00 | .41 | 1.00 | 2146.00 |
| | | | Cubic | 0.04 | .00 | .00 | .96 | .00 | 3.28 | 1.00 | 2145.00 |
| | | | Linear | 0.21 | .04 | .04 | .99 | .04 | 23.55** | 1.00 | 516.00 |

| | | | | | | | | | | | |
|---|---|---|---|---|---|---|---|---|---|---|---|
| | | Eating problems | Quadratic | 0.22 | .05 | .05 | .99 | .01 | 2.75 | 1.00 | 515.00 |
| | | | Cubic | 0.22 | .05 | .04 | .99 | .00 | .17 | 1.00 | 514.00 |
| | | Sleep problems | Linear | 0.08 | .01 | .00 | 1.06 | .01 | 9.12** | 1.00 | 1630.00 |
| | | | Quadratic | 0.08 | .01 | .00 | 1.06 | .00 | .26 | 1.00 | 1629.00 |
| | | | Cubic | 0.08 | .01 | .00 | 1.06 | .00 | .29 | 1.00 | 1628.00 |
| | | Victimisation | Linear | 0.08 | .01 | .00 | .97 | .01 | 6.94** | 1.00 | 1187.00 |
| | | | Quadratic | 0.08 | .01 | .00 | .97 | .00 | .21 | 1.00 | 1186.00 |
| | | | Cubic | 0.08 | .01 | .00 | .97 | .00 | .01 | 1.00 | 1185.00 |
| | | Substance use | Linear | 0.07 | .00 | .00 | .95 | .00 | 7.92** | 1.00 | 1629.00** |
| | | | Quadratic | 0.08 | .01 | .00 | .95 | .00 | 1.38 | 1.00 | 1628.00 |
| | | | Cubic | 0.08 | .01 | .00 | .95 | .00 | .47 | 1.00 | 1627.00 |
| | | Delinquency | Linear | 0.07 | .00 | .00 | .95 | .00 | 7.92 | 1.00 | 1629.00 |
| | | | Quadratic | 0.08 | .01 | .00 | .95 | .00 | 1.38 | 1.00 | 1628.00 |
| | | | Cubic | 0.08 | .01 | .00 | .9 | .00 | .47 | 1.00 | 1627.00 |
| | Parent | Behavior Problems | Linear | 0.02 | .00 | .00 | .89 | .00 | .61 | 1.00 | 2151.00 |
| | | | Quadratic | 0.02 | .00 | .00 | .89 | .00 | .00 | 1.00 | 2150.00 |
| | | | Cubic | 0.03 | .00 | .00 | .89 | .00 | 1.32 | 1.00 | 2149.00 |
| | | Anxiety | Linear | 0.03 | .00 | .00 | 1.05 | .00 | 1.54 | 1.00 | 2152.00 |
| | | | Quadratic | 0.04 | .00 | .00 | 1.05 | .00 | 1.75 | 1.00 | 2151.00 |
| | | | Cubic | 0.06 | .00 | .00 | 1.05 | .00 | 4.69* | 1.00 | 2150.00 |
| | | Moods and Feelings | Linear | 0.04 | .00 | .00 | 1.06 | .00 | 3.95 | 1.00 | 2151.00 |
| | | | Quadratic | 0.04 | .00 | .00 | 1.06 | .00 | .056 | 1.00 | 2150.00 |
| | | | Cubic | 0.05 | .00 | .00 | 1.06 | .00 | .578 | 1.00 | 2149.00 |
| | | ADHD | Linear | 0.01 | .00 | .00 | .82 | .00 | .11 | 1.00 | 2148.00 |
| | | | Quadratic | 0.01 | .00 | .00 | .82 | .00 | .01 | 1.00 | 2147.00 |
| | | | Cubic | 0.03 | .00 | .00 | .82 | .00 | 1.67 | 1.00 | 2146.00 |
| | | | Linear | 0.01 | .00 | .00 | .87 | .00 | .06 | 1.00 | 2151.00 |

| | | | | | | | | | | | |
|---|---|---|---|---|---|---|---|---|---|---|---|
| | | Negative Symptoms | Quadratic | 0.01 | .00 | .00 | .87 | .00 | .20 | 1.00 | 2150.00 |
| | | | Cubic | 0.01 | .00 | .00 | .87 | .00 | .08 | 1.00 | 2149.00 |
| | | Callous unemotional traits | Linear | 0.03 | .00 | .00 | .92 | .00 | 1.46 | 1.00 | 2151.00 |
| | | | Quadratic | 0.03 | .00 | .00 | .92 | .00 | .46 | 1.00 | 2150.00 |
| | | | Cubic | 0.03 | .00 | .00 | .92 | .00 | .26 | 1.00 | 2149.00 |
| | | Autism | Linear | 0.01 | .00 | .00 | .90 | .00 | .11 | 1.00 | 2150.00 |
| | | | Quadratic | 0.03 | .00 | .00 | .90 | .00 | 1.44 | 1.00 | 2149.00 |
| | | | Cubic | 0.03 | .00 | .00 | .90 | .00 | .22 | 1.00 | 2148.00 |

Note: $R^2$= proportion of variance explained; Adj $R^2$ = adjusted $R^2$ in new model. Significant non-linear results highlighted in grey. *= p<.05; **= p<.01

***Table S4.2*** - Quadratic and cubic polynomials between boys' age 11 PDS score and behavioral and psychiatric problems concurrently and over time. Analysis was conducted by randomly selecting one twin per pair.

| | | | | | | | | Change statistics | | | |
|---|---|---|---|---|---|---|---|---|---|---|---|
| | | | Model | R | $R^2$ | Adj $R^2$ | Std. Error | $R^2$ Change | F Change | df1 | df2 |
| Age 11 | Self | Behavior Problems | Linear | 0.12 | 0.02 | 0.01 | 0.98 | 0.02 | 34.64** | 1.00 | 2350.00 |
| | | | Quadratic | 0.12 | 0.02 | 0.01 | 0.99 | 0.00 | 0.00 | 1.00 | 2349.00 |
| | | | Cubic | 0.12 | 0.02 | 0.01 | 0.98 | 0.00 | 2.23 | 1.00 | 2348.00 |
| | | Moods and Feelings | Linear | 0.13 | 0.02 | 0.02 | 0.96 | 0.02 | 40.35** | 1.00 | 2354.00 |
| | | | Quadratic | 0.13 | 0.02 | 0.02 | 0.96 | 0.00 | 1.30 | 1.00 | 2353.00 |
| | | | Cubic | 0.14 | 0.02 | 0.02 | 0.96 | 0.00 | 1.89 | 1.00 | 2352.00 |
| | | Victimisation | Linear | 0.14 | 0.02 | 0.02 | 1.02 | 0.02 | 45.93** | 1.00 | 2352.00 |
| | | | Quadratic | 0.14 | 0.02 | 0.02 | 1.02 | 0.00 | 1.53 | 1.00 | 2351.00 |
| | | | Cubic | 0.14 | 0.02 | 0.02 | 1.02 | 0.00 | 2.51 | 1.00 | 2350.00 |
| | Parent | Behavior Problems | Linear | 0.03 | 0.00 | 0.00 | 1.01 | 0.00 | 2.61 | 1.00 | 2334.00 |
| | | | Quadratic | 0.04 | 0.00 | 0.00 | 1.01 | 0.00 | 0.19 | 1.00 | 2333.00 |
| | | | Cubic | 0.04 | 0.00 | 0.00 | 1.01 | 0.00 | 1.59 | 1.00 | 2332.00 |
| | | Moods and Feelings | Linear | 0.00 | 0.00 | 0.00 | 0.97 | 0.00 | 0.01 | 1.00 | 2333.00 |
| | | | Quadratic | 0.01 | 0.00 | 0.00 | 0.97 | 0.00 | 0.16 | 1.00 | 2332.00 |
| | | | Cubic | 0.02 | 0.00 | 0.00 | 0.97 | 0.00 | 0.94 | 1.00 | 2331.00 |
| | | Antisocial Personality | Linear | 0.05 | 0.00 | 0.00 | 1.03 | 0.00 | 5.71* | 1.00 | 2336.00 |
| | | | Quadratic | 0.05 | 0.00 | 0.00 | 1.03 | 0.00 | 0.58 | 1.00 | 2335.00 |
| | | | Cubic | 0.06 | 0.00 | 0.00 | 1.03 | 0.00 | 1.62 | 1.00 | 2334.00 |
| | | Autism | Linear | 0.05 | 0.00 | 0.00 | 0.97 | 0.00 | 5.56* | 1.00 | 2335.00 |
| | | | Quadratic | 0.05 | 0.00 | 0.00 | 0.97 | 0.00 | 1.07 | 1.00 | 2334.00 |
| | | | Cubic | 0.06 | 0.00 | 0.00 | 0.97 | 0.00 | 1.58 | 1.00 | 2333.00 |
| | | ADHD | Linear | 0.04 | 0.00 | 0.00 | 1.03 | 0.00 | 3.69 | 1.00 | 2332.00 |
| | | | Quadratic | 0.04 | 0.00 | 0.00 | 1.03 | 0.00 | 0.76 | 1.00 | 2331.00 |

| | | | | | | | | | | | |
|---|---|---|---|---|---|---|---|---|---|---|---|
| | | | Cubic | 0.05 | 0.00 | 0.00 | 1.03 | 0.00 | 0.56 | 1.00 | 2330.00 |
| Age 14 | Self | Victimisation | Linear | 0.07 | 0.00 | 0.00 | 1.07 | 0.00 | 5.53* | 1.00 | 1229.00 |
| | | | Quadratic | 0.09 | 0.01 | 0.01 | 1.07 | 0.00 | 4.16* | 1.00 | 1228.00 |
| | | | Cubic | 0.09 | 0.01 | 0.01 | 1.07 | 0.00 | 0.06 | 1.00 | 1227.00 |
| | | ADHD | Linear | 0.04 | 0.00 | 0.00 | 0.99 | 0.00 | 2.04 | 1.00 | 1234.00 |
| | | | Quadratic | 0.04 | 0.00 | 0.00 | 0.99 | 0.00 | 0.23 | 1.00 | 1233.00 |
| | | | Cubic | 0.05 | 0.00 | 0.00 | 0.99 | 0.00 | 0.59 | 1.00 | 1232.00 |
| | Parent | Victimisation | Linear | 0.05 | 0.00 | 0.00 | 1.03 | 0.00 | 2.25 | 1.00 | 1100.00 |
| | | | Quadratic | 0.05 | 0.00 | 0.00 | 1.03 | 0.00 | 0.38 | 1.00 | 1099.00 |
| | | | Cubic | 0.05 | 0.00 | 0.00 | 1.03 | 0.00 | 0.60 | 1.00 | 1098.00 |
| | | Autism | Linear | 0.00 | 0.00 | 0.00 | 1.01 | 0.00 | 0.00 | 1.00 | 1267.00 |
| | | | Quadratic | 0.07 | 0.00 | 0.00 | 1.01 | 0.00 | 6.33* | 1.00 | 1266.00 |
| | | | Cubic | 0.08 | 0.01 | 0.00 | 1.01 | 0.00 | 1.55 | 1.00 | 1265.00 |
| | | Antisocial Personality | Linear | 0.05 | 0.00 | 0.00 | 1.02 | 0.00 | 3.15 | 1.00 | 1279.00 |
| | | | Quadratic | 0.06 | 0.00 | 0.00 | 1.02 | 0.00 | 1.47 | 1.00 | 1278.00 |
| | | | Cubic | 0.06 | 0.00 | 0.00 | 1.02 | 0.00 | 0.00 | 1.00 | 1277.00 |
| | | ADHD | Linear | 0.00 | 0.00 | 0.00 | 1.04 | 0.00 | 0.02 | 1.00 | 1288.00 |
| | | | Quadratic | 0.01 | 0.00 | 0.00 | 1.04 | 0.00 | 0.02 | 1.00 | 1287.00 |
| | | | Cubic | 0.01 | 0.00 | 0.00 | 1.04 | 0.00 | 0.17 | 1.00 | 1286.00 |
| Age 16 | Self | Behavior Problems | Linear | 0.07 | 0.00 | 0.00 | 0.94 | 0.00 | 7.42** | 1.00 | 1703.00 |
| | | | Quadratic | 0.07 | 0.01 | 0.00 | 0.94 | 0.00 | 1.13 | 1.00 | 1702.00 |
| | | | Cubic | 0.08 | 0.01 | 0.00 | 0.94 | 0.00 | 1.17 | 1.00 | 1701.00 |
| | | Anxiety | Linear | 0.08 | 0.01 | 0.01 | 0.78 | 0.01 | 11.91** | 1.00 | 1705.00 |
| | | | Quadratic | 0.08 | 0.01 | 0.01 | 0.78 | 0.00 | 0.14 | 1.00 | 1704.00 |
| | | | Cubic | 0.09 | 0.01 | 0.01 | 0.78 | 0.00 | 0.72 | 1.00 | 1703.00 |
| | | Moods and Feelings | Linear | 0.11 | 0.01 | 0.01 | 0.79 | 0.01 | 19.58** | 1.00 | 1705.00 |
| | | | Quadratic | 0.11 | 0.01 | 0.01 | 0.79 | 0.00 | 0.87 | 1.00 | 1704.00 |

| | | | | | | | | | |
|---|---|---|---|---|---|---|---|---|---|
| | Cubic | 0.12 | 0.01 | 0.01 | 0.79 | 0.00 | 5.11* | 1.00 | 1703.00 |
| ADHD | Linear | 0.08 | 0.01 | 0.00 | 0.97 | 0.01 | 2.34 | 1.00 | 345.00 |
| | Quadratic | 0.15 | 0.02 | 0.02 | 0.96 | 0.02 | 5.41* | 1.00 | 344.00 |
| | Cubic | 0.15 | 0.02 | 0.01 | 0.96 | 0.00 | 0.13 | 1.00 | 343.00 |
| Callous unemotional traits | Linear | 0.10 | 0.01 | 0.01 | 1.07 | 0.01 | 3.32 | 1.00 | 344.00 |
| | Quadratic | 0.13 | 0.02 | 0.01 | 1.07 | 0.01 | 2.52 | 1.00 | 343.00 |
| | Cubic | 0.13 | 0.02 | 0.01 | 1.07 | 0.00 | 0.41 | 1.00 | 342.00 |
| Autism | Linear | 0.02 | 0.00 | 0.00 | 0.98 | 0.00 | 1.01 | 1.00 | 1704.00 |
| | Quadratic | 0.03 | 0.00 | 0.00 | 0.98 | 0.00 | 0.79 | 1.00 | 1703.00 |
| | Cubic | 0.03 | 0.00 | 0.00 | 0.99 | 0.00 | 0.22 | 1.00 | 1702.00 |
| Anomalous Perceptions | Linear | 0.14 | 0.02 | 0.02 | 0.94 | 0.02 | 36.34** | 1.00 | 1706.00 |
| | Quadratic | 0.15 | 0.02 | 0.02 | 0.94 | 0.00 | 2.83 | 1.00 | 1705.00 |
| | Cubic | 0.15 | 0.02 | 0.02 | 0.94 | 0.00 | 1.54 | 1.00 | 1704.00 |
| Grandiosity & Delusion | Linear | 0.05 | 0.00 | 0.00 | 0.99 | 0.00 | 4.56* | 1.00 | 1705.00 |
| | Quadratic | 0.05 | 0.00 | 0.00 | 0.99 | 0.00 | 0.39 | 1.00 | 1704.00 |
| | Cubic | 0.05 | 0.00 | 0.00 | 0.99 | 0.00 | 0.00 | 1.00 | 1703.00 |
| Cognitive disorganisation | Linear | 0.06 | 0.00 | 0.00 | 0.94 | 0.00 | 5.79* | 1.00 | 1702.00 |
| | Quadratic | 0.06 | 0.00 | 0.00 | 0.94 | 0.00 | 0.25 | 1.00 | 1701.00 |
| | Cubic | 0.06 | 0.00 | 0.00 | 0.94 | 0.00 | 1.04 | 1.00 | 1700.00 |
| Hedonia | Linear | 0.04 | 0.00 | 0.00 | 1.02 | 0.00 | 2.47 | 1.00 | 1702.00 |
| | Quadratic | 0.04 | 0.00 | 0.00 | 1.02 | 0.00 | 0.68 | 1.00 | 1701.00 |
| | Cubic | 0.05 | 0.00 | 0.00 | 1.02 | 0.00 | 0.60 | 1.00 | 1700.00 |
| Introvertive Anhedonia | Linear | 0.01 | 0.00 | 0.00 | 0.99 | 0.00 | 0.23 | 1.00 | 1703.00 |
| | Quadratic | 0.01 | 0.00 | 0.00 | 0.99 | 0.00 | 0.02 | 1.00 | 1702.00 |
| | Cubic | 0.02 | 0.00 | 0.00 | 0.99 | 0.00 | 0.70 | 1.00 | 1701.00 |
| Eating problems | Linear | 0.07 | 0.01 | 0.00 | 0.70 | 0.01 | 1.75 | 1.00 | 343.00 |
| | Quadratic | 0.09 | 0.01 | 0.00 | 0.70 | 0.00 | 1.29 | 1.00 | 342.00 |

| | | | | | | | | | | | |
|---|---|---|---|---|---|---|---|---|---|---|---|
| | | | Cubic | 0.09 | 0.01 | 0.00 | 0.70 | 0.00 | 0.01 | 1.00 | 341.00 |
| | | Sleep problems | Linear | 0.11 | 0.01 | 0.01 | 0.93 | 0.01 | 17.02** | 1.00 | 1358.00 |
| | | | Quadratic | 0.11 | 0.01 | 0.01 | 0.93 | 0.00 | 0.85 | 1.00 | 1357.00 |
| | | | Cubic | 0.12 | 0.01 | 0.01 | 0.93 | 0.00 | 0.20 | 1.00 | 1356.00 |
| | | Victimisation | Linear | 0.04 | 0.00 | 0.00 | 1.01 | 0.00 | 1.15 | 1.00 | 863.00 |
| | | | Quadratic | 0.04 | 0.00 | 0.00 | 1.01 | 0.00 | 0.00 | 1.00 | 862.00 |
| | | | Cubic | 0.06 | 0.00 | 0.00 | 1.01 | 0.00 | 2.08 | 1.00 | 861.00 |
| | | Substance use | Linear | 0.09 | 0.01 | 0.01 | 1.03 | 0.01 | 11.04** | 1.00 | 1359.00 |
| | | | Quadratic | 0.10 | 0.01 | 0.01 | 1.03 | 0.00 | 1.24 | 1.00 | 1358.00 |
| | | | Cubic | 0.10 | 0.01 | 0.01 | 1.03 | 0.00 | 1.05 | 1.00 | 1357.00 |
| | | Delinquency | Linear | 0.09 | 0.01 | 0.01 | 1.03 | 0.01 | 11.04** | 1.00 | 1359.00 |
| | | | Quadratic | 0.10 | 0.01 | 0.01 | 1.03 | 0.00 | 1.24 | 1.00 | 1358.00 |
| | | | Cubic | 0.10 | 0.01 | 0.01 | 1.03 | 0.00 | 1.05 | 1.00 | 1357.00 |
| | Parent | Behavior Problems | Linear | 0.01 | 0.00 | 0.00 | 1.00 | 0.00 | 0.16 | 1.00 | 1713.00 |
| | | | Quadratic | 0.02 | 0.00 | 0.00 | 1.00 | 0.00 | 0.81 | 1.00 | 1712.00 |
| | | | Cubic | 0.02 | 0.00 | 0.00 | 1.00 | 0.00 | 0.02 | 1.00 | 1711.00 |
| | | Anxiety | Linear | 0.00 | 0.00 | 0.00 | 0.81 | 0.00 | 0.01 | 1.00 | 1715.00 |
| | | | Quadratic | 0.01 | 0.00 | 0.00 | 0.81 | 0.00 | 0.09 | 1.00 | 1714.00 |
| | | | Cubic | 0.01 | 0.00 | 0.00 | 0.81 | 0.00 | 0.19 | 1.00 | 1713.00 |
| | | Moods and Feelings | Linear | 0.01 | 0.00 | 0.00 | 0.78 | 0.00 | 0.36 | 1.00 | 1713.00 |
| | | | Quadratic | 0.02 | 0.00 | 0.00 | 0.78 | 0.00 | 0.02 | 1.00 | 1712.00 |
| | | | Cubic | 0.02 | 0.00 | 0.00 | 0.78 | 0.00 | 0.09 | 1.00 | 1711.00 |
| | | ADHD | Linear | 0.03 | 0.00 | 0.00 | 1.03 | 0.00 | 1.30 | 1.00 | 1715.00 |
| | | | Quadratic | 0.03 | 0.00 | 0.00 | 1.03 | 0.00 | 0.20 | 1.00 | 1714.00 |
| | | | Cubic | 0.03 | 0.00 | 0.00 | 1.03 | 0.00 | 0.19 | 1.00 | 1713.00 |
| | | Negative Symptoms | Linear | 0.01 | 0.00 | 0.00 | 1.01 | 0.00 | 0.09 | 1.00 | 1711.00 |
| | | | Quadratic | 0.04 | 0.00 | 0.00 | 1.01 | 0.00 | 2.43 | 1.00 | 1710.00 |

| | | | | | | | | | | | |
|---|---|---|---|---|---|---|---|---|---|---|---|
| | | | Cubic | 0.05 | 0.00 | 0.00 | 1.01 | 0.00 | 1.28 | 1.00 | 1709.00 |
| | | Callous unemotional traits | Linear | 0.03 | 0.00 | 0.00 | 1.00 | 0.00 | 1.45 | 1.00 | 1713.00 |
| | | | Quadratic | 0.03 | 0.00 | 0.00 | 1.00 | 0.00 | 0.27 | 1.00 | 1712.00 |
| | | | Cubic | 0.03 | 0.00 | 0.00 | 1.00 | 0.00 | 0.09 | 1.00 | 1711.00 |
| | | Autism | Linear | 0.01 | 0.00 | 0.00 | 1.00 | 0.00 | 0.34 | 1.00 | 1713.00 |
| | | | Quadratic | 0.05 | 0.00 | 0.00 | 1.00 | 0.00 | 3.19 | 1.00 | 1712.00 |
| | | | Cubic | 0.06 | 0.00 | 0.00 | 1.00 | 0.00 | 1.75 | 1.00 | 1711.00 |

Note: $R^2$= proportion of variance explained; Adj $R^2$ = adjusted $R^2$ in new model. Significant non-linear results highlighted in grey. *= p<.05; **= p<.01

***Table S4.3*** - Linear, quadratic and cubic associations between girls' age 14 PDS score and behavioral and psychiatric problems concurrently and over time. Analysis was conducted by randomly selecting one twin per pair.

| | | | | | | | | Change statistics | | | |
|---|---|---|---|---|---|---|---|---|---|---|---|
| | | | Model | R | $R^2$ | Adj $R^2$ | Std. Error | $R^2$ Change | F Change | df1 | df2 |
| Age 14 | Self | Victimisation | Linear | 0.00 | 0.00 | 0.00 | 0.91 | 0.00 | 0.00 | 1.00 | 1583.00 |
| | | | Quadratic | 0.01 | 0.00 | 0.00 | 0.91 | 0.00 | 0.09 | 1.00 | 1582.00 |
| | | | Cubic | 0.01 | 0.00 | 0.00 | 0.91 | 0.00 | 0.23 | 1.00 | 1581.00 |
| | | ADHD | Linear | 0.03 | 0.00 | 0.00 | 0.93 | 0.00 | 1.31 | 1.00 | 1585.00 |
| | | | Quadratic | 0.03 | 0.00 | 0.00 | 0.93 | 0.00 | 0.06 | 1.00 | 1584.00 |
| | | | Cubic | 0.03 | 0.00 | 0.00 | 0.93 | 0.00 | 0.00 | 1.00 | 1583.00 |
| | Parent | Victimisation | Linear | 0.03 | 0.00 | 0.00 | 0.94 | 0.00 | 0.82 | 1.00 | 1326.00 |
| | | | Quadratic | 0.03 | 0.00 | 0.00 | 0.94 | 0.00 | 0.36 | 1.00 | 1325.00 |
| | | | Cubic | 0.03 | 0.00 | 0.00 | 0.94 | 0.00 | 0.02 | 1.00 | 1324.00 |
| | | Autism | Linear | 0.04 | 0.00 | 0.00 | 0.90 | 0.00 | 2.07 | 1.00 | 1450.00 |
| | | | Quadratic | 0.08 | 0.01 | 0.00 | 0.90 | 0.00 | 6.95** | 1.00 | 1449.00 |
| | | | Cubic | 0.08 | 0.01 | 0.00 | 0.90 | 0.00 | 0.08 | 1.00 | 1448.00 |
| | | Antisocial Personality | Linear | 0.03 | 0.00 | 0.00 | 0.92 | 0.00 | 0.92 | 1.00 | 1459.00 |
| | | | Quadratic | 0.03 | 0.00 | 0.00 | 0.92 | 0.00 | 0.21 | 1.00 | 1458.00 |
| | | | Cubic | 0.05 | 0.00 | 0.00 | 0.92 | 0.00 | 1.77 | 1.00 | 1457.00 |
| | | ADHD | Linear | 0.01 | 0.00 | 0.00 | 0.82 | 0.00 | 0.21 | 1.00 | 1464.00 |
| | | | Quadratic | 0.01 | 0.00 | 0.00 | 0.82 | 0.00 | 0.01 | 1.00 | 1463.00 |
| | | | Cubic | 0.05 | 0.00 | 0.00 | 0.82 | 0.00 | 3.53 | 1.00 | 1462.00 |
| Age 16 | Self | Behavior Problems | Linear | 0.02 | 0.00 | 0.00 | 0.97 | 0.00 | 0.40 | 1.00 | 1355.00 |
| | | | Quadratic | 0.04 | 0.00 | 0.00 | 0.97 | 0.00 | 1.56 | 1.00 | 1354.00 |
| | | | Cubic | 0.04 | 0.00 | 0.00 | 0.97 | 0.00 | 0.24 | 1.00 | 1353.00 |
| | | Anxiety | Linear | 0.04 | 0.00 | 0.00 | 1.04 | 0.00 | 1.82 | 1.00 | 1358.00 |
| | | | Quadratic | 0.04 | 0.00 | 0.00 | 1.04 | 0.00 | 0.59 | 1.00 | 1357.00 |

| | | | | | | | | | | | |
|---|---|---|---|---|---|---|---|---|---|---|---|
| | | | Cubic | 0.05 | 0.00 | 0.00 | 1.04 | 0.00 | 0.46 | 1.00 | 1356.00 |
| | | Moods and Feelings | Linear | 0.05 | 0.00 | 0.00 | 1.07 | 0.00 | 3.79 | 1.00 | 1357.00 |
| | | | Quadratic | 0.07 | 0.00 | 0.00 | 1.07 | 0.00 | 1.93 | 1.00 | 1356.00 |
| | | | Cubic | 0.07 | 0.00 | 0.00 | 1.07 | 0.00 | 0.00 | 1.00 | 1355.00 |
| | | ADHD | Linear | 0.06 | 0.00 | 0.00 | 1.02 | 0.00 | 1.68 | 1.00 | 470.00 |
| | | | Quadratic | 0.06 | 0.00 | 0.00 | 1.02 | 0.00 | 0.09 | 1.00 | 469.00 |
| | | | Cubic | 0.08 | 0.01 | 0.00 | 1.02 | 0.00 | 0.91 | 1.00 | 468.00 |
| | | Callous unemotional traits | Linear | 0.07 | 0.01 | 0.00 | 0.83 | 0.01 | 2.55 | 1.00 | 468.00 |
| | | | Quadratic | 0.11 | 0.01 | 0.01 | 0.82 | 0.01 | 3.55 | 1.00 | 467.00 |
| | | | Cubic | 0.11 | 0.01 | 0.01 | 0.83 | 0.00 | 0.03 | 1.00 | 466.00 |
| | | Autism | Linear | 0.01 | 0.00 | 0.00 | 1.01 | 0.00 | 0.05 | 1.00 | 1356.00 |
| | | | Quadratic | 0.04 | 0.00 | 0.00 | 1.01 | 0.00 | 1.98 | 1.00 | 1355.00 |
| | | | Cubic | 0.04 | 0.00 | 0.00 | 1.01 | 0.00 | 0.04 | 1.00 | 1354.00 |
| | | Anomalous Perceptions | Linear | 0.10 | 0.01 | 0.01 | 1.00 | 0.00 | 19.50* | 1.00 | 2149.00 |
| | | | Quadratic | 0.10 | 0.01 | 0.02 | 1.00 | 0.00 | 0.17 | 1.00 | 2148.00 |
| | | | Cubic | 0.10 | 0.01 | 0.02 | 1.00 | 0.00 | 1.01 | 1.00 | 2147.00 |
| | | Grandiosity & Delusion | Linear | 0.06 | 0.00 | 0.00 | 0.92 | 0.00 | 5.60* | 1.00 | 1356.00 |
| | | | Quadratic | 0.08 | 0.01 | 0.00 | 0.92 | 0.00 | 2.08 | 1.00 | 1355.00 |
| | | | Cubic | 0.10 | 0.01 | 0.01 | 0.91 | 0.00 | 6.55* | 1.00 | 1354.00 |
| | | Cognitive disorganisation | Linear | 0.01 | 0.00 | 0.00 | 0.98 | 0.00 | 0.16 | 1.00 | 1356.00 |
| | | | Quadratic | 0.02 | 0.00 | 0.00 | 0.98 | 0.00 | 0.37 | 1.00 | 1355.00 |
| | | | Cubic | 0.03 | 0.00 | 0.00 | 0.98 | 0.00 | 0.63 | 1.00 | 1354.00 |
| | | Hedonia | Linear | 0.02 | 0.00 | 0.00 | 0.92 | 0.00 | 0.51 | 1.00 | 1358.00 |
| | | | Quadratic | 0.07 | 0.00 | 0.00 | 0.92 | 0.00 | 5.29* | 1.00 | 1357.00 |
| | | | Cubic | 0.07 | 0.00 | 0.00 | 0.92 | 0.00 | 0.39 | 1.00 | 1356.00 |
| | | Introvertive Anhedonia | Linear | 0.01 | 0.00 | 0.00 | 0.95 | 0.00 | 0.21 | 1.00 | 1356.00 |
| | | | Quadratic | 0.03 | 0.00 | 0.00 | 0.95 | 0.00 | 1.12 | 1.00 | 1355.00 |

| | | | | | | | | | | | |
|---|---|---|---|---|---|---|---|---|---|---|---|
| | | | Cubic | 0.04 | 0.00 | 0.00 | 0.95 | 0.00 | 0.42 | 1.00 | 1354.00 |
| | | Eating problems | Linear | 0.11 | 0.01 | 0.01 | 0.99 | 0.01 | 5.49* | 1.00 | 465.00 |
| | | | Quadratic | 0.11 | 0.01 | 0.01 | 0.99 | 0.00 | 0.63 | 1.00 | 464.00 |
| | | | Cubic | 0.14 | 0.02 | 0.01 | 0.99 | 0.01 | 3.33 | 1.00 | 463.00 |
| | | Sleep problems | Linear | 0.03 | 0.00 | 0.00 | 1.06 | 0.00 | 0.99 | 1.00 | 887.00 |
| | | | Quadratic | 0.06 | 0.00 | 0.00 | 1.06 | 0.00 | 2.38 | 1.00 | 886.00 |
| | | | Cubic | 0.07 | 0.00 | 0.00 | 1.06 | 0.00 | 0.65 | 1.00 | 885.00 |
| | | Victimisation | Linear | 0.03 | 0.00 | 0.00 | 0.98 | 0.00 | 0.97 | 1.00 | 902.00 |
| | | | Quadratic | 0.04 | 0.00 | 0.00 | 0.98 | 0.00 | 0.37 | 1.00 | 901.00 |
| | | | Cubic | 0.04 | 0.00 | 0.00 | 0.98 | 0.00 | 0.40 | 1.00 | 900.00 |
| | | Substance use | Linear | 0.06 | 0.00 | 0.00 | 0.89 | 0.00 | 3.53 | 1.00 | 886.00 |
| | | | Quadratic | 0.06 | 0.00 | 0.00 | 0.89 | 0.00 | 0.12 | 1.00 | 885.00 |
| | | | Cubic | 0.06 | 0.00 | 0.00 | 0.89 | 0.00 | 0.02 | 1.00 | 884.00 |
| | | Delinquency | Linear | 0.08 | 0.01 | 0.00 | 0.89 | 0.01 | 2.89 | 1.00 | 469.00 |
| | | | Quadratic | 0.08 | 0.01 | 0.00 | 0.89 | 0.00 | 0.01 | 1.00 | 468.00 |
| | | | Cubic | 0.08 | 0.01 | 0.00 | 0.89 | 0.00 | 0.10 | 1.00 | 467.00 |
| | Parent | Behavior Problems | Linear | 0.01 | 0.00 | 0.00 | 0.88 | 0.00 | 0.11 | 1.00 | 1354.00 |
| | | | Quadratic | 0.01 | 0.00 | 0.00 | 0.88 | 0.00 | 0.02 | 1.00 | 1353.00 |
| | | | Cubic | 0.02 | 0.00 | 0.00 | 0.88 | 0.00 | 0.35 | 1.00 | 1352.00 |
| | | Anxiety | Linear | 0.04 | 0.00 | 0.00 | 0.97 | 0.00 | 1.93 | 1.00 | 1356.00 |
| | | | Quadratic | 0.04 | 0.00 | 0.00 | 0.97 | 0.00 | 0.67 | 1.00 | 1355.00 |
| | | | Cubic | 0.04 | 0.00 | 0.00 | 0.97 | 0.00 | 0.03 | 1.00 | 1354.00 |
| | | Moods and Feelings | Linear | 0.04 | 0.00 | 0.00 | 1.00 | 0.00 | 2.23 | 1.00 | 1355.00 |
| | | | Quadratic | 0.04 | 0.00 | 0.00 | 1.00 | 0.00 | 0.16 | 1.00 | 1354.00 |
| | | | Cubic | 0.04 | 0.00 | 0.00 | 1.00 | 0.00 | 0.02 | 1.00 | 1353.00 |
| | | ADHD | Linear | 0.01 | 0.00 | 0.00 | 0.77 | 0.00 | 0.11 | 1.00 | 1352.00 |
| | | | Quadratic | 0.01 | 0.00 | 0.00 | 0.77 | 0.00 | 0.05 | 1.00 | 1351.00 |

| | | | | | | | | | | | |
|---|---|---|---|---|---|---|---|---|---|---|---|
| | | | Cubic | 0.03 | 0.00 | 0.00 | 0.77 | 0.00 | 1.40 | 1.00 | 1350.00 |
| | | Negative Symptoms | Linear | 0.02 | 0.00 | 0.00 | 0.84 | 0.00 | 0.58 | 1.00 | 1356.00 |
| | | | Quadratic | 0.03 | 0.00 | 0.00 | 0.84 | 0.00 | 0.77 | 1.00 | 1355.00 |
| | | | Cubic | 0.04 | 0.00 | 0.00 | 0.84 | 0.00 | 0.76 | 1.00 | 1354.00 |
| | | Callous unemotional traits | Linear | 0.04 | 0.00 | 0.00 | 0.91 | 0.00 | 2.68 | 1.00 | 1355.00 |
| | | | Quadratic | 0.05 | 0.00 | 0.00 | 0.91 | 0.00 | 0.38 | 1.00 | 1354.00 |
| | | | Cubic | 0.05 | 0.00 | 0.00 | 0.91 | 0.00 | 0.32 | 1.00 | 1353.00 |
| | | Autism | Linear | 0.02 | 0.00 | 0.00 | 0.91 | 0.00 | 0.56 | 1.00 | 1353.00 |
| | | | Quadratic | 0.06 | 0.00 | 0.00 | 0.91 | 0.00 | 4.81* | 1.00 | 1352.00 |
| | | | Cubic | 0.06 | 0.00 | 0.00 | 0.91 | 0.00 | 0.02 | 1.00 | 1351.00 |

Note: $R^2$= proportion of variance explained; Adj $R^2$ = adjusted $R^2$ in new model. Significant non-linear results highlighted in grey. *= p<.05; **= p<.01

***Table S4.4*** - Linear, quadratic and cubic associations between boys' age 14 PDS score and behavioral and psychiatric problems concurrently and over time. Analysis was conducted by randomly selecting one twin per pair.

| | | | | | | | | Change statistics | | | |
|---|---|---|---|---|---|---|---|---|---|---|---|
| | | | Model | R | $R^2$ | Adj $R^2$ | Std. Error | $R^2$ Change | F Change | df1 | df2 |
| Age 14 | Self | Victimisation | Linear | 0.05 | 0.00 | 0.00 | 1.08 | 0.00 | 3.36 | 1.00 | 1314.00 |
| | | | Quadratic | 0.09 | 0.01 | 0.01 | 1.08 | 0.01 | 7.21** | 1.00 | 1313.00 |
| | | | Cubic | 0.10 | 0.01 | 0.01 | 1.08 | 0.00 | 1.31 | 1.00 | 1312.00 |
| | | ADHD | Linear | 0.06 | 0.00 | 0.00 | 1.01 | 0.00 | 4.15* | 1.00 | 1313.00 |
| | | | Quadratic | 0.07 | 0.01 | 0.00 | 1.01 | 0.00 | 2.53 | 1.00 | 1312.00 |
| | | | Cubic | 0.08 | 0.01 | 0.00 | 1.01 | 0.00 | 1.56 | 1.00 | 1311.00 |
| | Parent | Victimisation | Linear | 0.05 | 0.00 | 0.00 | 1.02 | 0.00 | 2.52 | 1.00 | 1082.00 |
| | | | Quadratic | 0.08 | 0.01 | 0.00 | 1.02 | 0.00 | 4.64** | 1.00 | 1081.00 |
| | | | Cubic | 0.08 | 0.01 | 0.00 | 1.02 | 0.00 | 0.13 | 1.00 | 1080.00 |
| | | Autism | Linear | 0.08 | 0.01 | 0.01 | 1.00 | 0.01 | 8.90** | 1.00 | 1240.00 |
| | | | Quadratic | 0.09 | 0.01 | 0.01 | 1.00 | 0.00 | 1.25 | 1.00 | 1239.00 |
| | | | Cubic | 0.09 | 0.01 | 0.01 | 1.00 | 0.00 | 0.04 | 1.00 | 1238.00 |
| | | Antisocial Personality | Linear | 0.01 | 0.00 | 0.00 | 1.02 | 0.00 | 0.05 | 1.00 | 1250.00 |
| | | | Quadratic | 0.04 | 0.00 | 0.00 | 1.02 | 0.00 | 2.00 | 1.00 | 1249.00 |
| | | | Cubic | 0.04 | 0.00 | 0.00 | 1.02 | 0.00 | 0.11 | 1.00 | 1248.00 |
| | | ADHD | Linear | 0.08 | 0.01 | 0.01 | 1.03 | 0.01 | 7.48** | 1.00 | 1256.00 |
| | | | Quadratic | 0.08 | 0.01 | 0.01 | 1.03 | 0.00 | 1.33 | 1.00 | 1255.00 |
| | | | Cubic | 0.09 | 0.01 | 0.01 | 1.03 | 0.00 | 2.11 | 1.00 | 1254.00 |
| Age 16 | Self | Behavior Problems | Linear | 0.01 | 0.00 | 0.00 | 0.94 | 0.00 | 0.21 | 1.00 | 1046.00 |
| | | | Quadratic | 0.04 | 0.00 | 0.00 | 0.94 | 0.00 | 1.33 | 1.00 | 1045.00 |
| | | | Cubic | 0.05 | 0.00 | 0.00 | 0.94 | 0.00 | 0.66 | 1.00 | 1044.00 |
| | | Anxiety | Linear | 0.00 | 0.00 | 0.00 | 0.76 | 0.00 | 0.01 | 1.00 | 1047.00 |
| | | | Quadratic | 0.05 | 0.00 | 0.00 | 0.76 | 0.00 | 2.23 | 1.00 | 1046.00 |

| | | | | | | | | | | | |
|---|---|---|---|---|---|---|---|---|---|---|---|
| | | | Cubic | 0.05 | 0.00 | 0.00 | 0.76 | 0.00 | 0.07 | 1.00 | 1045.00 |
| | | Moods and Feelings | Linear | 0.05 | 0.00 | 0.00 | 0.80 | 0.00 | 2.41 | 1.00 | 1047.00 |
| | | | Quadratic | 0.07 | 0.01 | 0.00 | 0.80 | 0.00 | 3.09 | 1.00 | 1046.00 |
| | | | Cubic | 0.07 | 0.01 | 0.00 | 0.80 | 0.00 | 0.02 | 1.00 | 1045.00 |
| | | ADHD | Linear | 0.02 | 0.00 | 0.00 | 0.98 | 0.00 | 0.09 | 1.00 | 315.00 |
| | | | Quadratic | 0.07 | 0.00 | 0.00 | 0.98 | 0.00 | 1.25 | 1.00 | 314.00 |
| | | | Cubic | 0.07 | 0.00 | -0.01 | 0.98 | 0.00 | 0.08 | 1.00 | 313.00 |
| | | Callous unemotional traits | Linear | 0.10 | 0.01 | 0.01 | 1.06 | 0.01 | 2.91 | 1.00 | 314.00 |
| | | | Quadratic | 0.10 | 0.01 | 0.00 | 1.07 | 0.00 | 0.10 | 1.00 | 313.00 |
| | | | Cubic | 0.10 | 0.01 | 0.00 | 1.07 | 0.00 | 0.17 | 1.00 | 312.00 |
| | | Autism | Linear | 0.00 | 0.00 | 0.00 | 0.99 | 0.00 | 0.02 | 1.00 | 1047.00 |
| | | | Quadratic | 0.03 | 0.00 | 0.00 | 0.99 | 0.00 | 1.01 | 1.00 | 1046.00 |
| | | | Cubic | 0.04 | 0.00 | 0.00 | 0.99 | 0.00 | 0.92 | 1.00 | 1045.00 |
| | | Anomalous Perceptions | Linear | 0.14 | 0.02 | 0.02 | 0.96 | 0.00 | 3.68 | 1.00 | 1706.00 |
| | | | Quadratic | 0.15 | 0.02 | 0.02 | 0.96 | 0.00 | 2.83 | 1.00 | 1705.00 |
| | | | Cubic | 0.15 | 0.02 | 0.02 | 0.96 | 0.00 | 1.54 | 1.00 | 1704.00 |
| | | Grandiosity & Delusion | Linear | 0.01 | 0.00 | 0.00 | 1.01 | 0.00 | 0.13 | 1.00 | 1047.00 |
| | | | Quadratic | 0.04 | 0.00 | 0.00 | 1.01 | 0.00 | 1.38 | 1.00 | 1046.00 |
| | | | Cubic | 0.06 | 0.00 | 0.00 | 1.01 | 0.00 | 1.70 | 1.00 | 1045.00 |
| | | Cognitive disorganisation | Linear | 0.03 | 0.00 | 0.00 | 0.91 | 0.00 | 1.11 | 1.00 | 1047.00 |
| | | | Quadratic | 0.03 | 0.00 | 0.00 | 0.91 | 0.00 | 0.06 | 1.00 | 1046.00 |
| | | | Cubic | 0.04 | 0.00 | 0.00 | 0.91 | 0.00 | 0.09 | 1.00 | 1045.00 |
| | | Hedonia | Linear | 0.04 | 0.00 | 0.00 | 0.98 | 0.00 | 1.47 | 1.00 | 1045.00 |
| | | | Quadratic | 0.04 | 0.00 | 0.00 | 0.98 | 0.00 | 0.57 | 1.00 | 1044.00 |
| | | | Cubic | 0.05 | 0.00 | 0.00 | 0.98 | 0.00 | 0.21 | 1.00 | 1043.00 |
| | | Introvertive Anhedonia | Linear | 0.00 | 0.00 | 0.00 | 0.98 | 0.00 | 0.01 | 1.00 | 1046.00 |
| | | | Quadratic | 0.06 | 0.00 | 0.00 | 0.98 | 0.00 | 4.00* | 1.00 | 1045.00 |

| | | | | | | | | | | | |
|---|---|---|---|---|---|---|---|---|---|---|---|
| | | | Cubic | 0.07 | 0.00 | 0.00 | 0.98 | 0.00 | 0.52 | 1.00 | 1044.00 |
| | | Eating problems | Linear | 0.04 | 0.00 | 0.00 | 0.74 | 0.00 | 0.60 | 1.00 | 314.00 |
| | | | Quadratic | 0.09 | 0.01 | 0.00 | 0.74 | 0.01 | 1.93 | 1.00 | 313.00 |
| | | | Cubic | 0.11 | 0.01 | 0.00 | 0.74 | 0.00 | 1.12 | 1.00 | 312.00 |
| | | Sleep problems | Linear | 0.12 | 0.02 | 0.01 | 0.90 | 0.02 | 11.32** | 1.00 | 730.00 |
| | | | Quadratic | 0.13 | 0.02 | 0.01 | 0.90 | 0.00 | 0.78 | 1.00 | 729.00 |
| | | | Cubic | 0.13 | 0.02 | 0.01 | 0.90 | 0.00 | 0.18 | 1.00 | 728.00 |
| | | Victimisation | Linear | 0.06 | 0.00 | 0.00 | 1.00 | 0.00 | 2.51 | 1.00 | 688.00 |
| | | | Quadratic | 0.08 | 0.01 | 0.00 | 1.00 | 0.00 | 1.89 | 1.00 | 687.00 |
| | | | Cubic | 0.08 | 0.01 | 0.00 | 1.00 | 0.00 | 0.03 | 1.00 | 686.00 |
| | | Substance use | Linear | 0.04 | 0.00 | 0.00 | 1.00 | 0.00 | 1.04 | 1.00 | 731.00 |
| | | | Quadratic | 0.04 | 0.00 | 0.00 | 1.00 | 0.00 | 0.02 | 1.00 | 730.00 |
| | | | Cubic | 0.06 | 0.00 | 0.00 | 1.00 | 0.00 | 1.69 | 1.00 | 729.00 |
| | | Delinquency | Linear | 0.03 | 0.00 | 0.00 | 1.03 | 0.00 | 0.32 | 1.00 | 315.00 |
| | | | Quadratic | 0.07 | 0.00 | 0.00 | 1.03 | 0.00 | 1.07 | 1.00 | 314.00 |
| | | | Cubic | 0.12 | 0.01 | 0.00 | 1.03 | 0.01 | 3.18 | 1.00 | 313.00 |
| | Parent | Behavior Problems | Linear | 0.07 | 0.00 | 0.00 | 0.94 | 0.00 | 5.29* | 1.00 | 1056.00 |
| | | | Quadratic | 0.07 | 0.01 | 0.00 | 0.94 | 0.00 | 0.22 | 1.00 | 1055.00 |
| | | | Cubic | 0.08 | 0.01 | 0.00 | 0.94 | 0.00 | 0.96 | 1.00 | 1054.00 |
| | | Anxiety | Linear | 0.04 | 0.00 | 0.00 | 0.79 | 0.00 | 1.81 | 1.00 | 1056.00 |
| | | | Quadratic | 0.05 | 0.00 | 0.00 | 0.79 | 0.00 | 0.49 | 1.00 | 1055.00 |
| | | | Cubic | 0.05 | 0.00 | 0.00 | 0.79 | 0.00 | 0.74 | 1.00 | 1054.00 |
| | | Moods and Feelings | Linear | 0.01 | 0.00 | 0.00 | 0.80 | 0.00 | 0.14 | 1.00 | 1055.00 |
| | | | Quadratic | 0.02 | 0.00 | 0.00 | 0.80 | 0.00 | 0.46 | 1.00 | 1054.00 |
| | | | Cubic | 0.02 | 0.00 | 0.00 | 0.80 | 0.00 | 0.01 | 1.00 | 1053.00 |
| | | ADHD | Linear | 0.08 | 0.01 | 0.01 | 0.97 | 0.01 | 6.76** | 1.00 | 1055.00 |
| | | | Quadratic | 0.08 | 0.01 | 0.00 | 0.98 | 0.00 | 0.21 | 1.00 | 1054.00 |

| | | | | | | | | | | | |
|---|---|---|---|---|---|---|---|---|---|---|---|
| | | | Cubic | 0.08 | 0.01 | 0.00 | 0.98 | 0.00 | 0.31 | 1.00 | 1053.00 |
| | | Negative Symptoms | Linear | 0.05 | 0.00 | 0.00 | 1.00 | 0.00 | 2.67 | 1.00 | 1053.00 |
| | | | Quadratic | 0.06 | 0.00 | 0.00 | 1.00 | 0.00 | 0.65 | 1.00 | 1052.00 |
| | | | Cubic | 0.06 | 0.00 | 0.00 | 1.00 | 0.00 | 0.00 | 1.00 | 1051.00 |
| | | Callous unemotional traits | Linear | 0.00 | 0.00 | 0.00 | 0.99 | 0.00 | 0.00 | 1.00 | 1055.00 |
| | | | Quadratic | 0.04 | 0.00 | 0.00 | 0.99 | 0.00 | 1.44 | 1.00 | 1054.00 |
| | | | Cubic | 0.04 | 0.00 | 0.00 | 0.99 | 0.00 | 0.03 | 1.00 | 1053.00 |
| | | Autism | Linear | 0.04 | 0.00 | 0.00 | 1.01 | 0.00 | 1.55 | 1.00 | 1055.00 |
| | | | Quadratic | 0.04 | 0.00 | 0.00 | 1.01 | 0.00 | 0.46 | 1.00 | 1054.00 |
| | | | Cubic | 0.04 | 0.00 | 0.00 | 1.01 | 0.00 | 0.03 | 1.00 | 1053.00 |

Note: $R^2$= proportion of variance explained; Adj $R^2$ = adjusted $R^2$ in new model. Significant non-linear results highlighted in grey. *= p<.05; **= p<.01

***Table S4.5*** - Linear, quadratic and cubic associations between girls' age 16 PDS score and behavioral and psychiatric problems concurrently and over time. Analysis was conducted by randomly selecting one twin per pair.

| | | | | | | | | Change statistics | | | |
|---|---|---|---|---|---|---|---|---|---|---|---|
| | | | Model | R | $R^2$ | Adj $R^2$ | Std. Error | $R^2$ Change | F Change | df1 | df2 |
| Age 16 | Self | Behavior Problems | Linear | 0.03 | 0.00 | 0.00 | 0.95 | 0.00 | 1.08 | 1.00 | 1061.00 |
| | | | Quadratic | 0.04 | 0.00 | 0.00 | 0.95 | 0.00 | 0.49 | 1.00 | 1060.00 |
| | | | Cubic | 0.04 | 0.00 | 0.00 | 0.95 | 0.00 | 0.28 | 1.00 | 1059.00 |
| | | Anxiety | Linear | 0.04 | 0.00 | 0.00 | 1.03 | 0.00 | 2.01 | 1.00 | 1063.00 |
| | | | Quadratic | 0.05 | 0.00 | 0.00 | 1.03 | 0.00 | 0.38 | 1.00 | 1062.00 |
| | | | Cubic | 0.05 | 0.00 | 0.00 | 1.03 | 0.00 | 0.54 | 1.00 | 1061.00 |
| | | Moods and Feelings | Linear | 0.04 | 0.00 | 0.00 | 1.08 | 0.00 | 1.60 | 1.00 | 1063.00 |
| | | | Quadratic | 0.04 | 0.00 | 0.00 | 1.08 | 0.00 | 0.00 | 1.00 | 1062.00 |
| | | | Cubic | 0.04 | 0.00 | 0.00 | 1.08 | 0.00 | 0.01 | 1.00 | 1061.00 |
| | | ADHD | Linear | 0.05 | 0.00 | 0.00 | 1.00 | 0.00 | 0.89 | 1.00 | 440.00 |
| | | | Quadratic | 0.05 | 0.00 | 0.00 | 1.00 | 0.00 | 0.17 | 1.00 | 439.00 |
| | | | Cubic | 0.05 | 0.00 | 0.00 | 1.00 | 0.00 | 0.09 | 1.00 | 438.00 |
| | | Callous unemotional traits | Linear | 0.01 | 0.00 | 0.00 | 0.84 | 0.00 | 0.03 | 1.00 | 437.00 |
| | | | Quadratic | 0.01 | 0.00 | 0.00 | 0.84 | 0.00 | 0.01 | 1.00 | 436.00 |
| | | | Cubic | 0.06 | 0.00 | 0.00 | 0.84 | 0.00 | 1.27 | 1.00 | 435.00 |
| | | Autism | Linear | 0.00 | 0.00 | 0.00 | 0.98 | 0.00 | 0.00 | 1.00 | 1061.00 |
| | | | Quadratic | 0.02 | 0.00 | 0.00 | 0.98 | 0.00 | 0.27 | 1.00 | 1060.00 |
| | | | Cubic | 0.02 | 0.00 | 0.00 | 0.98 | 0.00 | 0.03 | 1.00 | 1059.00 |
| | | Anomalous Perceptions | Linear | 0.04 | 0.00 | 0.00 | 1.02 | 0.00 | 1.97 | 1.00 | 1062.00 |
| | | | Quadratic | 0.04 | 0.00 | 0.00 | 1.02 | 0.00 | 0.05 | 1.00 | 1061.00 |
| | | | Cubic | 0.05 | 0.00 | 0.00 | 1.02 | 0.00 | 0.47 | 1.00 | 1060.00 |
| | | Grandiosity & Delusion | Linear | 0.02 | 0.00 | 0.00 | 0.93 | 0.00 | 0.42 | 1.00 | 1063.00 |
| | | | Quadratic | 0.06 | 0.00 | 0.00 | 0.93 | 0.00 | 3.07 | 1.00 | 1062.00 |

| | | | | | | | | | | | |
|---|---|---|---|---|---|---|---|---|---|---|---|
| | | | Cubic | 0.06 | 0.00 | 0.00 | 0.93 | 0.00 | 0.09 | 1.00 | 1061.00 |
| | | Cognitive disorganisation | Linear | 0.04 | 0.00 | 0.00 | 0.98 | 0.00 | 1.65 | 1.00 | 1060.00 |
| | | | Quadratic | 0.04 | 0.00 | 0.00 | 0.98 | 0.00 | 0.13 | 1.00 | 1059.00 |
| | | | Cubic | 0.05 | 0.00 | 0.00 | 0.98 | 0.00 | 0.52 | 1.00 | 1058.00 |
| | | Hedonia | Linear | 0.02 | 0.00 | 0.00 | 0.95 | 0.00 | 0.37 | 1.00 | 1064.00 |
| | | | Quadratic | 0.04 | 0.00 | 0.00 | 0.95 | 0.00 | 0.96 | 1.00 | 1063.00 |
| | | | Cubic | 0.06 | 0.00 | 0.00 | 0.95 | 0.00 | 2.07 | 1.00 | 1062.00 |
| | | Introvertive Anhedonia | Linear | 0.07 | 0.01 | 0.00 | 0.94 | 0.01 | 5.81* | 1.00 | 1061.00 |
| | | | Quadratic | 0.07 | 0.01 | 0.00 | 0.94 | 0.00 | 0.08 | 1.00 | 1060.00 |
| | | | Cubic | 0.07 | 0.01 | 0.00 | 0.94 | 0.00 | 0.00 | 1.00 | 1059.00 |
| | | Eating problems | Linear | 0.07 | 0.00 | 0.00 | 1.02 | 0.00 | 2.00 | 1.00 | 437.00 |
| | | | Quadratic | 0.10 | 0.01 | 0.01 | 1.02 | 0.01 | 2.47 | 1.00 | 436.00 |
| | | | Cubic | 0.10 | 0.01 | 0.00 | 1.02 | 0.00 | 0.03 | 1.00 | 435.00 |
| | | Sleep problems | Linear | 0.03 | 0.00 | 0.00 | 1.03 | 0.00 | 0.46 | 1.00 | 623.00 |
| | | | Quadratic | 0.08 | 0.01 | 0.00 | 1.03 | 0.00 | 3.05 | 1.00 | 622.00 |
| | | | Cubic | 0.09 | 0.01 | 0.00 | 1.03 | 0.00 | 1.79 | 1.00 | 621.00 |
| | | Victimisation | Linear | 0.03 | 0.00 | 0.00 | 0.97 | 0.00 | 1.50 | 1.00 | 1303.00 |
| | | | Quadratic | 0.03 | 0.00 | 0.00 | 0.97 | 0.00 | 0.05 | 1.00 | 1302.00 |
| | | | Cubic | 0.03 | 0.00 | 0.00 | 0.97 | 0.00 | 0.00 | 1.00 | 1301.00 |
| | | Substance use | Linear | 0.01 | 0.00 | 0.00 | 0.93 | 0.00 | 0.01 | 1.00 | 622.00 |
| | | | Quadratic | 0.01 | 0.00 | 0.00 | 0.93 | 0.00 | 0.00 | 1.00 | 621.00 |
| | | | Cubic | 0.08 | 0.01 | 0.00 | 0.92 | 0.01 | 3.75 | 1.00 | 620.00 |
| | | Delinquency | Linear | 0.00 | 0.00 | 0.00 | 0.93 | 0.00 | 0.00 | 1.00 | 440.00 |
| | | | Quadratic | 0.00 | 0.00 | 0.00 | 0.93 | 0.00 | 0.01 | 1.00 | 439.00 |
| | | | Cubic | 0.01 | 0.00 | -0.01 | 0.93 | 0.00 | 0.04 | 1.00 | 438.00 |
| | Parent | Behavior Problems | Linear | 0.03 | 0.00 | 0.00 | 0.80 | 0.00 | 0.83 | 1.00 | 1059.00 |
| | | | Quadratic | 0.03 | 0.00 | 0.00 | 0.80 | 0.00 | 0.08 | 1.00 | 1058.00 |

| | | | | | | | | | | | |
|---|---|---|---|---|---|---|---|---|---|---|---|
| | | | Cubic | 0.05 | 0.00 | 0.00 | 0.80 | 0.00 | 1.20 | 1.00 | 1057.00 |
| | | Anxiety | Linear | 0.03 | 0.00 | 0.00 | 0.97 | 0.00 | 0.80 | 1.00 | 1059.00 |
| | | | Quadratic | 0.03 | 0.00 | 0.00 | 0.97 | 0.00 | 0.10 | 1.00 | 1058.00 |
| | | | Cubic | 0.04 | 0.00 | 0.00 | 0.97 | 0.00 | 0.51 | 1.00 | 1057.00 |
| | | Moods and Feelings | Linear | 0.01 | 0.00 | 0.00 | 0.98 | 0.00 | 0.15 | 1.00 | 1058.00 |
| | | | Quadratic | 0.02 | 0.00 | 0.00 | 0.98 | 0.00 | 0.47 | 1.00 | 1057.00 |
| | | | Cubic | 0.02 | 0.00 | 0.00 | 0.98 | 0.00 | 0.01 | 1.00 | 1056.00 |
| | | ADHD | Linear | 0.05 | 0.00 | 0.00 | 0.74 | 0.00 | 2.96 | 1.00 | 1058.00 |
| | | | Quadratic | 0.05 | 0.00 | 0.00 | 0.74 | 0.00 | 0.03 | 1.00 | 1057.00 |
| | | | Cubic | 0.05 | 0.00 | 0.00 | 0.74 | 0.00 | 0.09 | 1.00 | 1056.00 |
| | | Negative Symptoms | Linear | 0.00 | 0.00 | 0.00 | 0.78 | 0.00 | 0.02 | 1.00 | 1059.00 |
| | | | Quadratic | 0.02 | 0.00 | 0.00 | 0.79 | 0.00 | 0.36 | 1.00 | 1058.00 |
| | | | Cubic | 0.02 | 0.00 | 0.00 | 0.79 | 0.00 | 0.05 | 1.00 | 1057.00 |
| | | Callous unemotional traits | Linear | 0.07 | 0.00 | 0.00 | 0.85 | 0.00 | 4.83* | 1.00 | 1058.00 |
| | | | Quadratic | 0.07 | 0.01 | 0.00 | 0.85 | 0.00 | 0.65 | 1.00 | 1057.00 |
| | | | Cubic | 0.08 | 0.01 | 0.00 | 0.85 | 0.00 | 1.18 | 1.00 | 1056.00 |
| | | Autism | Linear | 0.01 | 0.00 | 0.00 | 0.87 | 0.00 | 0.22 | 1.00 | 1057.00 |
| | | | Quadratic | 0.03 | 0.00 | 0.00 | 0.87 | 0.00 | 0.59 | 1.00 | 1056.00 |
| | | | Cubic | 0.03 | 0.00 | 0.00 | 0.87 | 0.00 | 0.35 | 1.00 | 1055.00 |

Note: $R^2$= proportion of variance explained; Adj $R^2$ = adjusted $R^2$ in new model. Significant non-linear results highlighted in grey. *= p<.05; **= p<.01

***Table S4.6*** - Linear, quadratic and cubic associations between boys' age 16 PDS score and behavioral and psychiatric problems concurrently and over time. Analysis was conducted by randomly selecting one twin per pair.

| | | | Model | R | $R^2$ | Adj $R^2$ | Std. Error | Change statistics | | | |
|---|---|---|---|---|---|---|---|---|---|---|---|
| | | | | | | | | $R^2$ Change | F Change | df1 | df2 |
| Age 16 | Self | Behavior Problems | Linear | 0.07 | 0.01 | 0.00 | 0.95 | 0.01 | 3.99* | 1.00 | 719.00 |
| | | | Quadratic | 0.09 | 0.01 | 0.01 | 0.95 | 0.00 | 2.09 | 1.00 | 718.00 |
| | | | Cubic | 0.09 | 0.01 | 0.00 | 0.95 | 0.00 | 0.21 | 1.00 | 717.00 |
| | | Anxiety | Linear | 0.05 | 0.00 | 0.00 | 0.76 | 0.00 | 1.70 | 1.00 | 720.00 |
| | | | Quadratic | 0.05 | 0.00 | 0.00 | 0.76 | 0.00 | 0.06 | 1.00 | 719.00 |
| | | | Cubic | 0.05 | 0.00 | 0.00 | 0.76 | 0.00 | 0.09 | 1.00 | 718.00 |
| | | Moods and Feelings | Linear | 0.05 | 0.00 | 0.00 | 0.84 | 0.00 | 1.68 | 1.00 | 719.00 |
| | | | Quadratic | 0.05 | 0.00 | 0.00 | 0.84 | 0.00 | 0.14 | 1.00 | 718.00 |
| | | | Cubic | 0.05 | 0.00 | 0.00 | 0.84 | 0.00 | 0.01 | 1.00 | 717.00 |
| | | ADHD | Linear | 0.00 | 0.00 | 0.00 | 0.96 | 0.00 | 0.00 | 1.00 | 272.00 |
| | | | Quadratic | 0.03 | 0.00 | -0.01 | 0.96 | 0.00 | 0.27 | 1.00 | 271.00 |
| | | | Cubic | 0.09 | 0.01 | 0.00 | 0.96 | 0.01 | 1.83 | 1.00 | 270.00 |
| | | Callous unemotional traits | Linear | 0.11 | 0.01 | 0.01 | 1.02 | 0.01 | 3.31 | 1.00 | 272.00 |
| | | | Quadratic | 0.24 | 0.06 | 0.05 | 1.00 | 0.04 | 12.63** | 1.00 | 271.00 |
| | | | Cubic | 0.25 | 0.06 | 0.05 | 1.00 | 0.00 | 1.22 | 1.00 | 270.00 |
| | | Autism | Linear | 0.06 | 0.00 | 0.00 | 0.98 | 0.00 | 2.28 | 1.00 | 719.00 |
| | | | Quadratic | 0.08 | 0.01 | 0.00 | 0.98 | 0.00 | 2.85 | 1.00 | 718.00 |
| | | | Cubic | 0.10 | 0.01 | 0.01 | 0.98 | 0.00 | 1.49 | 1.00 | 717.00 |
| | | Anomalous Perceptions | Linear | 0.03 | 0.00 | 0.00 | 0.97 | 0.00 | 0.48 | 1.00 | 720.00 |
| | | | Quadratic | 0.03 | 0.00 | 0.00 | 0.97 | 0.00 | 0.01 | 1.00 | 719.00 |
| | | | Cubic | 0.04 | 0.00 | 0.00 | 0.97 | 0.00 | 0.49 | 1.00 | 718.00 |
| | | Grandiosity & Delusion | Linear | 0.09 | 0.01 | 0.01 | 1.03 | 0.01 | 5.90* | 1.00 | 720.00 |
| | | | Quadratic | 0.12 | 0.01 | 0.01 | 1.02 | 0.01 | 4.61* | 1.00 | 719.00 |

| | | | | | | | | | | | |
|---|---|---|---|---|---|---|---|---|---|---|---|
| | | | Cubic | 0.12 | 0.01 | 0.01 | 1.02 | 0.00 | 0.18 | 1.00 | 718.00 |
| | | Cognitive disorganisation | Linear | 0.01 | 0.00 | 0.00 | 0.94 | 0.00 | 0.09 | 1.00 | 719.00 |
| | | | Quadratic | 0.01 | 0.00 | 0.00 | 0.94 | 0.00 | 0.03 | 1.00 | 718.00 |
| | | | Cubic | 0.04 | 0.00 | 0.00 | 0.94 | 0.00 | 1.27 | 1.00 | 717.00 |
| | | Hedonia | Linear | 0.02 | 0.00 | 0.00 | 0.99 | 0.00 | 0.28 | 1.00 | 718.00 |
| | | | Quadratic | 0.10 | 0.01 | 0.01 | 0.99 | 0.01 | 6.69* | 1.00 | 717.00 |
| | | | Cubic | 0.11 | 0.01 | 0.01 | 0.99 | 0.00 | 1.23 | 1.00 | 716.00 |
| | | Introvertive Anhedonia | Linear | 0.06 | 0.00 | 0.00 | 0.98 | 0.00 | 2.55 | 1.00 | 719.00 |
| | | | Quadratic | 0.09 | 0.01 | 0.01 | 0.98 | 0.01 | 3.66 | 1.00 | 718.00 |
| | | | Cubic | 0.09 | 0.01 | 0.00 | 0.98 | 0.00 | 0.14 | 1.00 | 717.00 |
| | | Eating problems | Linear | 0.04 | 0.00 | 0.00 | 0.73 | 0.00 | 0.54 | 1.00 | 273.00 |
| | | | Quadratic | 0.05 | 0.00 | 0.00 | 0.73 | 0.00 | 0.11 | 1.00 | 272.00 |
| | | | Cubic | 0.05 | 0.00 | -0.01 | 0.73 | 0.00 | 0.03 | 1.00 | 271.00 |
| | | Sleep problems | Linear | 0.11 | 0.01 | 0.01 | 0.94 | 0.01 | 5.58* | 1.00 | 445.00 |
| | | | Quadratic | 0.11 | 0.01 | 0.01 | 0.94 | 0.00 | 0.10 | 1.00 | 444.00 |
| | | | Cubic | 0.12 | 0.01 | 0.01 | 0.94 | 0.00 | 0.58 | 1.00 | 443.00 |
| | | Victimisation | Linear | 0.01 | 0.00 | 0.00 | 1.02 | 0.00 | 0.16 | 1.00 | 910.00 |
| | | | Quadratic | 0.04 | 0.00 | 0.00 | 1.02 | 0.00 | 1.14 | 1.00 | 909.00 |
| | | | Cubic | 0.04 | 0.00 | 0.00 | 1.02 | 0.00 | 0.07 | 1.00 | 908.00 |
| | | Substance use | Linear | 0.01 | 0.00 | 0.00 | 1.02 | 0.00 | 0.06 | 1.00 | 445.00 |
| | | | Quadratic | 0.07 | 0.00 | 0.00 | 1.02 | 0.00 | 2.02 | 1.00 | 444.00 |
| | | | Cubic | 0.09 | 0.01 | 0.00 | 1.02 | 0.00 | 1.18 | 1.00 | 443.00 |
| | | Delinquency | Linear | 0.05 | 0.00 | 0.00 | 1.01 | 0.00 | 0.61 | 1.00 | 273.00 |
| | | | Quadratic | 0.05 | 0.00 | 0.00 | 1.01 | 0.00 | 0.16 | 1.00 | 272.00 |
| | | | Cubic | 0.06 | 0.00 | -0.01 | 1.02 | 0.00 | 0.06 | 1.00 | 271.00 |
| | Parent | Behavior Problems | Linear | 0.10 | 0.01 | 0.01 | 0.90 | 0.01 | 7.05** | 1.00 | 722.00 |
| | | | Quadratic | 0.12 | 0.01 | 0.01 | 0.90 | 0.00 | 3.55 | 1.00 | 721.00 |

| | | | | | | | | | |
|---|---|---|---|---|---|---|---|---|---|
| | Cubic | 0.14 | 0.02 | 0.01 | 0.90 | 0.00 | 2.85 | 1.00 | 720.00 |
| Anxiety | Linear | 0.08 | 0.01 | 0.01 | 0.78 | 0.01 | 4.70* | 1.00 | 721.00 |
| | Quadratic | 0.08 | 0.01 | 0.00 | 0.78 | 0.00 | 0.42 | 1.00 | 720.00 |
| | Cubic | 0.10 | 0.01 | 0.01 | 0.78 | 0.00 | 1.81 | 1.00 | 719.00 |
| Moods and Feelings | Linear | 0.05 | 0.00 | 0.00 | 0.82 | 0.00 | 1.92 | 1.00 | 721.00 |
| | Quadratic | 0.07 | 0.00 | 0.00 | 0.82 | 0.00 | 1.45 | 1.00 | 720.00 |
| | Cubic | 0.07 | 0.00 | 0.00 | 0.83 | 0.00 | 0.00 | 1.00 | 719.00 |
| ADHD | Linear | 0.08 | 0.01 | 0.00 | 0.94 | 0.01 | 4.53* | 1.00 | 722.00 |
| | Quadratic | 0.10 | 0.01 | 0.01 | 0.94 | 0.00 | 3.14 | 1.00 | 721.00 |
| | Cubic | 0.14 | 0.02 | 0.01 | 0.94 | 0.01 | 5.84** | 1.00 | 720.00 |
| Negative Symptoms | Linear | 0.03 | 0.00 | 0.00 | 1.02 | 0.00 | 0.56 | 1.00 | 721.00 |
| | Quadratic | 0.05 | 0.00 | 0.00 | 1.02 | 0.00 | 0.94 | 1.00 | 720.00 |
| | Cubic | 0.05 | 0.00 | 0.00 | 1.02 | 0.00 | 0.51 | 1.00 | 719.00 |
| Callous unemotional traits | Linear | 0.10 | 0.01 | 0.01 | 0.93 | 0.01 | 7.43* | 1.00 | 721.00 |
| | Quadratic | 0.12 | 0.01 | 0.01 | 0.93 | 0.00 | 2.17 | 1.00 | 720.00 |
| | Cubic | 0.12 | 0.02 | 0.01 | 0.93 | 0.00 | 1.69 | 1.00 | 719.00 |
| Autism | Linear | 0.08 | 0.01 | 0.00 | 1.01 | 0.01 | 4.34* | 1.00 | 722.00 |
| | Quadratic | 0.08 | 0.01 | 0.00 | 1.02 | 0.00 | 0.24 | 1.00 | 721.00 |
| | Cubic | 0.08 | 0.01 | 0.00 | 1.02 | 0.00 | 0.27 | 1.00 | 720.00 |

Note: Note: $R^2$= proportion of variance explained; Adj $R^2$ = adjusted $R^2$ in new model. Significant non-linear results highlighted in grey. *= p<.05; **= p<.01

***Table S5.1*** - Analysis of variance and associated post-hoc comparisons looking at early, on-time and late pubertal timing in girls at age 11 and behavioral and psychiatric problems concurrently and over time. Analysis was conducted by randomly selecting one twin per pair.

| | | Measure | Early | | | On-time | | | Late | | | $F$ | Early vs. On time | | | On time vs. Late | | |
|---|---|---|---|---|---|---|---|---|---|---|---|---|---|---|---|---|---|---|
| | | | $N$ | $M$ | $SD$ | $N$ | $M$ | $SD$ | $N$ | $M$ | $SD$ | | $t$ | $d$ | $R^2$ | $t$ | $d$ | $R^2$ |
| Age 11 | Self-report | Behavior Problems | 456 | 8.75 | 5.20 | 1768 | 8.24 | 5.17 | 463 | 7.96 | 5.24 | 3.18* | -2.09* | 0.08 | 0.04 | | | |
| | | Moods and Feelings | 459 | 3.46 | 4.08 | 1777 | 3.18 | 3.97 | 467 | 2.78 | 3.65 | 3.76* | | | | | | |
| | | Vicitmisation | 458 | 7.35 | 6.97 | 1776 | 6.84 | 6.75 | 467 | 6.39 | 6.64 | 3.40* | -2.14* | -0.08 | 0.04 | | | |
| | Parent-report | Behavior Problems | 456 | 6.65 | 4.97 | 1762 | 6.18 | 4.54 | 465 | 6.01 | 4.82 | 3.04* | -2.23 | -0.09 | 0.04 | | | |
| | | Moods and Feelings | 456 | 1.86 | 3.15 | 1763 | 1.63 | 2.74 | 464 | 1.50 | 2.73 | 2.32 | | | | | | |
| | | Antisocial Personality | 457 | 6.68 | 4.45 | 1771 | 6.42 | 3.96 | 465 | 6.19 | 3.83 | 1.84 | | | | | | |
| | | Autism | 457 | 4.46 | 2.94 | 1767 | 4.15 | 2.93 | 465 | 4.21 | 2.95 | 2.39 | | | | | | |
| | | ADHD | 457 | 8.06 | 6.93 | 1768 | 7.93 | 7.30 | 464 | 7.43 | 7.03 | 1.30 | | | | | | |
| Age 14 | Self-report | Victimisation | 248 | 7.89 | 7.36 | 1011 | 6.68 | 6.34 | 248 | 5.65 | 6.03 | 7.84** | -2.51* | -0.14 | 0.07 | 2.35* | 0.11 | 0.06 |
| | | ADHD | 249 | 12.84 | 7.52 | 1015 | 12.66 | 7.68 | 248 | 11.81 | 7.20 | 1.47 | | | | | | |
| | Parent-report | Victimisation | 224 | 7.37 | 7.52 | 899 | 5.68 | 7.10 | 222 | 5.49 | 6.93 | 6.01** | -3.32** | -0.18 | 0.09 | | | |
| | | Autism | 243 | 35.55 | 11.84 | 978 | 34.97 | 11.25 | 245 | 36.87 | 12.08 | 2.81 | | | | | | |
| | | Antisocial Personality | 245 | 7.21 | 4.76 | 985 | 6.95 | 4.80 | 245 | 6.50 | 4.35 | 1.60 | | | | | | |
| | | ADHD | 247 | 6.98 | 7.06 | 990 | 6.63 | 6.79 | 246 | 6.61 | 6.53 | 0.52 | | | | | | |
| Age 16 | Self-report | Behavior Problems | 342 | 10.32 | 5.16 | 1434 | 9.83 | 5.17 | 374 | 9.23 | 5.02 | 4.08* | | | | -1.98 | 0.09 | 0.04 |
| | | Anxiety | 343 | 10.00 | 6.33 | 1435 | 9.46 | 6.12 | 374 | 8.88 | 6.36 | 2.91 | | | | | | |
| | | Moods and Feelings | 343 | 4.98 | 5.53 | 1435 | 4.31 | 4.86 | 374 | 3.73 | 4.36 | 5.74** | | | | 2.32 | 0.18 | 0.09 |
| | | ADHD | 81 | 4.89 | .95 | 346 | 4.73 | .86 | 95 | 4.86 | .84 | 1.73 | | | | | | |
| | | Callous unemotional traits | 80 | 17.67 | 7.56 | 345 | 17.82 | 6.56 | 95 | 16.68 | 6.35 | 1.06 | | | | | | |
| | | Autism | 343 | 11.91 | 5.98 | 1435 | 11.48 | 5.85 | 374 | 11.61 | 5.47 | 0.84 | | | | | | |

| | | | | | | | | | | | | | | | | | | |
|---|---|---|---|---|---|---|---|---|---|---|---|---|---|---|---|---|---|---|
| | | Paranoid Checklist | 343 | 14.34 | 11.09 | 1434 | 12.53 | 10.83 | 373 | 10.41 | 9.22 | 12.33** | -2.69 | -0.24 | 0.11 | 3.83 | 0.30 | 0.11 |
| | | Anomalous Perceptions | 343 | 5.87 | 7.04 | 1435 | 4.85 | 6.00 | 373 | 3.93 | 5.51 | 8.97** | -2.48 | 0.23 | 0.11 | 2.80 | 0.22 | 0.11 |
| | | Grandiosity & Delusion | 343 | 5.51 | 4.51 | 1434 | 4.86 | 4.28 | 373 | 4.34 | 3.91 | 6.58** | -2.33 | -0.21 | 0.10 | 2.29 | 0.18 | 0.09 |
| | | Cognitive Disorganisation | 343 | 4.53 | 2.86 | 1432 | 4.35 | 2.84 | 374 | 4.07 | 2.82 | 2.36 | | | | | | |
| | | Hedonia | 343 | 34.81 | 8.11 | 1436 | 35.70 | 7.22 | 374 | 35.10 | 7.51 | 2.75 | | | | | | |
| | | Introvertive Anhedonia | 343 | 1.14 | 1.25 | 1432 | 1.12 | 1.30 | 374 | 1.10 | 1.22 | 0.15 | | | | | | |
| | | Eating problems | 80 | 4.28 | 1.97 | 344 | 3.97 | 1.94 | 94 | 3.13 | 1.78 | 9.45** | | | | 4.01 | 0.64 | 0.30 |
| | | Peer Victimisation | 221 | 3.29 | 3.35 | 761 | 3.08 | 3.11 | 207 | 2.80 | 2.99 | 1.30 | | | | | | |
| | | Sleep | 262 | 9.34 | 5.45 | 1090 | 8.32 | 4.87 | 280 | 7.75 | 4.75 | 6.77** | -2.53 | -0.26 | 0.13 | | | |
| | | Substance use | 262 | 2.81 | 2.79 | 1091 | 2.57 | 2.67 | 278 | 2.26 | 2.49 | 2.96 | | | | | | |
| | | Delinquency | 81 | 4.99 | 6.58 | 346 | 4.84 | 6.79 | 95 | 3.34 | 5.87 | 2.17 | | | | | | |
| | Parent-report | SDQ Behavior Problems | 342 | 3.29 | 2.90 | 1439 | 3.34 | 2.91 | 372 | 3.10 | 2.81 | 0.95 | | | | | | |
| | | Anxiety | 342 | 4.46 | 4.40 | 1440 | 4.11 | 4.46 | 372 | 3.82 | 4.47 | 2.03 | | | | | | |
| | | Moods and Feelings | 342 | 1.34 | 2.58 | 1440 | 1.12 | 2.41 | 371 | .88 | 2.50 | 3.15* | NS | | | | | |
| | | ADHD | 341 | 5.14 | 6.41 | 1437 | 5.41 | 6.09 | 372 | 5.29 | 6.33 | 0.08 | | | | | | |
| | | Negative Symptoms | 342 | 2.36 | 3.23 | 1439 | 2.45 | 3.52 | 372 | 2.27 | 3.50 | 0.37 | | | | | | |
| | | Callous unemotional traits | 342 | 15.21 | 8.53 | 1439 | 15.76 | 8.71 | 372 | 15.48 | 8.12 | 0.53 | | | | | | |
| | | Autism | 342 | 21.88 | 9.59 | 1438 | 21.99 | 10.04 | 372 | 22.32 | 9.99 | 0.24 | | | | | | |

*Note: N*= sample size; *M*=Mean; *SD* = standard deviation; $R^2$ = variance explained; *O* = on-time pubertal group; *E* = early pubertal timing group; *L* = late pubertal timing group; *NS* = non-significant.

***Table S5.2*** - Analysis of variance and associated post-hoc comparisons looking at early, on-time and late pubertal timing in boys at age 11 and behavioral and psychiatric problems concurrently and over time. Analysis was conducted by randomly selecting one twin per pair.

| | | Measure | Early | | | On-time | | | Late | | | *F* | Early vs. On time | | | On time vs. Late | | |
|---|---|---|---|---|---|---|---|---|---|---|---|---|---|---|---|---|---|---|
| | | | *N* | *M* | *SD* | *N* | *M* | *SD* | *N* | *M* | *SD* | | *t* | *d* | $R^2$ | *t* | *d* | $R^2$ |
| Age 11 | Self-report | Behavior Problems | 391 | 10.29 | 5.42 | 1609 | 9.37 | 5.33 | 352 | 8.24 | 5.13 | 12.15** | -3.14** | -0.13 | 0.06 | 3.14** | 0.13 | 0.06 |
| | | Moods and Feelings | 392 | 4.14 | 3.91 | 1612 | 3.39 | 3.73 | 352 | 2.67 | 3.22 | 13.05** | -3.56** | -0.30 | 0.15 | 3.04** | 0.25 | 0.13 |
| | | Victimisation | 392 | 10.26 | 7.67 | 1610 | 8.38 | 7.64 | 352 | 6.27 | 7.05 | 20.34** | -4.64** | -0.38 | 0.19 | 3.57** | 0.31 | 0.15 |
| | Parent-report | Behavior Problems | 390 | 7.82 | 5.07 | 1597 | 7.51 | 5.05 | 349 | 7.15 | 5.23 | 1.07 | | | | | | |
| | | Moods and Feelings | 390 | 1.79 | 2.64 | 1596 | 1.86 | 2.67 | 349 | 1.75 | 2.73 | 0.08 | | | | | | |
| | | Antisocial Personality | 390 | 8.37 | 4.59 | 1599 | 7.84 | 4.45 | 349 | 7.73 | 4.68 | 2.43 | | | | | | |
| | | Autism | 391 | 5.58 | 3.46 | 1598 | 5.13 | 3.25 | 348 | 5.24 | 3.45 | 3.10* | -2.46* | -0.10 | 0.05 | | | |
| | | ADHD | 391 | 11.81 | 8.87 | 1594 | 11.28 | 8.78 | 349 | 9.84 | 8.38 | 2.90 | | | | | | |
| Age 14 | Self-report | Victimisation | 187 | 9.18 | 7.33 | 836 | 8.59 | 7.88 | 208 | 7.13 | 7.11 | 3.68* | | | | 2.38* | 0.26 | 0.13 |
| | | ADHD | 188 | 14.60 | 8.65 | 840 | 13.91 | 8.24 | 208 | 13.14 | 7.55 | 1.54 | | | | | | |
| | Parent-report | Victimisation | 167 | 7.34 | 7.82 | 752 | 6.74 | 8.00 | 183 | 5.94 | 7.41 | 1.26 | | | | | | |
| | | Autism | 192 | 39.29 | 12.57 | 868 | 37.89 | 12.80 | 209 | 38.72 | 12.87 | 1.46 | | | | | | |
| | | Antisocial Personality | 194 | 9.01 | 5.33 | 875 | 8.57 | 5.21 | 212 | 8.05 | 4.73 | 1.63 | | | | | | |
| | | ADHD | 198 | 10.28 | 8.66 | 880 | 9.79 | 8.68 | 212 | 9.19 | 8.21 | 0.69 | | | | | | |
| Age 16 | Self-report | Behavior Problems | 271 | 9.37 | 4.81 | 1178 | 8.83 | 4.93 | 256 | 8.25 | 4.63 | 3.18* | | | | | | |
| | | Anxiety | 273 | 6.71 | 4.82 | 1179 | 5.87 | 4.68 | 255 | 5.34 | 3.93 | 6.49** | -2.70** | -0.13 | 0.07 | | | |
| | | Moods and Feelings | 273 | 3.22 | 3.65 | 1178 | 2.68 | 3.65 | 256 | 2.24 | 2.83 | 6.45** | -2.12* | -0.21 | 0.10 | 2.84** | 0.26 | 0.13 |

| | | | | | | | | | | | | | | | | | | |
|---|---|---|---|---|---|---|---|---|---|---|---|---|---|---|---|---|---|---|
| | | ADHD | 43 | 4.50 | .83 | 212 | 4.78 | .80 | 92 | 4.83 | .82 | 2.60 | | | | | | |
| | | Callous unemotional traits | 42 | 22.99 | 8.55 | 212 | 21.88 | 7.83 | 92 | 21.68 | 7.77 | 0.42 | | | | | | |
| | | Autism | 273 | 12.75 | 5.43 | 1178 | 12.06 | 5.70 | 255 | 12.39 | 6.00 | 1.96 | | | | | | |
| | | Paranoid Checklist | 271 | 13.76 | 11.86 | 1178 | 11.80 | 10.28 | 256 | 10.90 | 10.01 | 5.60** | -2.49* | -0.26 | 0.13 | | | |
| | | Anomalous Perceptions | 273 | 6.50 | 7.48 | 1179 | 3.96 | 5.45 | 256 | 3.59 | 4.92 | 23.98** | -5.30** | -0.57 | 0.28 | | | |
| | | Grandiosity & Delusion | 272 | 6.23 | 4.52 | 1179 | 5.66 | 4.33 | 256 | 5.70 | 5.01 | 1.80 | | | | | | |
| | | Cognitive Disorganisation | 272 | 3.69 | 2.73 | 1177 | 3.37 | 2.73 | 255 | 3.12 | 2.54 | 3.45* | | | | | | |
| | | Hedonia | 271 | 31.84 | 7.65 | 1178 | 31.34 | 8.09 | 255 | 30.83 | 8.06 | 1.37 | | | | | | |
| | | Introvertive Anhedonia | 272 | 1.41 | 1.30 | 1178 | 1.49 | 1.31 | 255 | 1.44 | 1.35 | 0.37 | | | | | | |
| | | Eating problems | 42 | 1.98 | 1.27 | 211 | 2.26 | 1.40 | 92 | 1.90 | 1.24 | 2.35 | | | | | | |
| | | Peer Victimisation | 137 | 4.40 | 3.34 | 529 | 4.13 | 3.20 | 199 | 3.91 | 3.31 | 0.93 | | | | | | |
| | | Sleep | 230 | 8.25 | 4.69 | 966 | 7.15 | 4.39 | 164 | 6.93 | 3.80 | 7.50** | -3.07** | -0.34 | 0.17 | | | |
| | | Substance use | 230 | 2.98 | 2.96 | 967 | 2.57 | 2.84 | 164 | 2.57 | 2.85 | 4.71** | | | | 2.39* | 0.13 | 0.06 |
| | | Delinquency | 43 | 8.02 | 8.83 | 212 | 6.41 | 7.85 | 92 | 5.49 | 7.38 | 1.52 | | | | | | |
| | Parent-report | SDQ Behavior Problems | 277 | 4.36 | 3.40 | 1179 | 4.17 | 3.20 | 259 | 4.12 | 3.26 | 0.52 | | | | | | |
| | | Anxiety | 277 | 2.62 | 3.19 | 1181 | 2.63 | 3.44 | 259 | 2.51 | 3.55 | 0.08 | | | | | | |
| | | Moods and Feelings | 277 | .76 | 1.74 | 1179 | .73 | 1.84 | 259 | .70 | 1.72 | 0.05 | | | | | | |
| | | ADHD | 276 | 8.48 | 8.75 | 1182 | 7.72 | 7.61 | 259 | 6.64 | 7.19 | 2.17 | | | | | | |
| | | Negative Symptoms | 276 | 3.42 | 4.32 | 1178 | 2.98 | 3.78 | 259 | 3.31 | 4.58 | 2.34 | | | | | | |
| | | Callous unemotional traits | 277 | 20.26 | 9.98 | 1180 | 19.59 | 9.14 | 258 | 19.62 | 9.02 | 0.65 | | | | | | |
| | | Autism | 277 | 25.95 | 11.07 | 1180 | 25.70 | 10.95 | 258 | 26.21 | 11.41 | 0.99 | | | | | | |

*Note: N*= sample size; *M*=Mean; *SD* = standard deviation; $R^2$ = variance explained; *O* = on-time pubertal group; *E* = early pubertal timing group; *L* = late pubertal timing group; *NS* = non-significant.

***Table S5.3*** - Analysis of variance and associated post-hoc comparisons looking at early, on-time and late pubertal timing in girls at age 14 and behavioral and psychiatric problems concurrently and at age 16. Analysis was conducted by randomly selecting one twin per pair.

No significant differences between early, on time and late groups

| | | Measure | Early | | | On-time | | | Late | | | *F* |
|---|---|---|---|---|---|---|---|---|---|---|---|---|
| | | | *N* | *M* | *SD* | *N* | *M* | *SD* | *N* | *M* | *SD* | |
| Age 14 | Self-report | Victimisation | 208 | 7.13 | 7.11 | 1139 | 6.73 | 6.47 | 249 | 6.79 | 6.47 | 0.03 |
| | | ADHD | 208 | 13.14 | 7.55 | 1140 | 12.75 | 7.88 | 249 | 12.13 | 6.77 | 0.74 |
| | Parent-report | Victimisation | 183 | 5.94 | 7.41 | 954 | 5.95 | 7.15 | 209 | 6.44 | 7.58 | 0.19 |
| | | Autism | 209 | 38.72 | 12.87 | 1045 | 35.27 | 10.86 | 225 | 36.46 | 12.61 | 0.69 |
| | | Antisocial Personality | 212 | 8.05 | 4.73 | 1052 | 6.94 | 4.52 | 225 | 6.68 | 4.65 | 0.61 |
| | | ADHD | 212 | 9.19 | 8.21 | 1057 | 6.59 | 6.58 | 225 | 7.13 | 7.43 | 0.33 |
| Age 16 | Self-report | Behavior Problems | 256 | 8.25 | 4.63 | 973 | 9.47 | 5.12 | 213 | 9.69 | 4.67 | 0.30 |
| | | Anxiety | 255 | 5.34 | 3.93 | 976 | 9.39 | 6.25 | 213 | 8.84 | 5.83 | 0.71 |
| | | Moods and Feelings | 256 | 2.24 | 2.83 | 975 | 4.13 | 4.74 | 213 | 4.18 | 4.38 | 1.76 |
| | | ADHD | 92 | 4.83 | .82 | 341 | 4.77 | .88 | 79 | 4.85 | .76 | 0.47 |
| | | Callous unemotional traits | 92 | 21.68 | 7.77 | 339 | 17.88 | 6.55 | 79 | 16.77 | 5.82 | 1.16 |
| | | Autism | 255 | 12.39 | 6.00 | 974 | 11.40 | 5.87 | 213 | 11.95 | 5.82 | 1.38 |
| | | Paranoid Checklist | 256 | 10.90 | 10.01 | 975 | 12.58 | 10.84 | 213 | 11.74 | 10.55 | 1.06 |
| | | Anomalous Perceptions | 256 | 3.59 | 4.92 | 975 | 4.74 | 6.05 | 212 | 4.31 | 5.91 | 2.34 |
| | | Grandiosity & Delusion | 256 | 5.70 | 5.01 | 975 | 4.76 | 4.05 | 212 | 4.16 | 4.03 | 2.58 |
| | | Cognitive Disorganisation | 255 | 3.12 | 2.54 | 974 | 4.31 | 2.86 | 213 | 4.21 | 2.66 | 0.29 |
| | | Hedonia | 255 | 30.83 | 8.06 | 976 | 35.60 | 7.19 | 213 | 35.14 | 6.84 | 0.66 |

| | | | | | | | | | | | |
|---|---|---|---|---|---|---|---|---|---|---|---|
| | | Introvertive Anhedonia | 255 | 1.44 | 1.35 | 974 | 1.08 | 1.25 | 213 | 1.06 | 1.27 | 0.15 |
| | | Eating problems | 92 | 1.90 | 1.24 | 338 | 3.89 | 1.92 | 78 | 3.57 | 1.92 | 0.99 |
| | | Peer Victimisation | 199 | 3.91 | 3.31 | 673 | 3.06 | 3.14 | 138 | 3.05 | 3.17 | 1.06 |
| | | Sleep | 164 | 6.93 | 3.80 | 636 | 8.34 | 4.86 | 134 | 8.45 | 5.25 | 1.19 |
| | | Substance use | 164 | 2.57 | 2.85 | 635 | 2.60 | 2.49 | 134 | 2.17 | 2.32 | 1.19 |
| | | Delinquency | 92 | 5.49 | 7.38 | 340 | 4.13 | 6.19 | 79 | 3.89 | 6.35 | 2.38 |
| | Parent-report | SDQ Behavior Problems | 259 | 4.12 | 3.26 | 970 | 3.10 | 2.77 | 213 | 3.25 | 2.80 | 0.22 |
| | | Behavior Problems | 259 | 2.51 | 3.55 | 972 | 3.78 | 4.09 | 213 | 4.32 | 4.28 | 1.43 |
| | | Anxiety | 259 | .70 | 1.72 | 972 | 1.03 | 2.33 | 212 | .88 | 1.92 | 0.55 |
| | | Moods and Feelings | 259 | 6.64 | 7.19 | 968 | 4.93 | 5.58 | 213 | 5.12 | 6.09 | 0.01 |
| | | ADHD | 259 | 3.31 | 4.58 | 972 | 2.20 | 3.28 | 213 | 2.36 | 3.27 | .14 |
| | | Negative Symptoms | 258 | 19.62 | 9.02 | 971 | 15.60 | 8.25 | 213 | 16.05 | 8.41 | 1.68 |
| | | Callous unemotional traits | 258 | 26.21 | 11.41 | 970 | 21.53 | 9.93 | 213 | 21.79 | 10.50 | 0.02 |

*Note: N*= sample size; *M*=Mean; *SD* = standard deviation. No significant ANOVA results

***Table S5.4*** - Analysis of variance and associated post-hoc comparisons looking at early, on-time and late pubertal timing in boys at age 14 and behavioral and psychiatric problems concurrently and at age 16. Analysis was conducted by randomly selecting one twin per pair.

| | | Measure | Early | | | On-time | | | Late | | | *F* | Early vs. On time | | | On time vs. Late | | |
|---|---|---|---|---|---|---|---|---|---|---|---|---|---|---|---|---|---|---|
| | | | *N* | *M* | *SD* | *N* | *M* | *SD* | *N* | *M* | *SD* | | t | *d* | $R^2$ | t | d | $R^2$ |
| Age 14 | Self-report | Victimisation | 186 | 7.35 | 6.70 | 901 | 8.77 | 7.85 | 229 | 8.69 | 8.28 | 2.88 | | | | | | |
| | | ADHD | 186 | 13.59 | 8.77 | 902 | 13.85 | 8.06 | 227 | 15.09 | 9.07 | 2.25 | | | | | | |
| | Parent-report | Victimisation | 158 | 5.59 | 7.01 | 739 | 6.91 | 8.03 | 187 | 6.53 | 7.67 | 2.35 | | | | | | |
| | | Autism | 180 | 36.44 | 12.73 | 854 | 38.35 | 12.57 | 208 | 39.02 | 12.99 | 2.38 | | | | | | |
| | | Antisocial Personality | 181 | 8.21 | 5.57 | 860 | 8.64 | 5.10 | 211 | 8.55 | 5.11 | 0.65 | | | | | | |
| | | ADHD | 181 | 8.42 | 7.82 | 864 | 9.84 | 8.47 | 213 | 10.74 | 9.40 | 3.78* | 2.26* | 0.13 | 0.06 | | | |
| Age 16 | Self-report | Behavior Problems | 147 | 8.29 | 4.64 | 723 | 8.71 | 4.93 | 178 | 8.47 | 4.65 | 0.54 | | | | | | |
| | | Anxiety | 148 | 5.94 | 4.30 | 724 | 5.77 | 4.42 | 177 | 6.22 | 4.69 | 0.78 | | | | | | |
| | | Moods and Feelings | 148 | 2.83 | 3.62 | 724 | 2.72 | 3.62 | 177 | 2.44 | 3.43 | 0.50 | | | | | | |
| | | ADHD | 62 | 4.79 | .97 | 200 | 4.70 | .75 | 55 | 4.84 | .91 | 0.66 | | | | | | |
| | | Callous unemotional traits | 62 | 21.62 | 7.99 | 199 | 22.74 | 7.73 | 55 | 20.45 | 7.61 | 2.05 | | | | | | |
| | | Autism | 148 | 12.27 | 5.77 | 723 | 12.28 | 5.73 | 178 | 12.26 | 5.78 | 0.00 | | | | | | |
| | | Paranoid Checklist | 147 | 12.17 | 9.44 | 724 | 11.96 | 10.58 | 177 | 12.03 | 11.72 | 0.03 | | | | | | |
| | | Anomalous Perceptions | 148 | 4.45 | 5.73 | 724 | 4.29 | 5.91 | 178 | 4.02 | 5.83 | 0.23 | | | | | | |
| | | Grandiosity & Delusion | 148 | 6.33 | 5.02 | 724 | 5.66 | 4.37 | 177 | 5.97 | 4.78 | 1.46 | | | | | | |
| | | Cognitive Disorganisation | 148 | 3.23 | 2.65 | 723 | 3.26 | 2.61 | 178 | 3.14 | 2.56 | 0.11 | | | | | | |
| | | Hedonia | 147 | 31.11 | 7.69 | 723 | 31.28 | 7.50 | 177 | 32.28 | 8.49 | 1.43 | | | | | | |
| | | Introvertive Anhedonia | 147 | 1.44 | 1.33 | 723 | 1.36 | 1.30 | 178 | 1.34 | 1.24 | 0.34 | | | | | | |
| | | Eating problems | 61 | 2.28 | 1.41 | 200 | 2.21 | 1.45 | 55 | 1.92 | 1.29 | 1.09 | | | | | | |

| | | | | | | | | | | | | | | | | | | |
|---|---|---|---|---|---|---|---|---|---|---|---|---|---|---|---|---|---|---|
| | | Peer Victimisation | 97 | 3.68 | 2.90 | 482 | 4.18 | 3.16 | 111 | 4.31 | 3.63 | 1.15 | | | | | | |
| | | Sleep | 86 | 7.27 | 4.08 | 523 | 7.41 | 4.38 | 123 | 6.10 | 3.76 | 4.13* | 2.86** | 0.21 | 0.10 | | | |
| | | Substance use | 86 | 2.33 | 2.74 | 524 | 2.82 | 2.79 | 123 | 2.56 | 2.87 | 0.76 | | | | | | |
| | | Delinquency | 62 | 6.90 | 8.13 | 200 | 6.08 | 7.00 | 55 | 7.02 | 8.20 | 0.51 | | | | | | |
| | Parent-report | SDQ Behavior Problems | 149 | 3.38 | 2.74 | 730 | 3.98 | 3.07 | 179 | 4.16 | 3.16 | 2.90 | | | | | | |
| | | Anxiety | 149 | 2.36 | 2.56 | 730 | 2.48 | 3.44 | 179 | 2.82 | 3.59 | 0.78 | | | | | | |
| | | Moods and Feelings | 149 | .54 | 1.28 | 729 | .77 | 2.04 | 179 | .64 | 1.36 | 1.13 | | | | | | |
| | | ADHD | 149 | 5.99 | 6.73 | 729 | 6.88 | 7.26 | 179 | 8.07 | 8.05 | 2.98 | | | | | | |
| | | Negative Symptoms | 149 | 2.37 | 3.02 | 727 | 2.99 | 4.09 | 179 | 3.22 | 4.16 | 1.93 | | | | | | |
| | | Callous unemotional traits | 148 | 17.69 | 8.56 | 730 | 19.37 | 9.30 | 179 | 18.34 | 8.95 | 2.60 | | | | | | |
| | | Autism | 149 | 24.89 | 11.87 | 729 | 25.09 | 10.98 | 179 | 25.48 | 11.01 | 0.08 | | | | | | |

*Note: N*= sample size; *M*=Mean; *SD* = standard deviation; $R^2$ = variance explained; *O* = on-time pubertal group; *E* = early pubertal timing group; *L* = late pubertal timing group; *NS* = non-significant.

***Table S5.5*** - Analysis of variance and associated post-hoc comparisons looking at early, on-time and late pubertal timing in girls at age 16 and behavioral and psychiatric problems concurrently at age 16. Analysis was conducted by randomly selecting one twin per pair.

| | | Measure | Early | | | On-time | | | Late | | | *F* | Early vs. On time | | | On time vs. Late | | |
|---|---|---|---|---|---|---|---|---|---|---|---|---|---|---|---|---|---|---|
| | | | *N* | *M* | *SD* | *N* | *M* | *SD* | *N* | *M* | *SD* | | t | *d* | $R^2$ | t | *d* | $R^2$ |
| Age 16 | Self-report | Behavior Problems | 178 | 8.99 | 4.94 | 737 | 9.29 | 4.94 | 148 | 10.06 | 4.74 | 2.02 | | | | | | |
| | | Anxiety | 178 | 9.03 | 6.10 | 738 | 8.92 | 5.82 | 149 | 10.43 | 6.84 | 3.96* | -2.52* | -0.36 | 0.18 | | | |
| | | Moods and Feelings | 178 | 4.53 | 5.55 | 739 | 4.22 | 4.63 | 148 | 4.20 | 4.76 | 0.29 | | | | | | |
| | | ADHD | 69 | 4.75 | .89 | 307 | 4.81 | .85 | 66 | 4.76 | .74 | 0.14 | | | | | | |
| | | Callous unemotional traits | 68 | 17.18 | 6.36 | 306 | 17.33 | 6.82 | 66 | 18.28 | 7.01 | 0.64 | | | | | | |
| | | Autism | 177 | 11.48 | 6.03 | 737 | 11.15 | 5.49 | 149 | 11.78 | 6.04 | 0.87 | | | | | | |
| | | Paranoid Checklist | 178 | 12.91 | 11.64 | 739 | 12.07 | 10.38 | 148 | 12.40 | 11.50 | 0.44 | | | | | | |
| | | Anomalous Perceptions | 178 | 4.38 | 5.78 | 738 | 4.49 | 5.88 | 148 | 5.94 | 8.12 | 3.59* | | | | -2.07* | -0.30 | 0.15 |
| | | Grandiosity & Delusion | 178 | 4.69 | 4.12 | 739 | 4.81 | 4.17 | 148 | 4.89 | 4.31 | 0.12 | | | | | | |
| | | Cognitive Disorganisation | 178 | 3.89 | 2.72 | 735 | 4.29 | 2.81 | 149 | 4.65 | 2.78 | 3.11* | *NS* | | | | | |
| | | Hedonia | 178 | 35.14 | 7.88 | 739 | 35.83 | 7.27 | 149 | 35.78 | 7.75 | 0.65 | | | | | | |
| | | Introvertive Anhedonia | 178 | .92 | 1.15 | 736 | 1.03 | 1.25 | 149 | 1.36 | 1.34 | 5.57** | | | | -2.75** | -0.39 | 0.19 |
| | | Eating problems | 69 | 4.11 | 2.09 | 306 | 3.74 | 1.92 | 64 | 3.79 | 1.98 | 1.07 | | | | | | |
| | | Peer Victimisation | 220 | 2.87 | 3.12 | 894 | 3.00 | 3.08 | 191 | 3.32 | 3.33 | 1.15 | | | | | | |
| | | Sleep | 109 | 8.46 | 4.20 | 433 | 8.29 | 4.88 | 83 | 9.06 | 5.29 | 0.94 | | | | | | |
| | | Substance use | 109 | 2.74 | 2.21 | 432 | 2.96 | 2.59 | 83 | 2.86 | 2.42 | 0.45 | | | | | | |
| | | Delinquency | 69 | 3.58 | 5.70 | 307 | 4.28 | 6.69 | 66 | 5.42 | 7.42 | 1.25 | | | | | | |
| | Parent-report | SDQ Behavior Problems | 178 | 2.90 | 2.89 | 735 | 2.94 | 2.51 | 148 | 3.06 | 2.55 | 0.13 | | | | | | |
| | | Anxiety | 178 | 3.65 | 4.22 | 735 | 3.86 | 4.14 | 148 | 3.90 | 3.85 | 0.17 | | | | | | |

| | | | | | | | | | | | | | | | | | | |
|---|---|---|---|---|---|---|---|---|---|---|---|---|---|---|---|---|---|---|
| | | Moods and Feelings | 178 | .96 | 2.51 | 734 | .99 | 2.23 | 148 | .87 | 2.14 | 0.19 | | | | | | |
| | | ADHD | 177 | 4.22 | 6.17 | 735 | 4.46 | 5.40 | 148 | 5.13 | 5.72 | 0.96 | | | | | | |
| | | Negative Symptoms | 178 | 1.96 | 3.10 | 735 | 2.15 | 3.22 | 148 | 2.00 | 2.60 | 0.36 | | | | | | |
| | | Callous unemotional traits | 178 | 13.66 | 7.23 | 734 | 15.06 | 8.02 | 148 | 15.76 | 8.15 | 3.15* | 2.11* | 0.13 | 0.06 | | | |
| | | Autism | 177 | 21.10 | 9.49 | 734 | 21.42 | 9.85 | 148 | 22.16 | 8.57 | 0.44 | | | | | | |

*Note:* *N*= sample size; *M*=Mean; *SD* = standard deviation; $R^2$ = variance explained; *O* = on-time pubertal group; *E* = early pubertal timing group; *L* = late pubertal timing group; *NS* = non-significant.

***Table S5.6*** - Analysis of variance and associated post-hoc comparisons looking at early, on-time and late pubertal timing in boys at age 16 and behavioral and psychiatric problems concurrently at age 16. Analysis was conducted by randomly selecting one twin per pair.

| | | Measure | Early | | | On-time | | | Late | | | *F* | Early vs. On time | | | On time vs. Late | | |
|---|---|---|---|---|---|---|---|---|---|---|---|---|---|---|---|---|---|---|
| | | | *N* | *M* | *SD* | *N* | *M* | *SD* | *N* | *M* | *SD* | | t | d | $R^2$ | t | d | $R^2$ |
| Age 16 | Self-report | Behavior Problems | 112 | 8.30 | 4.46 | 522 | 8.64 | 4.96 | 87 | 9.39 | 5.06 | 1.28 | | | | | | |
| | | Anxiety | 112 | 6.12 | 4.85 | 523 | 5.81 | 4.37 | 87 | 5.70 | 4.29 | 0.27 | | | | | | |
| | | Moods and Feelings | 112 | 2.63 | 3.57 | 522 | 2.88 | 3.70 | 87 | 3.19 | 3.98 | 0.55 | | | | | | |
| | | ADHD | 50 | 4.81 | .73 | 197 | 4.81 | .81 | 27 | 4.91 | .94 | 0.13 | | | | | | |
| | | Callous unemotional traits | 50 | 22.06 | 8.42 | 196 | 21.08 | 7.11 | 28 | 21.90 | 8.38 | 0.42 | | | | | | |
| | | Autism | 112 | 12.41 | 6.29 | 522 | 12.50 | 5.40 | 87 | 13.21 | 6.48 | 0.64 | | | | | | |
| | | Paranoid Checklist | 112 | 12.68 | 10.24 | 523 | 12.13 | 10.33 | 87 | 11.95 | 11.95 | 0.15 | | | | | | |
| | | Anomalous Perceptions | 112 | 4.34 | 5.66 | 523 | 4.31 | 5.77 | 87 | 5.18 | 7.10 | 0.81 | | | | | | |
| | | Grandiosity & Delusion | 112 | 5.03 | 4.12 | 523 | 6.12 | 4.56 | 87 | 6.48 | 5.46 | 3.10* | 2.49* | 0.38 | 0.19 | | | |
| | | Cognitive Disorganisation | 112 | 3.31 | 2.87 | 522 | 3.45 | 2.71 | 87 | 3.25 | 2.48 | 0.31 | | | | | | |
| | | Hedonia | 112 | 31.91 | 7.94 | 521 | 31.87 | 7.47 | 87 | 30.34 | 9.45 | 1.51 | | | | | | |
| | | Introvertive Anhedonia | 112 | 1.30 | 1.25 | 522 | 1.34 | 1.28 | 87 | 1.62 | 1.41 | 2.05 | | | | | | |
| | | Eating problems | 50 | 2.40 | 1.50 | 197 | 2.07 | 1.32 | 28 | 2.39 | 1.70 | 1.57 | | | | | | |
| | | Peer Victimisation | 153 | 3.97 | 3.11 | 646 | 4.11 | 3.34 | 113 | 4.08 | 3.18 | 0.12 | | | | | | |
| | | Sleep | 62 | 9.32 | 4.92 | 326 | 7.58 | 4.32 | 59 | 7.47 | 4.15 | 4.23* | -2.84** | -0.27 | 0.13 | | | |
| | | Substance use | 62 | 3.22 | 2.83 | 326 | 3.03 | 2.75 | 59 | 2.77 | 2.69 | 0.37 | | | | | | |
| | | Delinquency | 50 | 5.36 | 6.97 | 197 | 5.92 | 7.46 | 28 | 6.21 | 6.90 | 0.17 | | | | | | |
| | Parent-report | SDQ Behavior Problems | 114 | 3.46 | 2.57 | 523 | 3.71 | 2.92 | 87 | 4.48 | 3.34 | 3.39* | | | | -2.31* | -0.17 | 0.09 |
| | | Anxiety | 113 | 2.25 | 2.82 | 523 | 2.46 | 3.15 | 87 | 3.11 | 4.49 | 1.92 | | | | | | |
| | | Moods and Feelings | 114 | .52 | 1.69 | 522 | .74 | 1.96 | 87 | .66 | 1.84 | 0.63 | | | | | | |

| | | | | | | | | | | | | | | | | | | |
|---|---|---|---|---|---|---|---|---|---|---|---|---|---|---|---|---|---|---|
| | | ADHD | 114 | 6.30 | 7.38 | 523 | 6.23 | 6.95 | 87 | 7.64 | 7.50 | 1.58 | | | | | | |
| | | Negative Symptoms | 114 | 2.84 | 3.95 | 522 | 2.94 | 3.86 | 87 | 3.45 | 5.06 | 0.71 | | | | | | |
| | | Callous unemotional traits | 114 | 17.69 | 7.67 | 522 | 18.34 | 8.60 | 87 | 21.22 | 10.07 | 4.89** | | | | -2.53* | -0.49 | 0.24 |
| | | Autism | 114 | 24.06 | 10.61 | 523 | 25.56 | 11.16 | 87 | 26.81 | 11.99 | 1.57 | | | | | | |

*Note:* *N*= sample size; *M*=Mean; *SD* = standard deviation; $R^2$ = variance explained; *O* = on-time pubertal group; *E* = early pubertal timing group; *L* = late pubertal timing group; *NS* = non-significant.

***Table S5.7*** - Analysis of variance and associated post-hoc comparisons looking at early, on-time and late menarcheal timing in girls and behavioral and psychiatric problems concurrently and at age 14 and 16. Analysis was conducted by randomly selecting one twin per pair.

| | | Measure | Early | | | On-time | | | Late | | | *F* | Early vs. On time | | | On time vs. Late | | |
|---|---|---|---|---|---|---|---|---|---|---|---|---|---|---|---|---|---|---|
| | | | *N* | *M* | *SD* | *N* | *M* | *SD* | *N* | *M* | *SD* | | t | d | $R^2$ | t | d | $R^2$ |
| Age 11 | Self-report | Behavior Problems | 217 | 8.34 | 5.24 | 1134 | 8.06 | 5.08 | 225 | 8.00 | 5.48 | 0.27 | | | | | | |
| | | Moods and Feelings | 217 | 3.12 | 3.99 | 1140 | 3.06 | 3.82 | 225 | 2.96 | 3.97 | 0.06 | | | | | | |
| | | Victimisation | 218 | 6.49 | 6.74 | 1140 | 6.19 | 6.62 | 224 | 5.77 | 6.17 | 0.44 | | | | | | |
| | Parent-report | Behavior Problems | 217 | 6.54 | 4.97 | 1144 | 5.85 | 4.54 | 225 | 5.69 | 4.51 | 2.29 | | | | | | |
| | | Moods and Feelings | 216 | 1.71 | 2.85 | 1144 | 1.53 | 2.80 | 225 | 1.30 | 2.28 | 1.10 | | | | | | |
| | | Antisocial Personality | 217 | 6.83 | 4.17 | 1145 | 6.24 | 3.97 | 225 | 6.06 | 3.86 | 2.41 | | | | | | |
| | | Autism | 220 | 4.65 | 3.00 | 1147 | 4.00 | 2.79 | 226 | 4.06 | 2.71 | 4.82** | -3.10** | -0.16 | 0.08 | | | |
| | | ADHD | 216 | 7.95 | 7.07 | 1145 | 7.21 | 6.65 | 225 | 7.35 | 7.18 | 1.02 | | | | | | |
| Age 14 | Self-report | Victimisation | 194 | 6.80 | 6.67 | 999 | 6.85 | 6.55 | 214 | 6.53 | 6.23 | 0.10 | | | | | | |
| | | ADHD | 195 | 13.74 | 7.94 | 1002 | 12.74 | 7.76 | 215 | 11.61 | 7.33 | 3.84* | *NS* | | | | | |
| | Parent-report | Victimisation | 165 | 5.91 | 7.15 | 855 | 6.13 | 7.26 | 188 | 5.75 | 7.08 | 0.09 | | | | | | |
| | | Autism | 178 | 36.34 | 11.84 | 943 | 35.29 | 10.89 | 202 | 35.05 | 12.34 | 0.80 | | | | | | |
| | | Antisocial Personality | 181 | 7.43 | 4.99 | 948 | 6.85 | 4.58 | 201 | 6.47 | 4.59 | 1.99 | | | | | | |
| | | ADHD | 181 | 7.24 | 7.58 | 951 | 6.40 | 6.49 | 202 | 6.80 | 7.53 | 1.81 | | | | | | |
| Age 16 | Self-report | Behavior Problems | 208 | 9.67 | 4.93 | 1073 | 9.55 | 4.97 | 212 | 9.48 | 5.29 | 0.07 | | | | | | |
| | | Anxiety | 208 | 9.55 | 6.26 | 1076 | 9.25 | 6.10 | 212 | 8.91 | 5.89 | 0.62 | | | | | | |
| | | Moods and Feelings | 208 | 4.13 | 4.54 | 1075 | 4.45 | 5.03 | 213 | 3.72 | 4.32 | 2.31 | | | | | | |
| | | ADHD | 56 | 4.68 | .83 | 392 | 4.75 | .85 | 68 | 4.88 | .85 | 0.81 | | | | | | |
| | | Callous unemotional traits | 56 | 16.58 | 6.08 | 390 | 17.75 | 6.74 | 68 | 17.26 | 6.42 | 0.86 | | | | | | |

| | | | | | | | | | | | | | | | | | | |
|---|---|---|---|---|---|---|---|---|---|---|---|---|---|---|---|---|---|---|
| | | Autism | 208 | 12.03 | 5.89 | 1074 | 11.45 | 5.69 | 212 | 11.49 | 6.08 | 0.85 | | | | | | |
| | | Paranoid Checklist | 208 | 13.13 | 10.87 | 1076 | 12.77 | 10.95 | 212 | 10.60 | 9.89 | 4.06* | | | | 2.70** | 0.14 | 0.07 |
| | | Anomalous Perceptions | 208 | 5.47 | 7.38 | 1075 | 4.72 | 5.93 | 212 | 4.75 | 6.67 | 1.25 | | | | | | |
| | | Grandiosity & Delusion | 208 | 5.45 | 4.70 | 1076 | 4.68 | 4.06 | 212 | 4.80 | 4.55 | 3.02* | -2.46** | -0.13 | 0.06 | | | |
| | | Cognitive Disorganisation | 208 | 4.36 | 2.79 | 1072 | 4.32 | 2.84 | 212 | 4.18 | 2.82 | 0.31 | | | | | | |
| | | Hedonia | 208 | 35.32 | 8.13 | 1076 | 35.49 | 7.26 | 213 | 36.11 | 6.91 | 0.67 | | | | | | |
| | | Introvertive Anhedonia | 208 | 1.33 | 1.41 | 1073 | 1.04 | 1.23 | 212 | 1.10 | 1.23 | 4.45* | -2.69** | -0.33 | 0.16 | | | |
| | | Eating problems | 55 | 4.09 | 1.95 | 390 | 3.85 | 1.95 | 68 | 3.59 | 1.84 | 0.97 | | | | | | |
| | | Peer Victimisation | 185 | 2.92 | 3.06 | 927 | 3.18 | 3.20 | 209 | 2.65 | 2.91 | 2.61 | | | | | | |
| | | Sleep | 152 | 8.96 | 4.89 | 684 | 8.62 | 4.97 | 146 | 8.16 | 4.79 | 1.32 | | | | | | |
| | | Substance use | 152 | 2.97 | 2.75 | 684 | 2.70 | 2.51 | 145 | 2.73 | 2.64 | 1.16 | | | | | | |
| | | Delinquency | 56 | 4.23 | 5.88 | 392 | 4.49 | 6.42 | 68 | 4.56 | 7.73 | 0.05 | | | | | | |
| | Parent-report | SDQ Behavior Problems | 207 | 3.03 | 2.67 | 1073 | 3.14 | 2.79 | 211 | 2.91 | 2.62 | 0.55 | | | | | | |
| | | Anxiety | 208 | 4.23 | 4.48 | 1073 | 3.84 | 4.13 | 211 | 3.97 | 4.15 | 0.76 | | | | | | |
| | | Moods and Feelings | 208 | .87 | 2.14 | 1073 | 1.11 | 2.42 | 210 | .72 | 1.67 | 2.98 | | | | | | |
| | | ADHD | 207 | 5.06 | 6.37 | 1071 | 4.95 | 5.85 | 211 | 4.66 | 5.26 | 0.10 | | | | | | |
| | | Negative Symptoms | 208 | 2.49 | 3.65 | 1073 | 2.22 | 3.21 | 211 | 2.18 | 3.22 | 0.57 | | | | | | |
| | | Callous unemotional traits | 208 | 14.80 | 7.55 | 1073 | 15.43 | 8.39 | 210 | 15.42 | 8.31 | 0.56 | | | | | | |
| | | Autism | 207 | 23.10 | 9.80 | 1073 | 21.50 | 9.80 | 210 | 21.78 | 10.03 | 2.19 | | | | | | |

*Note:* $N$= sample size; $M$=Mean; $SD$ = standard deviation; $R^2$ = variance explained; $O$ = on-time pubertal group; $E$ = early pubertal timing group; $L$ = late pubertal timing group; $NS$ = non-significant

***Table S6*** - Estimated effect sizes for the contrasts between early vs on-time and late vs. on-time pubertal development from Graber et al 1997.

| | Girls | | | | | Boys | | | | |
|---|---|---|---|---|---|---|---|---|---|---|
| | Pubertal timing group standardized means | | | Planned contrasts | | Pubertal timing group standardized means | | | Planned contrasts | |
| | Early | On time | Late | Early vs On time (*d*) | Late vs. On time (*d*) | Early | On time | Late | Early vs On time (*d*) | Late vs. On time (*d*) |
| Daily hassles | 0.09 | -0.05 | 0.16 | *NS* | *NS* | 0.06 | 0.05 | 0.18 | *NS* | *-0.13* |
| Major life events | 0.13 | -0.03 | -0.03 | *NS* | *NS* | 0.17 | -0.05 | 0.06 | *0.22* | *NS* |
| Current depression | 0.18 | -0.05 | 0.03 | *0.23* | *NS* | 0.14 | -0.07 | 0.18 | *0.21* | *-0.25* |
| Internalizing behaviour | 0.11 | -0.04 | 0.09 | *NS* | *NS* | 0.09 | -0.09 | 0.28 | *NS* | *-0.37* |
| Externalizing behavior | 0.23 | -0.08 | -0.05 | *0.31* | *NS* | 0.05 | -0.04 | 0.04 | *NS* | *NS* |
| Negative cognitions | 0.11 | -0.03 | 0.04 | *NS* | *NS* | 0.04 | -0.05 | 0.14 | *NS* | *-0.19* |
| Attributional style | 0.14 | -0.03 | 0.05 | *0.17* | *NS* | -0.08 | -0.03 | 0.19 | *NS* | *-0.22* |
| Self-consciousness | 0.05 | -0.05 | 0.35 | *NS* | *-0.40* | -0.08 | -0.03 | 0.29 | *NS* | *-0.32* |
| Low self-esteem | 0.31 | -0.05 | -0.23 | *0.36* | *NS* | -0.06 | -0.01 | 0.05 | *NS* | *NS* |
| Emotional reliance | 0.09 | -0.03 | 0.07 | *NS* | *NS* | 0.21 | -0.11 | 0.28 | *0.32* | *-0.40* |
| Low future goals: academic | 0.12 | -0.01 | -0.26 | *NS* | *-0.27* | 0.06 | -0.01 | -0.03 | *NS* | *NS* |
| Low future goals: family | -0.01 | -0.01 | -0.03 | *NS* | *NS* | -0.20 | 0.04 | -0.03 | *0.16* | *NS* |
| Poor coping skills | 0.19 | -0.04 | -0.12 | *0.23* | *NS* | -0.07 | -0.03 | -0.18 | *NS* | *-0.21* |
| Low social support: family | 0.17 | -0.06 | 0.07 | *0.23* | *NS* | -0.04 | -0.03 | 0.06 | *NS* | *NS* |
| Low social support: friends | 0.13 | -0.04 | 0.07 | *0.17* | *NS* | -0.07 | 0.02 | -0.01 | *NS* | *NS* |
| Interpersonal: conflict with parents | 0.00 | -0.02 | 0.22 | *NS* | *-0.24* | 0.01 | -0.05 | 0.19 | *NS* | *-0.24* |
| Physical illness | 0.10 | -0.03 | -0.02 | *NS* | *NS* | 0.24 | -0.07 | 0.12 | *0.31* | *NS* |
| Hypomanic personality | 0.07 | -0.03 | 0.02 | *NS* | *NS* | 0.15 | -0.09 | 0.17 | *0.24* | *NS* |
| Days missed at school | 0.16 | -0.05 | -0.04 | *0.21* | *NS* | 0.12 | -0.06 | 0.12 | *NS* | *NS* |
| Late for school | 0.04 | -0.01 | 0.08 | *NS* | *NS* | 0.13 | -0.06 | 0.16 | *NS* | *0.22* |
| Failure to complete homework | 0.12 | -0.03 | -0.05 | *NS* | *NS* | 0.06 | -0.05 | 0.21 | *NS* | *0.11* |
| Poor self-rated health | 0.19 | -0.04 | -0.11 | *0.23* | *NS* | 0.06 | 0 | -0.06 | *NS* | *NS* |
| Lifetime no physical symptoms | 0.10 | -0.05 | 0.19 | *NS* | *NS* | 0.20 | -0.06 | 0.11 | *0.26* | *NS* |
| Current rate of tobacco use | 0.18 | -0.04 | -0.09 | *0.22* | *NS* | 0.23 | -0.03 | -0.11 | *0.26* | *NS* |

Note: (*d*) = Cohen's d effect sizes for the significant contrasts. These were estimated using the mean provided in the paper and assuming a standard deviation of 1 (scores had been standardized). However, as exact standard deviations were not provided, Cohen's d estimates should be interpreted with caution

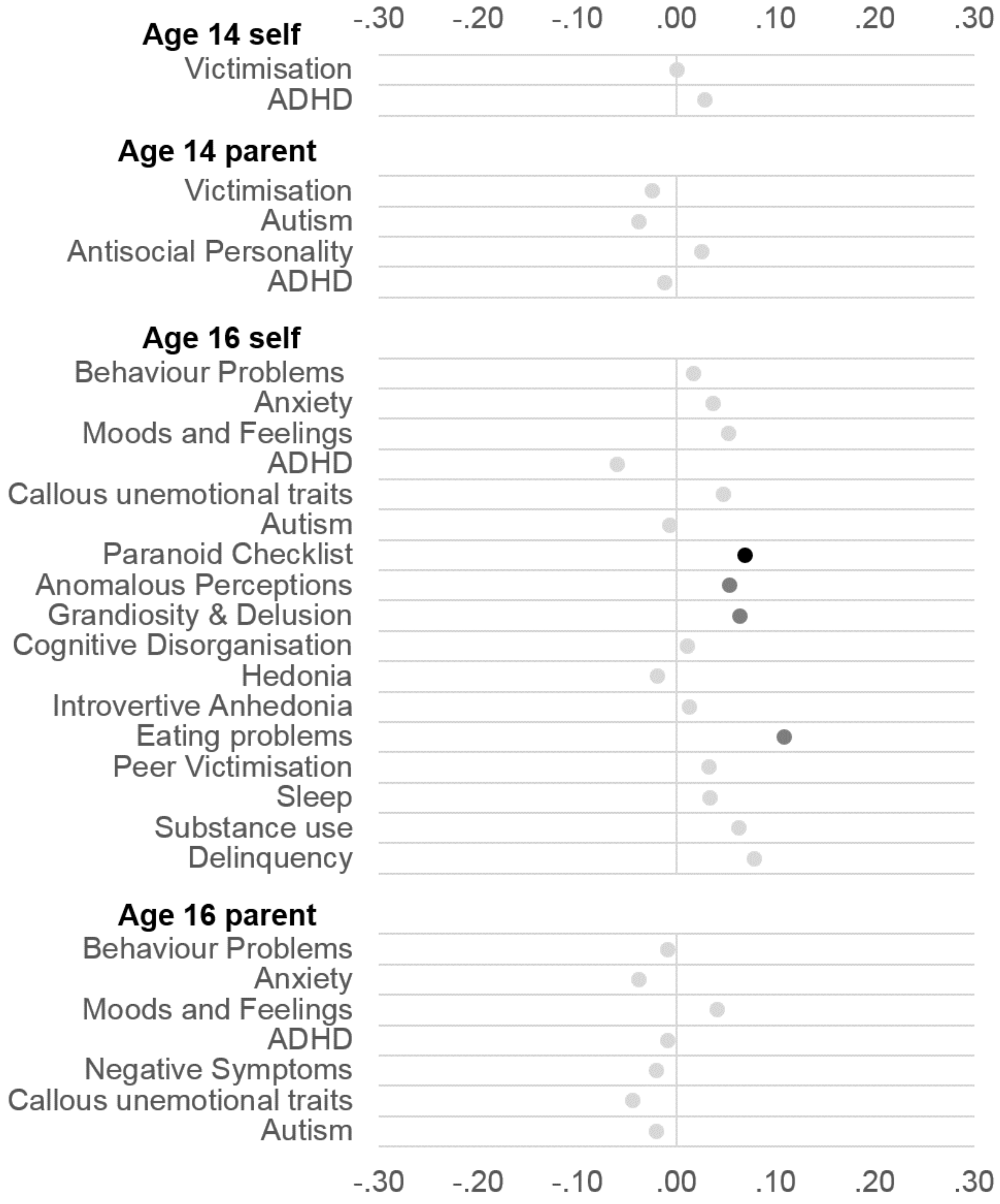


***Figure S1.1*** - Correlations between girls PDS score at age 14 and psychiatric and behavioral problems. Black dots indicate correlations significant at the .01, dark grey dots indicate correlations significant at the .05 level and light grey dots indicate non-significant correlations. Analyses was conducted by randomly selecting one twin per pair.

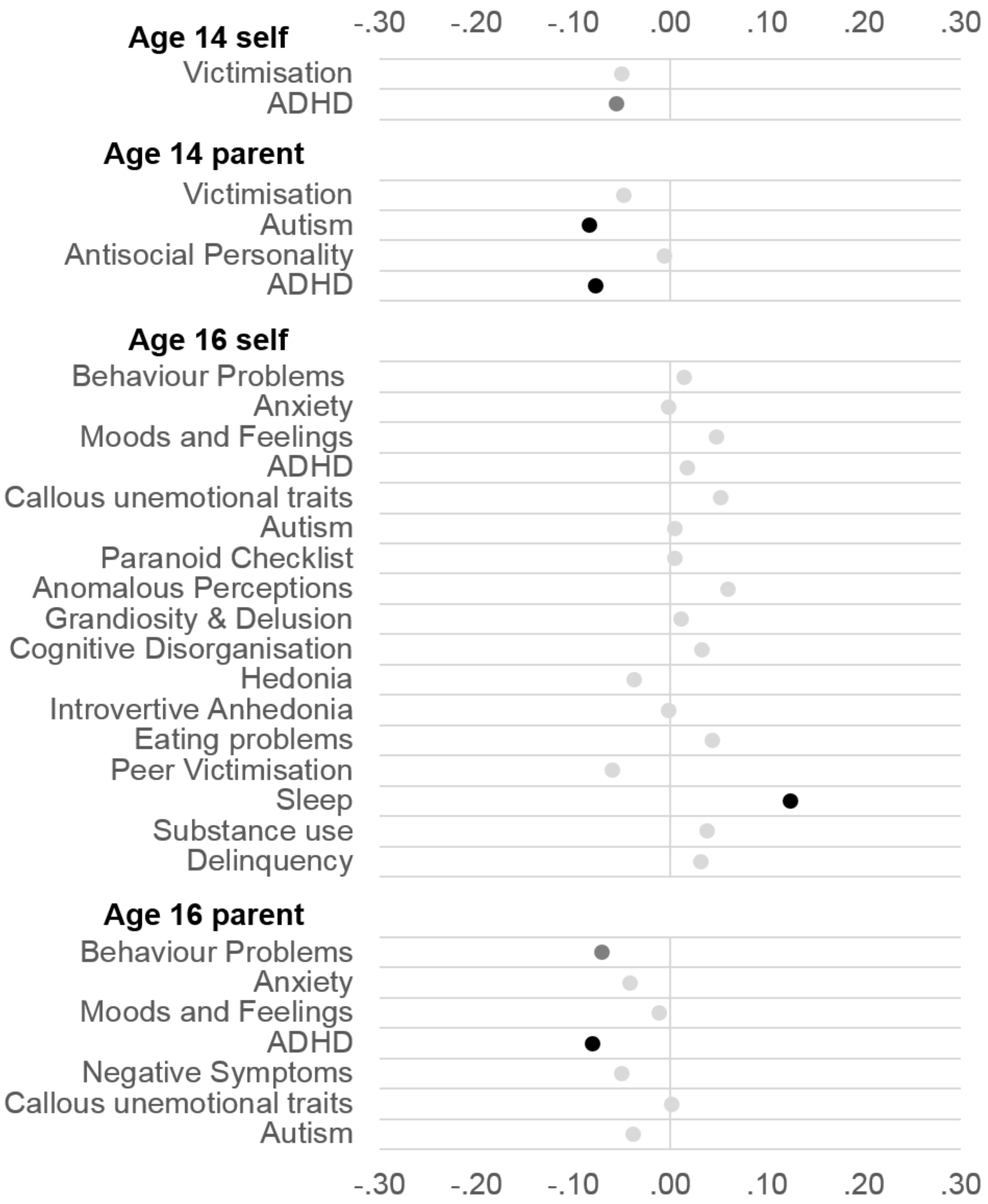


***Figure S1.2*** - Correlations between boys PDS score at age 14 and psychiatric and behavioral problems. Black dots indicate correlations significant at the .01, dark grey dots indicate correlations significant at the .05 level and light grey dots indicate non-significant correlations.

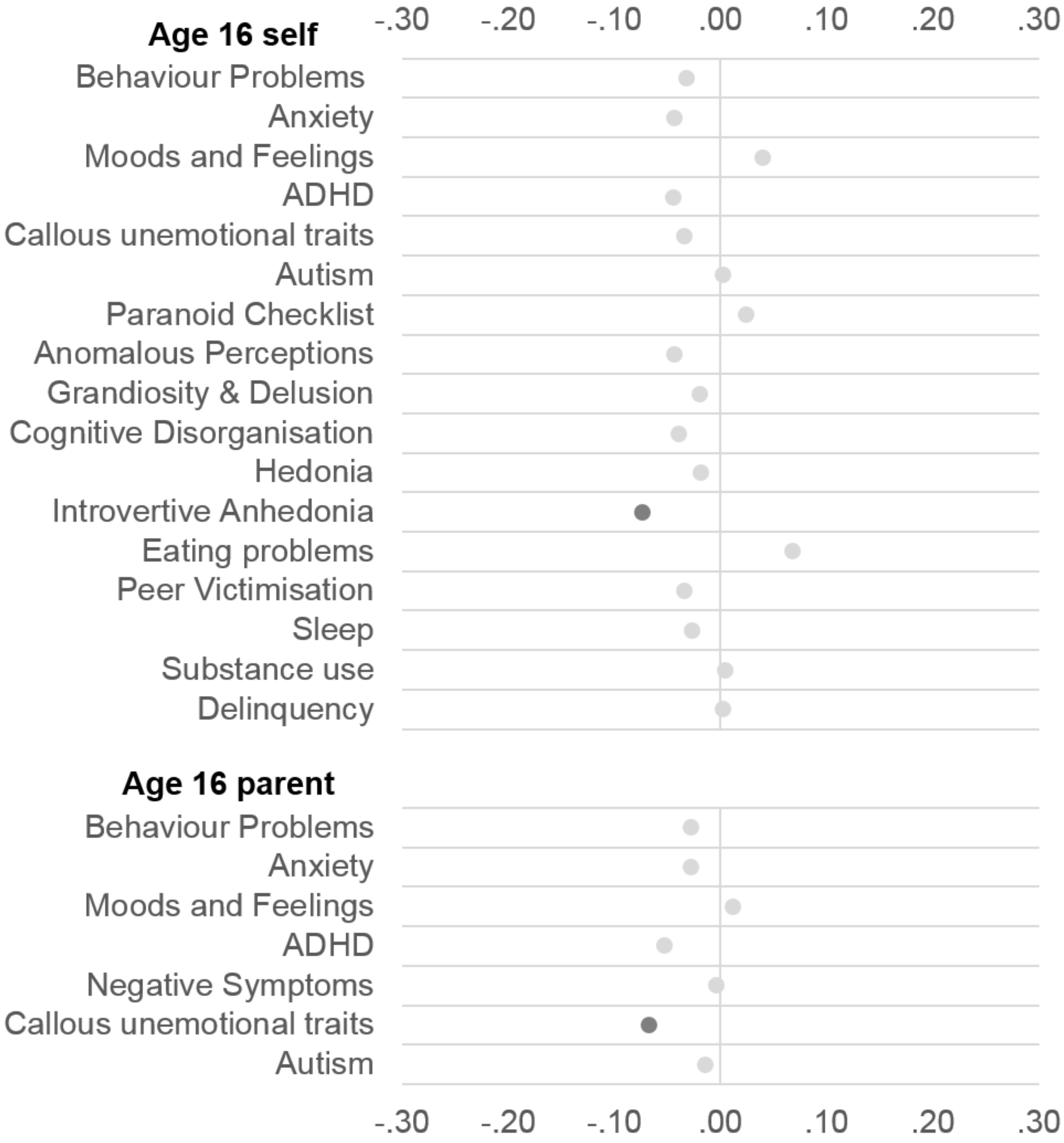


***Figure S1.3*** - Correlations between girls PDS score at age 16 and psychiatric and behavioral problems. Black dots indicate correlations significant at the .01, dark grey dots indicate correlations significant at the .05 level and light grey dots indicate non-significant correlations. Analyses was conducted by randomly selecting one twin per pair.

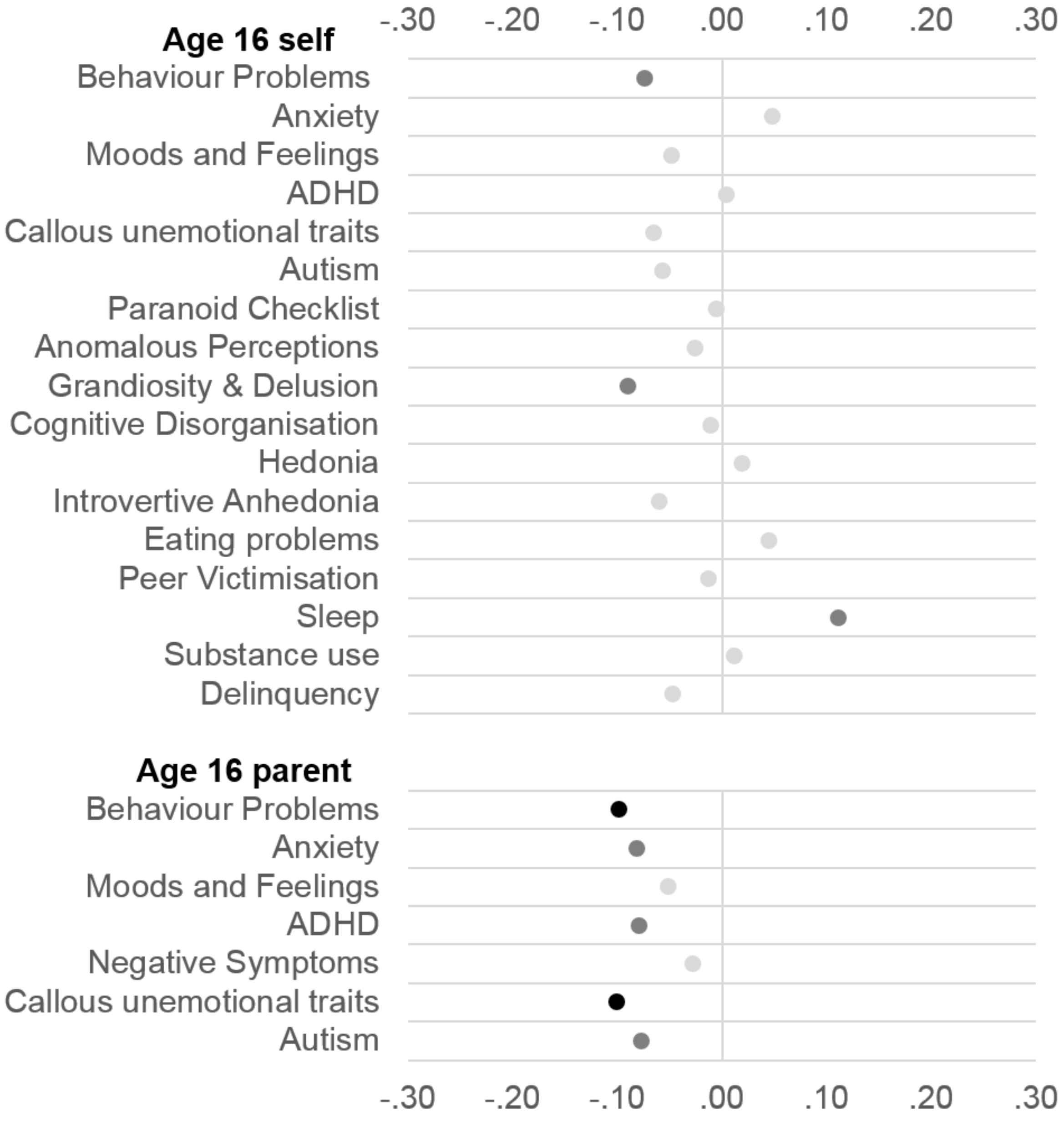


***Figure S1.4*** - Correlations between boys PDS score at age 16 and psychiatric and behavioral problems. Black dots indicate correlations significant at the .01, dark grey dots indicate correlations significant at the .05 level and light grey dots indicate non-significant correlations. Analyses was conducted by randomly selecting one twin per pair.
